# Coupling of Adhesion and Anti-Freezing Properties in Hydrogel Electrolytes for Low-Temperature Aqueous-Based Hybrid Capacitors

**DOI:** 10.1007/s40820-023-01229-9

**Published:** 2023-11-20

**Authors:** Jingya Nan, Yue Sun, Fusheng Yang, Yijing Zhang, Yuxi Li, Zihao Wang, Chuchu Wang, Dingkun Wang, Fuxiang Chu, Chunpeng Wang, Tianyu Zhu, Jianchun Jiang

**Affiliations:** 1 Institute of Chemical Industry of Forest Products, Chinese Academy of Forestry, Key Laboratory of Biomass Energy and Material, Jiangsu Province, Nanjing, 210042 Jiangsu People’s Republic of China; 2 Co-Innovation Center of Efficient Processing and Utilization of Forest Resources, Nanjing Forestry University, Nanjing, 210037 Jiangsu People’s Republic of China; 3 Energy Technologies Area, Lawrence Berkeley National Laboratory, Berkeley, CA 94720 USA; 4 Department of Materials Science and Engineering, Clemson University, Clemson, SC 29634 USA

**Keywords:** Interfacial adhesion, Anti-freezing, Hydrogel electrolytes, Low-temperature hybrid capacitors, Dynamic deformation

## Abstract

Solid-state zinc-ion capacitors are emerging as promising candidates for large-scale energy storage owing to improved safety, mechanical and thermal stability and easy-to-direct stacking. Hydrogel electrolytes are appealing solid-state electrolytes because of eco-friendliness, high conductivity and intrinsic flexibility. However, the electrolyte/electrode interfacial contact and anti-freezing properties of current hydrogel electrolytes are still challenging for practical applications of zinc-ion capacitors. Here, we report a class of hydrogel electrolytes that couple high interfacial adhesion and anti-freezing performance. The synergy of tough hydrogel matrix and chemical anchorage enables a well-adhered interface between hydrogel electrolyte and electrode. Meanwhile, the cooperative solvation of ZnCl_2_ and LiCl hybrid salts renders the hydrogel electrolyte high ionic conductivity and mechanical elasticity simultaneously at low temperatures. More significantly, the Zn||carbon nanotubes hybrid capacitor based on this hydrogel electrolyte exhibits low-temperature capacitive performance, delivering high-energy density of 39 Wh kg^−1^ at −60 °C with capacity retention of 98.7% over 10,000 cycles. With the benefits of the well-adhered electrolyte/electrode interface and the anti-freezing hydrogel electrolyte, the Zn/Li hybrid capacitor is able to accommodate dynamic deformations and function well under 1000 tension cycles even at −60 °C. This work provides a powerful strategy for enabling stable operation of low-temperature zinc-ion capacitors.

**Electronic supplementary material:**

The online version of this article (10.1007/s40820-023-01229-9) contains supplementary material, which is available to authorized users.

## Highlights

- A class of hydrogel electrolytes that couple high adhesion and anti-freezing properties is developed.
- Zn/Li hybrid capacitors based on the hydrogel electrolyte can tolerate low temperatures and accommodate dynamic deformations across a temperature range of 25 to − 60 °C.
- This work highlights an advancement for promoting next-generation energy storage system with low-temperature capability and mechanical durability.

**Electronic supplementary material:**

The online version of this article (10.1007/s40820-023-01229-9) contains supplementary material, which is available to authorized users.

## Graphical abstract

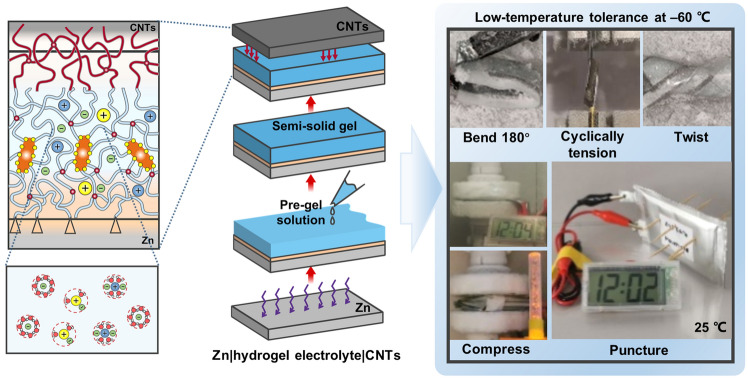

## Introduction

Metal-ion capacitors with hybrid configurations of a battery-type electrode and a capacitor-type electrode have emerged as a promising candidate for electrochemical energy storage, since they offer improved energy density without sacrificing their lifespan and power performance [1–8]. In particular, zinc (Zn)-ion capacitors are increasingly attractive due to the intrinsic merits of Zn in abundant resources, low redox potential (−0.76 V vs. standard hydrogen electrode) and high compatibility with water [4, 9–14]. However, many reported Zn-ion capacitors are based on conventional organic-based liquid electrolytes, which would result in potential safety concerns caused by the flammability of organic solvents and leakage of toxic liquid electrolytes [15, 16]. In contrast, solid-state electrolytes are rational alternatives for constructing solid-state Zn-ion capacitors because of their enhanced safety, mechanical and thermal stability, no leakage and easy-to-direct stacking [17–20]. Among various solid-state electrolytes, cost-efficient aqueous-based hydrogel electrolytes are favored for their eco-friendliness, high ionic conductivity and intrinsic flexibility that enables to accommodate mechanical deformations [21, 22]. At present, much progress of hydrogel electrolytes for Zn-ion capacitors has been made, mainly focusing on the fabrication of tough hydrogel electrolytes with high ionic conductivity [23–25]. Several recent works have successfully developed elastic hydrogel electrolytes/electrodes, such as the zwitterionic hydrogel electrolyte [26] and polyvinyl alcohol (PVA) hydrogel electrode [27], to achieve Zn-ion capacitors with exceptional flexibility and self-healing at the device level. Nevertheless, two key challenges should be addressed. First, compared with liquid electrolytes, solid-state hydrogel electrolytes commonly have an insufficient interfacial contact with electrodes, thus likely resulting in the detachment during cell fabrication and operation. Second, the high freezing point of water solvent easily induces hydrogel electrolytes to be frozen, hence limiting the ion transport and cell operation at low temperatures. Thus, it is highly desirable that how to design a robust adhesion of hydrogel electrolyte on electrode to form the strong electrolyte/electrode interface, and improve the anti-freezing ability of hydrogel electrolyte for high-performance solid-state Zn-ion capacitors.

Adhesion of hydrogels on engineering materials has been recently developed by typical four strategies [28]: (i) physical attachment between hydrogels and substrates, including electrostatic interactions, van der Waals interactions, hydrogen bonds and hydrophobic interactions [29, 30], (ii) covalent anchorage onto the substrates in combination with tough dissipative hydrogel matrices [31, 32], (iii) interfacial penetration of stitching polymers both into hydrogels and substrates [33, 34] and (iv) mechanical interlocking of hydrogels to porous substrates [35, 36]. Therefore, designing adhesion of hydrogel electrolyte on electrode requires two major considerations, including the cohesion of hydrogel matrix and the interfacial interaction between hydrogel electrolyte and electrode, which are synergistic contributions to robust adhesion [37]. On the other hand, the freezing of water arises mainly from the rearrangement of orderless water to ordered ice, which is driven by forming extra hydrogen bonds (H-bonds) at subzero temperatures [38, 39]. The use of highly concentrated salts in aqueous solutions is an efficient approach to suppress the water freezing, due to its ability to favor ion solvation configurations and break H-bonds in water [40, 41]. The previous reports have centered on the use of highly concentrated salts in anti-freezing hydrogel electrolytes, in an effort to reduce the freezing point of water solvent and improve low-temperature ionic conductivity [42, 43]. However, simply ascending salt concentrations in hydrogel electrolytes would strengthen the cation–anion interactions and bring about ions aggregation [41, 43], thus weakening the interactions between polymer chains and negatively affecting the mechanical robustness of polymer skeletons at low temperatures [44]. The compromised mechanical performance of such hydrogel electrolytes would lead to instability issues, such as short circuiting via external puncture [45, 46]. Therefore, how to modulate salt concentrations to balance the relationship between ion solvation configurations and ion interactions in water solvent should be further studied, thus obtaining an anti-freezing hydrogel electrolyte which meets the simultaneous requirement of excellent ionic conductivity and mechanical stability at low temperatures.

Here, we report a different type of hydrogel electrolytes for low-temperature Zn/Li hybrid capacitors, which couples high adhesion energy and impressive anti-freezing ability. The robust adhesion between hydrogel electrolyte and electrode is developed by the synergy of tough hydrogel matrix and chemical interfacial interaction, in which the former stiffens and strengthens the polymer skeleton by incorporating inorganic fillers, and the latter adheres to the electrode through chemically anchoring polymer networks. Meanwhile, benefiting from the cooperative solvation of ZnCl_2_ and LiCl hybrid salts, the hydrogel electrolyte simultaneously exhibits high ionic conductivity and mechanical elasticity at low temperatures. Using this hydrogel electrolyte, we demonstrate a solid-state Zn||carbon nanotubes (CNTs) hybrid capacitor, achieving impressive low-temperature capacitive performance and stable operation under dynamic deformations over a temperature range of 25 to −60 °C. This type of hydrogel electrolytes offers an alternative strategy for promoting the low-temperature aqueous-based energy storage system.

## Experimental Section

### Synthesis of Hydrogel Electrolytes

The hydrogel electrolyte was synthesized by polymerization of the soybean protein isolate-calcium sulfoaluminate-based (SPI-CSA-based) solution and the acrylamide-based (AAm-based) solution. Typically, the soybean protein isolate (SPI) dispersion solution was first made by dispersing 0.45-g SPI powders (Macklin) in 10-g deionized water at 95 °C for 4 h as our previously reported method [47]. The SPI-CSA-based solution was then prepared by dissolving 6.3-g calcium sulfoaluminate (CSA, Ca_4_(AlO_2_)_6_SO_4_) (Wuxi Yangshijin Construction), hybrid salts of 10.22-g ZnCl_2_ (Aladdin) and 7.63-g LiCl (Aladdin) in SPI dispersion solution at room temperature. The AAm-based solution was prepared by dissolving 3.15-g monomer acrylamide (AAm) (Aladdin), 9.45-mg cross-linker *N,N'*-methylenebisacrylamide (MBAA) (Sigma-Aldrich) and 94.5-mg thermal initiator ammonium persulfate (APS) (Sigma-Aldrich) in 5-g deionized water at room temperature. Both prepared solutions were homogeneously mixed to be a precursor solution. The degassed precursor solution was then polymerized at 60 °C for 3 h to synthesize the hydrogel electrolyte (Fig. S1).

### Adhesion of Hydrogel Electrolytes on Electrodes

The surface of Zn metal negative electrode was treated with silane functionalization chemistry [31, 48]. Zn plate (> 99%) was first treated with oxygen plasma for 5 min (BSP-B1 Plasma, Nanjing Jiaboli) to activate the surface. The plasma-treated Zn plate was immersed into the silane solution, containing 100-mL deionized water, 10-μL acetic acid (Aladdin) and 2 wt% of 3-(trimethoxysilyl) propyl methacrylate (TMSPMA) (Aladdin), for 2 h at room temperature. The TMSPMA-grafted Zn plate was then washed with ethanol and completely dried before use.

The hydrogel electrolyte/Zn electrode was adhered by in situ copolymerization of hydrogel electrolyte on Zn metal surface. The degassed precursor solution was poured onto the TMSPMA-grafted Zn surface at 60 °C for 3 h to generate the chemically anchored polymer network, resulting in the adhesion between the hydrogel electrolyte and Zn electrode [49].

The hydrogel electrolyte/CNTs was adhered by in situ polymerization of hydrogel electrolyte on CNTs electrode. The CNTs paper (XFNANO) was adhered onto the carbon cloth with conductive adhesive to serve as the CNTs electrode (the mass loading of CNTs in the electrode is ~ 1.7 mg cm^−2^). The degassed precursor solution was initially poured into glass mold at 60 °C for 6 min to form semi-solid gel. The CNTs electrode was then placed onto the upper side of the semi-solid gel at 60 °C for 3 h to induce in situ cross-linking of AAm monomers along CNTs surface, thus forming the adhesion between hydrogel electrolyte and CNTs electrode.

The Zn/hydrogel electrolyte/CNTs adhesion was obtained by in situ formation of hydrogel electrolyte on the Zn metal and CNTs paper. The degassed precursor solution was first poured onto the functionalized Zn electrode at 60 °C for 6 min to form semi-solid gel. Subsequently, the CNTs electrode was introduced onto the upper side of semi-solid gel at 60 °C for 3 h. Thus, the hydrogel electrolyte was in situ adhered simultaneously onto the Zn and CNTs electrodes.

### Material Characterizations

The morphology of hydrogel electrolyte was observed by SEM (Regulus 8220, HITACHI) equipped with an energy-dispersive X-ray spectroscopic detector (Ultim Max 170, OXFORD). The three-dimensional tomography image was constructed by an X-ray microscope (Bruker Skyscan 1272). The cross-sectional morphologies of hydrogel electrolyte/electrode interfaces were characterized by Cryo-SEM (Quorum PP3000T, FEI Quanta 450). The details of adhesion tests, mechanical tests, electrochemical tests, simulation of peeling process and molecular dynamics (MD) simulations are seen in Supporting Experimental Section.

## Results and Discussion

### Designing Adhesion of Hydrogel Electrolytes on Electrodes

**Fig. 1:**
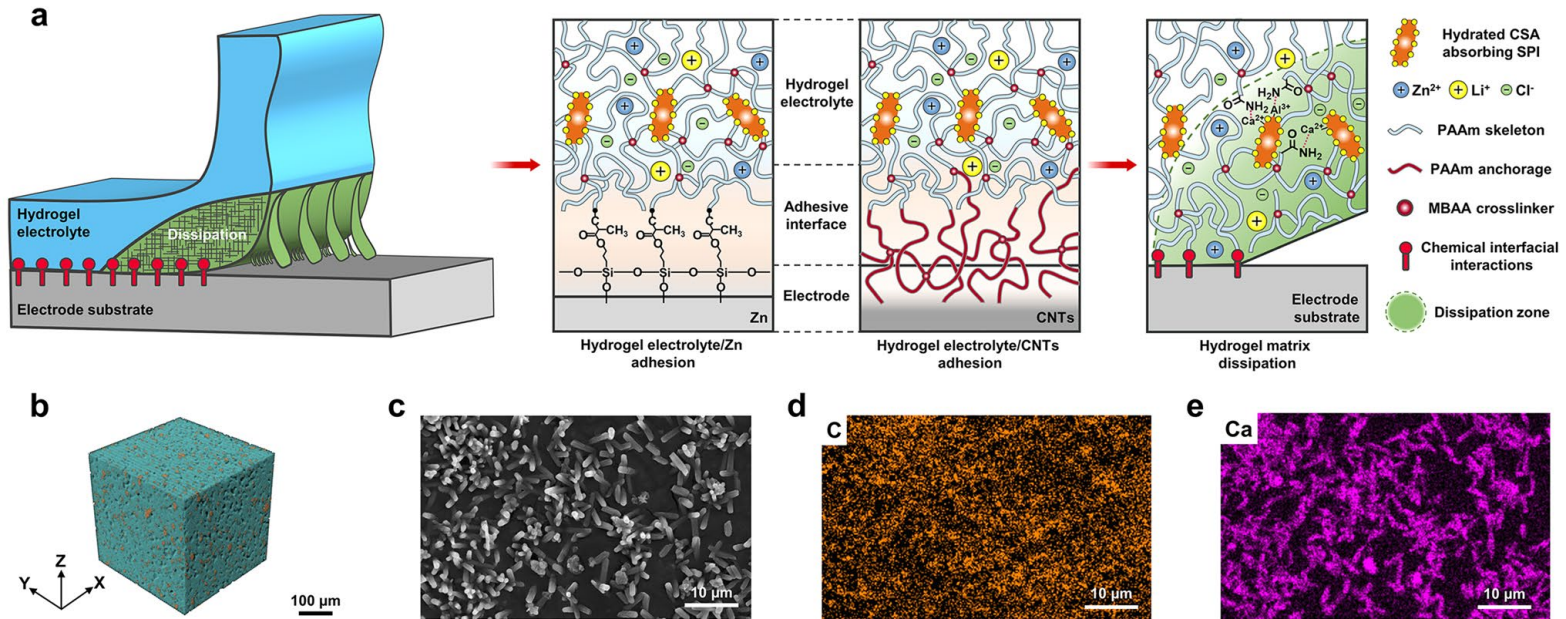
Design of adhering hydrogel electrolyte on electrode. **a** Schematic of the structure for hydrogel electrolyte and adhesive interface. The hydrogel electrolyte shows a sea–island structure, for which the inorganic phase of hydrated calcium sulfoaluminate (hydrated CSA, orange needle shape) is filled in the continuous phase of PAAm porous polymer network (blue line). The adhesive interface between hydrogel electrolyte and Zn is formed by chemically anchoring PAAm polymer network onto the silanized Zn metal surface. The adhesive interface between hydrogel electrolyte and CNTs is generated by penetrating PAAm polymer network (red line) into the porous CNTs. When a crack approaches, a process zone (light green region) effectively dissipates energy as the sliding of hydrated CSA facilitates tension to transmit and distribute among polymer chains. **b** Three-dimensional tomography of hydrogel electrolyte constructed by an X-ray microscope. **c** SEM image of hydrogel electrolyte. **d, e** EDS elemental mapping of the SEM image in **c**. (Color figure online)

The design principle for adhesion of hydrogel electrolytes on electrodes is based on two criteria: (i) The hydrogel electrolyte should have high toughness to resist cohesive failure [50, 51]. (ii) The hydrogel electrolyte should form strong interfacial interaction with electrode to combat adhesive failure [29, 33, 52]. To satisfy the first criterion, we engineer an organic–inorganic composite to stiffen and strengthen the polymer skeleton, creating tough matrix for the hydrogel electrolyte. For the second criterion, we chemically anchor polymer networks of hydrogel electrolyte onto the electrode surface by in situ polymerization, forming chemical interfacial interaction between hydrogel electrolyte and electrode. We propose that the robust adhesion of hydrogel electrolyte on electrode would be achieved by the synergy between tough matrix and chemical interfacial interaction, as illustrated in Fig. 1a.

With the guidance of the proposed design strategy, we fabricated the tough hydrogel electrolyte by polymerizing a precursor solution consisting primarily of SPI, CSA (Ca_4_(AlO_2_)_6_SO_4_), AAm as well as hybrid salts of ZnCl_2_ and LiCl at 60 °C (Fig. S1). In the polymerization process, inorganic crystals of hydrated CSA (Ca_6_Al_2_(SO_4_)_3_(OH)_12_) were first formed by the hydration of CSA [53]. SPI was selected as a bio-based pickering dispersant [54, 55] to stabilize the hydrated CSA crystals through electrostatic interactions between Ca^2+^/Al^3+^ of hydrated CSA and –COO^–^ of SPI nanoparticles (Fig. S2). AAm monomers were polymerized and covalently cross-linked with MBAA cross-linkers to form polyacrylamide (PAAm) network, which was followed by interacting with Ca^2+^/Al^3+^ of the hydrated CSA through metal coordination, thus obtaining the PAAm-CSA hydrogel electrolyte (Fig. S3). Three-dimensional tomography and scanning electron microscopy (SEM) images show that the hydrogel electrolyte has a typical sea–island structure, for which the inorganic phase of needle-shaped hydrated CSA with length ~ 3.6 μm is well filled in the continuous phase of PAAm porous polymer network (Figs. 1b, c and S4a, b). Energy-dispersive X-ray spectroscopy (EDS) elemental mapping images further verify that carbon (C) of the PAAm continuous phase presents a cross-linked polymer skeleton, while calcium (Ca), sulfur (S) and oxygen (O) of the hydrated CSA serving as inorganic fillers are incorporated into the polymer skeleton (Figs. 1d, e and S4c, d). At the same time, dissolved salt ions of Zn^2+^ and Cl^–^ are uniformly distributed in the porous channel of the polymer network, serving as an ion-conducting phase to produce ionic current via movements (Fig. S4e and f). The coordination between Ca^2+^/Al^3+^ of hydrated CSA and N of –CONH_2_ (on PAAm) contributes to the formation of sea–island structure. The combination of inorganic filled phase and organic continuous phase is expected to improve the mechanical performance of hydrogel electrolyte.

**Fig. 2:**
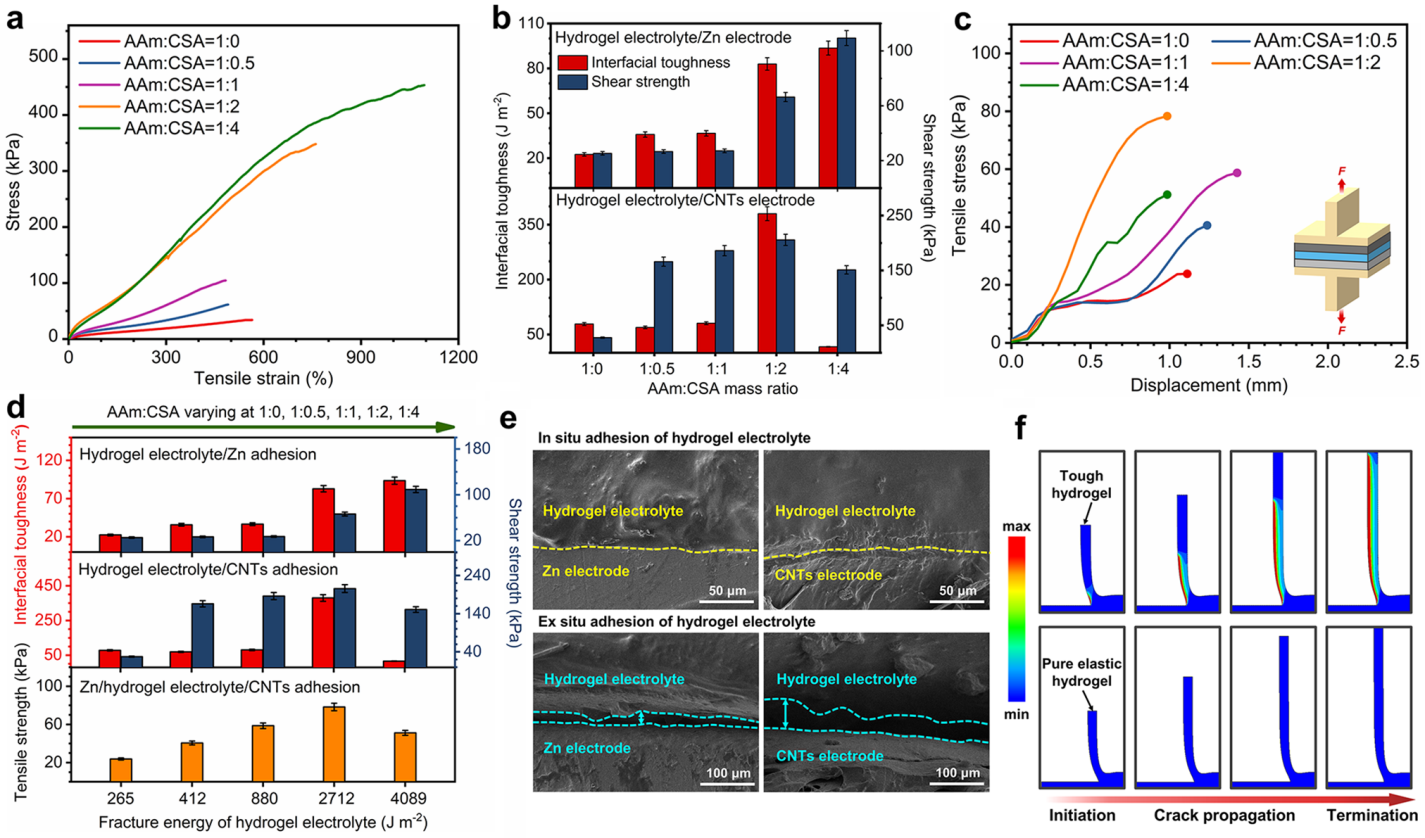
Adhesion performance of the hydrogel electrolyte.** a** Tensile stress versus strain curves of hydrogel electrolytes with different AAm:CSA mass ratios. **b** Interfacial toughness and shear strength for various hydrogel electrolyte/Zn hybrids and hydrogel electrolyte/CNTs hybrids. **c** Tensile stress versus displacement curves of various Zn/hydrogel electrolyte/CNTs hybrids. **d** Interfacial toughness, shear strength and tensile strength of hybrids against the fracture energy of hydrogel electrolyte. **e** Cross-sectional SEM images of hydrogel electrolyte/electrode interfaces with in situ adhesion and ex situ adhesion. **f** Simulated peeling process of the tough hydrogel and the pure elastic hydrogel from rigid substrates. The color indicates the energy dissipation in the hydrogel

To verify the design of organic–inorganic composite in mechanical enhancement, we synthesized a series of hydrogel electrolytes by varying the inorganic phase content (the AAm:CSA mass ratio ranges from 1:0 to 1:4; the corresponding CSA content increases from 0 to 25.69 wt%) and investigated their mechanical properties. When the AAm:CSA mass ratio increases from 1:0 to 1:2, Young’s modulus, fracture stress and fracture strain significantly increase. As the mass ratio further increases to 1:4, the Young’s modulus slightly decreases, whereas fracture stress and fracture strain both increase (Figs. 2a and S5). This result indicates that incorporation of hydrated CSA fillers is beneficial to improve the stiffness and strength of hydrogel electrolyte simultaneously. On the one hand, a certain content of high-modulus inorganic fillers partially confines the mobility of polymer chains, thus stiffening the PAAm skeleton. On the other hand, the sliding of inorganic fillers facilitates tension to transmit and distribute among polymer chains, thus strengthening the PAAm skeleton. However, a higher content of inorganic fillers would break the organic continuous phase and strongly confine the mobility of polymer chains, thus resulting in a negative effect on stiffness and strength [56]. Meanwhile, typical tension-release hysteresis loops of hydrogel electrolytes exhibit an increased trend of mechanical energy dissipation with increasing the CSA content (Fig. S6), which suggests that sliding of hydrated CSA crystals can effectively dissipate energy by transmitting it to polymer chains as a result of chemical bonding between the organic phase and inorganic phase. Because the design of organic–inorganic composite integrates contributions of high stiffness, high strength and effective energy dissipation [57], the fracture energy of hydrogel electrolyte reaches more than 2,700 J m^−2^ as the AAm:CSA mass ratio increases to 1:2 (Figs. S7 and S8), which enables the hydrogel electrolyte tough enough for strong adhesion [31, 48, 58].

We then evaluated the synergy between tough matrix and interfacial interaction by examining the adhesion performance of hydrogel electrolyte on non-porous Zn electrode and porous CNTs electrode. The adhesion of hydrogel electrolyte/Zn electrode was obtained by in situ copolymerizing the precursor solution on the silanized Zn metal surface. A functional silane, 3-(trimethoxysilyl) propyl methacrylate (TMSPMA), was first applied to modify Zn metal, forming the TMSPMA-grafted Zn metal surface [49, 59]. The precursor solution of hydrogel electrolyte was then in situ copolymerized with grafted TMSPMA, facilitating chemically anchored long-chain polymer network onto Zn surface (details on the modification and anchoring process seen in Experimental Section and Fig. S9a). The proposed mechanism of interactions between the TMSPMA-grafted Zn electrode and the hydrogel electrolyte is schematically illustrated in Fig. S10. We examined the adhesion of hydrogel electrolyte/Zn electrode with interfacial toughness (by 90-degree peel tests) and shear strength (by lap-shear tests) (Figs. S11a, c and S12). As the AAm:CSA mass ratio increases from 1:0 to 1:4, the interfacial toughness rises from 22.45 to 93.51 J m^−2^ and the shear strength rises from 25.31 to 109.34 kPa, showing an obvious growth with the incremental fracture energy of hydrogel electrolyte (Fig. 2b, d). Meanwhile, when the mass ratio is in the range of 1:0 ~ 1:4, the adhesion of hydrogel electrolyte/Zn occurs adhesive failure at the interface during peel tests (Movie S1), indicating that the interfacial interaction formed by chemical anchorage of hydrogel electrolyte on Zn metal is weaker than the fracture toughness of hydrogel electrolyte. These results reflect that the fracture toughness of hydrogel matrix and the chemical anchorage simultaneously determine the adhesion of hydrogel electrolyte on Zn electrode.

The adhesion of hydrogel electrolyte/CNTs electrode was acquired by in situ polymerizing the precursor solution along the CNTs surface. As the porous CNTs surface was permeable, the AAm-based solution could be penetrated into the surface and covalently cross-linked, forming chemical anchorage across the interface (details on the anchoring process seen in Experimental Section and Fig. S9b). We measured the adhesion of hydrogel electrolyte/CNTs electrode with interfacial toughness (by 180-degree peel tests) and shear strength (by lap-shear tests) (Figs. S11b, c and S13). When the AAm:CSA mass ratio increases from 1:0 to 1:2, the interfacial toughness rises from 78.48 to 380.09 J m^−2^ and the shear strength rises from 27.46 to 205.67 kPa, demonstrating a substantial improvement with the incremental fracture energy of hydrogel electrolyte (Fig. 2b, d). However, as the mass ratio increases to 1:4, both of the interfacial toughness and shear strength present an evident reduction (Fig. 2b, d). At the same time, when the mass ratio is in the range of 1:0 ~ 1:2, the adhesion of hydrogel electrolyte/CNTs undergoes cohesive failure with leaving a residual hydrogel layer during peel tests. Whereas the mass ratio reaches 1:4, it occurs adhesive failure at the interface (Movie S1). These results indicate that a certain content of CSA is beneficial to formulate tough hydrogel matrix and strong chemical anchorage onto CNTs simultaneously, whereas excessive CSA would hinder the penetration of AAm-based solution and result in inferior interfacial interaction. Therefore, the robust adhesion of hydrogel electrolyte/CNTs is closely correlated with high fracture toughness of hydrogel electrolyte and strong interfacial interaction.

To further investigate the relationship between fracture toughness of hydrogel electrolyte, interfacial interaction of hydrogel electrolyte with Zn electrode and interfacial interaction of hydrogel electrolyte with CNTs electrode, we discussed the adhesion performance of Zn/hydrogel electrolyte/CNTs. The adhesion of Zn/hydrogel electrolyte/CNTs was obtained by in situ formation of hydrogel electrolyte on Zn metal electrode and CNTs electrode (details on the anchoring process seen in Experimental Section). We measured the adhesion strength of Zn/hydrogel electrolyte/CNTs by tensile tests (Figs. 2c and S11d). As the AAm:CSA mass ratio increases from 1:0 to 1:2, the tensile strength rises from 23.84 to 78.32 kPa, along with adhesive failure occurring consistently at the hydrogel electrolyte/Zn interface (Fig. S14 and Movie S1). In contrast, as the mass ratio reaching 1:4, the tensile strength reduces to 51.19 kPa while the adhesive failure occurs at the hydrogel electrolyte/CNTs interface (Fig. S14 and Movie S1). The above results indicate that when the mass ratio is in the range of 1:0 ~ 1:2, the interfacial interaction of hydrogel electrolyte/Zn is the lowest, which decides the adhesion strength of Zn/hydrogel electrolyte/CNTs. When the mass ratio increases to 1:4, the interfacial interaction of hydrogel electrolyte/CNTs is the lowest, which determines its adhesion strength. At the same time, as the mass ratio reaching 1:2, the platform in the tensile stress–displacement curve disappears (Fig. 2c), suggesting that the elastic behavior in the hydrogel electrolyte is highest and the viscosity-induced stress-relaxation behavior is weakest. Thus, the AAm:CSA mass ratio of 1:2 is selected as the optimal organic–inorganic composition for the hydrogel electrolyte by considering both two contributions of tough cohesion and strong interfacial interaction. We also compared the cross-sectional interfaces with different adhesion ways of hydrogel electrolyte on Zn/CNTs electrode in Fig. 2e. The interface that was formed by ex situ adhesion of hydrogel electrolyte without chemical anchorage onto electrode surface shows an obvious gap between them. In contrast, the interface that was formed by in situ adhesion of hydrogel electrolyte shows a seamless contact between hydrogel electrolyte and Zn/CNTs electrode, forming a more stable interface, which confirms the key role of chemical anchorage in well-adhered interface. Therefore, the tough hydrogel electrolyte can achieve robust adhesion to electrode materials with high interfacial toughness (~ 83 J m^−2^ for Zn and ~ 380 J m^−2^ for CNTs) and high shear strength (66 kPa for Zn and ~ 206 kPa for CNTs), which is strong enough for creating stable electrolyte/electrode interface [19, 60, 61].

To better understand the synergistic effect between the tough hydrogel matrix and interfacial interaction, we simulated the peeling process of different hydrogels from rigid substrates using the finite element method. For peeling simulations (Fig. S15), the elastic property and toughness of the hydrogels were modeled as the Ogden hyperelastic material and Mullins effect, respectively [31, 48]. The interfacial interactions were prescribed by a layer of cohesive elements [31]. Figure 2f and Movie S2 demonstrate the simulated peeling process of the tough hydrogel and the pure elastic hydrogel from rigid substrates. Obviously, the tough hydrogel generates energy dissipation during peeling process, whereas the pure elastic hydrogel exhibits an elastic behavior without energy dissipation. At the same time, the simulated results show that the calculated interfacial toughness of the tough hydrogel is much higher than that of the pure elastic hydrogel (Fig. S16). Moreover, the interfacial toughness increases monotonically with the energy of interfacial interactions (Fig. S16). These simulated results further validate the significance of high toughness of the hydrogel matrix and strong interfacial interaction in obtaining robust adhesion of hydrogels on engineering solids.

### Investigating Anti-freezing Property of Hydrogel Electrolytes

**Fig. 3:**
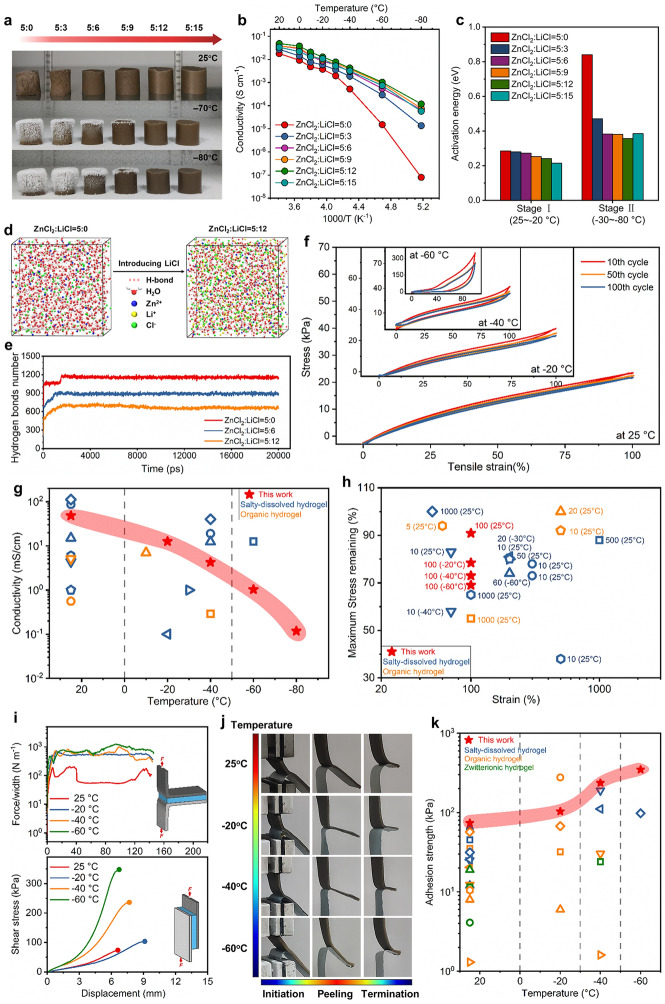
Anti-freezing property of the hydrogel electrolyte. **a** Optical images of hydrogel electrolytes with different ZnCl_2_:LiCl molality ratios at 25, −70 and −80 °C. **b** Ionic conductivities of different hydrogel electrolytes in the temperature range of 25 ~ −80 °C. **c** Activation energies of ionic conduction in different hydrogel electrolytes at normal temperature and low-temperature stages. **d** Snapshots of MD simulations in electrolytes with ZnCl_2_:LiCl ratios of 5:0 and 5:12. **e** H-bonds number versus time in three electrolytes obtained from MD simulations. **f** Tensile stress versus strain curves of the hydrogel electrolyte under 100% strain for 100 cycles at 25, − 20, − 40 and − 60 °C, respectively. **g, h** Comparison of our hydrogel electrolyte with previously-reported anti-freezing hydrogel electrolytes, in terms of conductivity versus operating temperature (**g**) and stress remaining versus tensile strain (**h**). A number of tension cycles are marked beside the corresponding symbols. Numbers in brackets represent the operating temperature. Details shown in Table S3. **i** Adhesion performance of the Zn/hydrogel electrolyte/CNTs at 25, −20, −40 and −60 °C. Force/width versus displacement curves for 180-degree peel tests (top) and shear stress versus displacement curves for lap-shear tests (bottom). **j** Optical images of the Zn/hydrogel electrolyte/CNTs during peeling process at different temperatures. **k** Comparison of the low-temperature adhesion performance of our hydrogel electrolyte with previously-reported anti-freezing hydrogels, in terms of adhesion strength versus operating temperature (details shown in Table S4)

We used the intermediate ZnCl_2_ concentration with a supporting LiCl as electrolyte salt in the hydrogel and investigated its effect on anti-freezing property of hydrogel electrolyte by modulating their concentrations. As shown in Fig. 3a, when the molality ratio of ZnCl_2_:LiCl increases from 5:0 to 5:15, the hydrogel electrolyte (AAm:CSA = 1:2) exhibits enhanced anti-freezing ability, maintaining unfrozen even at −80 °C. The ionic conductivities of hydrogel electrolytes with different molality ratios of ZnCl_2_:LiCl were tested in the temperature range of 25 ~ −80 °C (Fig. 3b). The specific ionic conductivities at different temperatures are summarized in Table S1. At a ZnCl_2_:LiCl ratio of 5:0, the ionic conductivity shows a fast decay as the temperature drops from 25 to −80 °C, resulting from the impeded Zn^2+^ transport in frozen hydrogel electrolyte at low temperatures. While adding LiCl, the reduction in ionic conductivities becomes slower with temperature dropping, indicating that the addition of LiCl prevents water solvent from freezing and ensures effective Zn^2+^ transport at low temperatures. At the same time, we find that when the temperature drops from 25 to −80 °C, the ionic conductivity of hydrogel electrolyte with a ZnCl_2_:LiCl ratio of 5:12 shows the smallest reduction and maintains the highest value at each temperature, which means that there exists a critical concentration of LiCl facilitating Zn^2+^ movement at low temperatures. To further reveal the temperature dependence of ionic conductivity, we calculated the activation energies of ionic conduction in hydrogel electrolytes according to Arrhenius equation [17, 41]. The activation energies are divided into two stages, including stage I of 25 ~ −20 °C and stage II of −30 ~ −80 °C (Fig. 3c and Table S2). At stage I, the activation energy decreases with LiCl concentration ascending, which indicates that the introduction of LiCl is beneficial to lower the energy barrier for Zn^2+^ transport at normal temperatures [43]. At stage II, the activation energy presents a dramatically decreasing and then slightly increasing trend with LiCl concentration ascending, reaching the lowest value of 0.357 eV at a ZnCl_2_:LiCl ratio of 5:12, which demonstrates that a critical addition of 12 m LiCl contributes to the lowest energy barrier for Zn^2+^ transport at low temperatures. In addition, the Raman spectra and fitting results reveal that the moderate addition of LiCl into the hydrogel electrolyte is beneficial to breaking the H-bonds among water molecules, thus efficiently suppressing ice nucleation and hindering water freezing (Fig. S17). The above results suggest that the moderate addition of LiCl can effectively suppress the formation of ice crystallization and improve the temperature independence of ionic conductivity in the hydrogel electrolyte, enabling favorable Zn^2+^ transport at low temperatures.

Toward revealing the mechanism of water freezing suppression and Zn^2+^ transport acceleration by introducing LiCl, we conducted molecular dynamics (MD) simulations of aqueous solutions with three ZnCl_2_:LiCl molality ratios (5:0, 5:6 and 5:12) to investigate the interactions among ions and water. The snapshots of MD simulations show a significant reduction of H-bonds upon introducing LiCl (Fig. 3d). In detail, when the ZnCl_2_:LiCl molality ratio increases from 5:0 to 5:12, the average number of H-bonds in water clusters reduces from ~ 1156 to ~ 673 (Fig. 3e), revealing that the addition of LiCl can obviously break water–water interactions. The average coordination number of Zn^2+^ with O (water) and Cl^−^ was calculated according to MD simulations (Fig. S18). As the LiCl molality adds from 0 to 12 m, the coordination number of Zn^2+^–O_W_ reduces from 4.2 to 3.7, and the Zn^2+^–Cl^−^ increases from 1.7 to 2.1, both of them showing slight changes. At the same time, the coordination number of Cl^−^–H_W_ maintains at ~ 7.5. This result indicates that the introduction of LiCl can validly alleviate Zn^2+^–Cl^−^ aggregations and ensure solvation of Zn^2+^ and Cl^−^ even at high concentrations. Therefore, the cooperative solvation of ZnCl_2_ and LiCl effectively prevents the water solvent from freezing and facilitates ions transport in the hydrogel electrolyte. We then performed low-temperature mechanical tests of the hydrogel electrolyte (AAm:CSA = 1:2) with a critical ZnCl_2_:LiCl ratio of 5:12. Benefiting from the excellent freezing resistance, the hydrogel electrolyte exhibits enhanced stretchability and toughness as the environmental temperature drops from 25 to −60 °C, in which the fracture strength increases from 348 to 643 kPa, and the fracture energy increases from 2712 to 3944 J m^−2^ (Fig. S19a and b). Meanwhile, the hydrogel electrolyte can endure compression at 80% strain even under −60 °C, with no attenuated compressive strength or cracking failure (Fig. S19c). More encouragingly, the hydrogel electrolyte can perfectly recover to its original shape after undergoing multi-cycle tensions or compressions in the temperature range of 25 ~ −60 °C (Figs. 3f, S20 and S21). In detail, at the temperature of 25 ~ −40 °C, the hydrogel electrolyte always maintains mechanical stability during 100 tension cycles at 100% strain, showing stable maximum stress of ~ 22.59 kPa (at 25 °C), ~ 25.37 kPa (at − 20 °C) and ~ 37.68 (at − 40 °C), low plastic deformation of ~ 6.45% (at 25 °C), ~ 10.90% (at − 20 °C) and ~ 17.19% (at − 40 °C) as well as high resilience of ~ 95% (at 25 °C), ~ 89% (at − 20 °C) and ~ 70% (at − 40 °C) (Figs. S20 and S22). When the temperature falls to −60 °C, despite that the hydrogel electrolyte presents enlarged maximum stress due to the reduced flexibility of polymer chains at low temperatures [62], it still demonstrates decent stretchable elasticity without brittle fracture or structural damage, in which the elastic recovery keeps ~ 52.67% during 100 tension cycles (Figs. S20 and S22). Furthermore, cyclic compressions of the hydrogel electrolyte at 80% strain also demonstrate that there are few significant deteriorations of compressive elasticity in the temperature range of 25 ~ −60 °C, maintaining ~ 63% height retention after 100 cycles even at − 60 °C (Figs. S21 and S23). Meanwhile, the differential scanning calorimetry (DSC) and dynamic mechanical analysis (DMA) results further confirm that the hydrogel electrolyte shows a phase transition around −116 °C, and is able to maintain mechanical stability above − 60 °C from the aspect of thermodynamics (Fig. S24). This excellent low-temperature mechanical stability indicates that the cooperative solvation of ZnCl_2_ and LiCl can restrain ions aggregations and maintain the strong interactions between polymer chains at such high concentration, thus preserving the elastic recoil ability of polymer skeleton at low temperatures. Even though the incorporation of hydrated CSA nanocrystals partially sacrifices the ionic conductivity of the hydrogel electrolyte, its contribution to stiffening and strengthening the hydrogel skeleton at low temperatures should also be valued (Fig. S25). It is noteworthy that our hydrogel electrolyte achieves a perfect combination of fantastic ionic conductivity and mechanical stability over a temperature range of 25 to − 60 °C, which has rarely been reported for any other anti-freezing hydrogel electrolytes (Fig. 3g, h and Table S3). In addition, our hydrogel electrolyte demonstrates excellent flame retardancy (Fig. S26). Consequently, the critical molality ratio of ZnCl_2_:LiCl = 5:12 was chosen as preferential hybrid salts for the application of anti-freezing hydrogel electrolyte.

We further evaluated the low-temperature adhesion performance of the Zn/hydrogel electrolyte/CNTs by 180-degree peel and lap-shear tests. When the temperature drops from 25 to −60 °C, the interfacial toughness rises from 75.85 to 836.96 J m^−2^, and the shear strength rises from 73.88 to 347.39 kPa, presenting an obvious enhancement with the decremental temperature (Figs. 3i and S27). Meanwhile, the peeling process of the Zn/hydrogel electrolyte/CNTs shows that both the hydrogel electrolyte and the adhered interface remain unfrozen in the temperature range of 25 ~ −60 °C (Fig. 3j). The enhanced adhesion of the Zn/hydrogel electrolyte/CNTs at low temperatures can be attributed to the increased toughness of the hydrogel electrolyte with the temperature dropping (Fig. S19b). Compared with previously reported adhesive hydrogels, our hydrogel electrolyte (AAm:CSA = 1:2, ZnCl_2_:LiCl = 5:12) achieves unprecedented adhesion to Zn/CNTs electrode across a temperature range of 25 to − 60 °C, which is critical for further applications of the Zn||CNTs hybrid capacitor at low temperatures (Fig. 3k and Table S4).

### Demonstrating Low-temperature Hybrid Capacitors

**Fig. 4:**
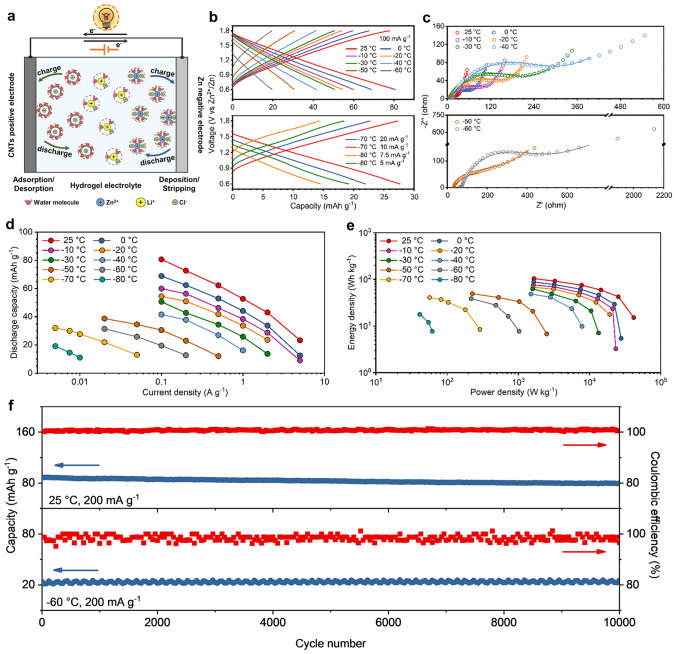
Low-temperature electrochemical performance of the Zn|hydrogel electrolyte|CNTs hybrid capacitor. **a** Schematic of the configuration and working mechanism of Zn|hydrogel electrolyte|CNTs hybrid capacitor. **b** Charge/discharge curves of the Zn||CNTs hybrid capacitor in the temperature range of 25 ~ −80 °C. **c** Temperature-dependent Nyquist plots of the hybrid capacitor. The solid lines correspond to the fitting equivalent circuits. **d** Discharge capacities of the hybrid capacitor with different current densities at various temperatures. **e** Ragone plots of the hybrid capacitor at various temperatures. The energy densities and power densities are normalized by the mass of active materials in CNTs electrodes. **f** Cycling performance of the hybrid capacitor at different temperatures. Capacity and Coulombic efficiency as a function of cycle number at 25 and −60 °C with 200 mA g^−1^

Motivated by strong adhesion of Zn/hydrogel electrolyte/CNTs and satisfying anti-freezing property of the hydrogel electrolyte, we investigated the use of the hydrogel electrolyte (AAm:CSA = 1:2 and ZnCl_2_:LiCl = 5:12) for the low-temperature Zn||CNTs hybrid capacitor. The configuration and working mechanism of the Zn/Li hybrid capacitor are schematically illustrated in Fig. 4a, presenting the hybridization of solvated Zn^2+^ deposition/stripping and solvated Cl^−^ adsorption/desorption. Before capacitor testing, we performed Zn plating/stripping tests in symmetric Zn cells to explore the Zn compatibility with hydrogel electrolyte. We assembled two types of symmetric Zn||Zn cells, in which the one is fabricated by directly stacking the hydrogel electrolyte between Zn electrodes (defining as “the Zn||Zn cell with ex situ adhesion”), the other is fabricated by in situ polymerizing the hydrogel electrolyte on Zn electrodes (defining as “the Zn||Zn cell with in situ adhesion”). As shown in Fig. S28a, compared with the cell with ex situ adhesion, the cell with in situ adhesion shows obviously lower overpotential. Meanwhile, the cycled Zn anode with in situ adhesion keeps smooth and flat on the surface, showing a dendrite-free morphology after 40 cycles (Fig. S28b and c). Even though the symmetric Zn cell with in situ adhesion shows increased overpotential with the temperature dropping, it remains working even at − 60 °C (Fig. S28d). These results suggest that the hydrogel electrolyte with strong adhesion and high anti-freezing properties favors Zn reversibility and compatibility, enabling stable Zn plating/stripping cycles across a temperature range of 25 to − 60 °C. In addition, the electrochemical stability window of the hydrogel electrolyte in Zn||Pt cell shows an oxidation wave over 1.8 V (Fig. S28e). This enhancement of oxidative stability is attributed to the cooperative solvation of ZnCl_2_ and LiCl, resulting in the scarcity of free water molecules and suppression of water decomposition. Electrochemical performance of the Zn|hydrogel electrolyte|CNTs hybrid capacitor containing a Zn metal negative electrode and a CNTs positive electrode was tested at different environmental temperatures. As shown in Figs. 4b and S29, when the temperature drops from 25 to −60 °C, the Zn||CNTs hybrid capacitor delivers the discharge capacity of 81, 69, 54, 42 and 20 mAh g^−1^ with the current density of 100 mA g^−1^ at 25, 0, −20, −40 and −60 °C, respectively. Even if the temperature falls to −80 °C, the hybrid capacitor still keeps working and displays capacity of 19 mAh g^−1^ at 5 mA g^−1^. Notably, the galvanostatic charge/discharge (GCD) curves maintain symmetric triangular shapes at different temperatures (Fig. 4b), presenting effective capacity output [63]. Meanwhile, the cyclic voltammetry (CV) curves in the scan rates of 0.4 ~ 1.0 mV s^−1^ show quasi-rectangular shapes at 25, −20 and −60 °C (Fig. S30), indicating the stable and reversible capacitive behavior of the hybrid capacitor at low temperatures [64, 65], which is in accordance with the GCD results.

We further used electrochemical impedance spectroscopy (EIS) to analyze the effect of temperature dropping on the electrochemical process. The temperature-dependent Nyquist plots of Zn||CNTs hybrid capacitor (Fig. 4c) and the fitting equivalent circuit results (Fig. S31a and Table S5) show that with the temperature dropping from 25 to −60 °C, the internal resistance (*R*_*s*_) slightly increases, which may be attributed to the fact that the anti-freezing hydrogel electrolyte is beneficial to ions transport at low temperatures. In contrast, the charge-transfer resistance (*R*_ct_) presents a significant increase, which could indicate that the charge-transfer kinetics suffers more at low temperatures. The relationship between log(1/*R*_ct_) and 1000/T shows a linear trend (Fig. S31b), indicating that the electrode/electrolyte interface is well maintained in the temperature range of 25 ~ −60 °C [66]. Next, the diffusion coefficients of Zn^2+^ (*D*_Zn2+_) at different temperatures are calculated according to the Warburg diffusion region (Fig. S31c and d) [67], and the corresponding log(*D*_Zn2+_) versus 1000/T plot is shown in Fig. S31e. Notably, the linear fitting of the relationship between log(*D*_Zn2+_) and 1000/T reveals that Zn^2+^ can still remain efficient diffusion in the electrode over the temperature from 25 to −60 °C. This EIS analysis suggests that it is the synergy of favorable ions transport, stable charge transfer at the interface and effective ions diffusion that enables the hybrid capacitor to work properly at low temperatures. In addition, the comparison of EIS results between the Zn||CNTs hybrid capacitors with ex situ adhesion and in situ adhesion further confirms that the robust interface formed by in situ adhesion significantly facilitates efficient charge transfer at the interface and ions diffusion in the electrode at low temperatures (Fig. S32 and Table S6), which is beneficial to improving the electrochemical performance of the hybrid capacitor across a temperature range of 25 ~ −60 °C. Discharge capacities of the hybrid capacitor with different current densities at various temperatures are summarized in Fig. 4d, exhibiting excellent rate capability. The energy densities and power densities of the hybrid capacitor at low temperatures were calculated based on the mass of active materials using Ragone-type plots (Fig. 4e and Table S7). The hybrid capacitor demonstrates a high-energy density of 104 Wh kg^−1^ at room temperature and maintains 39 Wh kg^−1^ at −60 °C, achieving low-temperature tolerance. In addition, the hybrid capacitor can cycle well at −60 °C over 10,000 cycles, with an average Coulombic efficiency of 98.4% and capacity retention of 98.7% at 200 mA g^−1^ (Figs. 4f and S33).

**Fig. 5:**
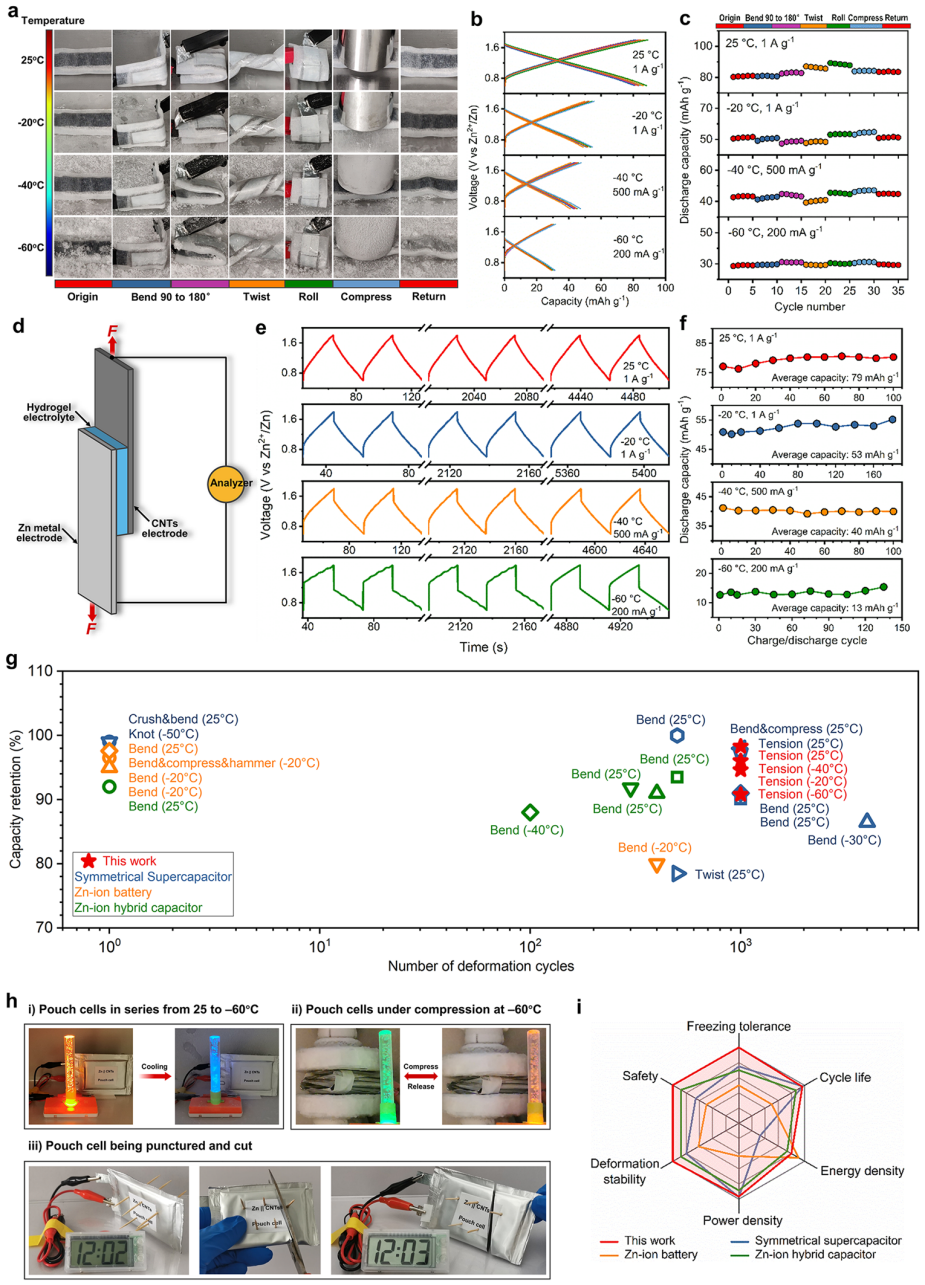
Correlating mechanical and capacitive behaviors of the Zn|hydrogel electrolyte|CNTs hybrid capacitor under dynamic deformations in the temperature of 25 ~ −60 °C. **a** Optical images of the Zn||CNTs hybrid capacitor under various deformations including bending, twisting, rolling and compressing at 25 ~ −60 °C. **b** Charge/discharge curves of the hybrid capacitor undergoing various mechanical deformations at 25, −20, −40 and −60 °C. **c** Corresponding changes in discharge capacities of the hybrid capacitor under various deformations at different temperatures. **d** Schematic of the Zn||CNTs hybrid capacitor under consecutive tensions. **e** Charge/discharge traces of the hybrid capacitor undergoing 1000 tension cycles of 20% strain at 25, −20, −40 and −60 °C.** f** Corresponding changes in discharge capacities of the hybrid capacitor during cyclic tensions at different temperatures. **g** Comparison of low-temperature deformation stability of our Zn||CNTs hybrid capacitor with previously-reported aqueous-based energy storage devices, in terms of capacity retention versus number of deformation cycles. Deformation types are noted beside the corresponding symbols. Numbers in brackets represent the operating temperature. Details shown in Table S8. **h** Demonstration of the Zn|hydrogel electrolyte|CNTs pouch-type cell providing power in harsh conditions. Pouch cells can function well at (i) −60 °C, (ii) under consecutive compressions as well as (iii) when being punctured and cut. **i** Comparison of electrochemical performance of our Zn||CNTs hybrid capacitor with previously-reported low-temperature aqueous-based energy storage devices, in terms of freezing tolerance, cycle life, energy density, power density, deformation stability and safety (details shown in Table S8)

To verify the interfacial stability, we tested the low-temperature electrochemical performance of the Zn||CNTs hybrid capacitor under dynamic deformations. As shown in Fig. 5a, the hybrid capacitor can support various mechanical deformations, such as bending, twisting, rolling and compressing, with no detachment or crack in the temperature range of 25 ~ −60 °C. Meanwhile, the corresponding charge/discharge traces at each temperature show the nearly overlapped shape under various deformations, demonstrating non-deteriorated discharge capacities at 25, −20, −40 and −60 °C, respectively (Fig. 5b and c). We further conducted charge/discharge tests for the hybrid capacitor under consecutive tensions at low temperatures. Figure 5d-f and Movie S3 demonstrate the charge/discharge behaviors of the hybrid capacitor being subjected to tension cycles at 20% strain in the temperature range of 25 ~ −60 °C. The corresponding tension-release traces are shown in Fig. S34. The hybrid capacitor shows a marginal decrease in capacity over 1000 tension cycles, maintaining stable capacity of 79, 53, 40 and 13 mAh g^−1^ at 25, −20, −40 and −60 °C, respectively (Fig. 5f). These results indicate that the hybrid capacitor is able to resist interfacial detachment and being frozen at low temperatures, operating properly even at −60 °C. More encouragingly, even at 30% tensile strain, the hybrid capacitor can still accommodate dynamic movements and operate well at low temperatures, presenting a minor capacity decrease of less than 16% over 1000 tension cycles in the temperature of 25 ~ −60 °C (Figs. S35, S36 and Movie S3). Thus, the coupling of well-adhered hydrogel electrolyte/electrode interface and remarkable anti-freezing hydrogel electrolyte allows our hybrid capacitor to operate well under cyclic deformations even at − 60 °C, which is more distinguished than existing low-temperature aqueous-based energy storage devices (Fig. 5g and Table S8). In addition, the Zn||CNTs hybrid capacitor based on the hydrogel electrolyte also demonstrates excellent high-temperature capacitive performance at 40 and 60 °C (Fig. S37), achieving distinguished wide-temperature adaptability across a wide-temperature range of −60 to + 60 °C.

Considering practical applications, we proceeded to fabricate the Zn|hydrogel electrolyte|CNTs pouch-type cell and investigate its operational performance. The fabrication process of Zn||CNTs pouch-type cell is illustrated in Fig. S38. Self-discharge results show that the pouch cell achieves high capacity retentions at low temperatures, remaining ~ 88%, ~ 91%, ~ 89% and ~ 77% of the initial capacity after resting 24 h at 25, −20, −40 and −60 °C, respectively (Fig. S39). As a demonstration, when the environmental temperature drops from 25 to −60 °C, a single pouch cell is able to continue powering an electronic watch, and five pouch cells in series enable a flash lamp to work well (Figs. S40(i), 5h(i) and Movie S4). More importantly, it is the coupling of robust adhesion and satisfying anti-freezing in hydrogel electrolyte that allows pouch cells to function properly even under consecutive compressions at −60 °C (Figs. S40(ii), 5h(ii) and Movie S5). Finally, the safety concern is verified by puncturing and cutting the pouch cell, in which the electronic watch remains working all the time (Fig. 5h(iii) and Movie S5). Therefore, this study strongly supports the stable operation of the hydrogel electrolyte-based Zn||CNTs pouch cells in extremely harsh conditions. Overall, our hybrid capacitor highlights an advancement in the key figures of merit for Zn-ion capacitor performance, including freezing tolerance, cycle life, energy density, power density, deformation stability and safety, which is more competitive than that for existing aqueous-based energy storage devices (Fig. 5i and Table S8).

## Conclusion

In summary, we develop a class of hydrogel electrolytes combining strong adhesion and impressive anti-freezing properties, which has been successfully used for low-temperature Zn-ion capacitors. The well-adhered interface of hydrogel electrolyte/electrode is generated by chemically anchoring the polymer network of tough hydrogel electrolyte onto the electrode surface, reaching unprecedented adhesion to Zn/CNTs electrode across a temperature range of 25 to − 60 °C. The moderate modulation of hybrid salts concentration enables the hydrogel electrolyte to favor fast Zn^2+^ transport and mechanical elasticity even at −60 °C, exhibiting excellent anti-freezing property. By coupling adhesion and anti-freezing properties in the hydrogel electrolyte, the Zn||CNTs hybrid capacitor utilizing this hydrogel electrolyte achieves appreciable low-temperature electrochemical performance, delivering high-energy density of 39 Wh kg^−1^ and excellent cycling behavior (with an average Coulombic efficiency of 98.4% and capacity retention of 98.7% over 10,000 cycles) at −60 °C. Meanwhile, the hybrid capacitor is able to handle dynamic deformations and maintain stable operation under 20 and 30% tension cycles across a temperature range of 25 ~ −60 °C. We expect that this type of hydrogel electrolytes would provide a pathway to address some interfacial and operational challenges of solid-state electrolytes for practical energy storage applications.

## Electronic supplementary material

Below is the link to the electronic supplementary material.

Supplementary file 1 (MP4 13704 kb)

Supplementary file 2 (MP4 2964 kb)

Supplementary file 3 (MP4 14717 kb)

Supplementary file 4 (MP4 14993 kb)

Supplementary file 5 (PDF 4826 kb)

Supplementary file 6 (MP4 14993 kb)
