# Supplementary material for: Coupling of Adhesion and Anti-Freezing Properties in Hydrogel Electrolytes for Low-Temperature Aqueous-Based Hybrid Capacitors
Source: Nanomicro Lett. 2023 Nov 20;16:22. doi: 10.1007/s40820-023-01229-9 (PMC10661583; doi:10.1007/s40820-023-01229-9)
Supplement: Supplementary file 5 — (PDF 4826 kb) [file 40820_2023_1229_MOESM5_ESM.pdf]

Supporting Information for

Coupling of Adhesion and Anti-Freezing Properties in Hydrogel Electrolytes for Low-Temperature Aqueous-Based Hybrid Capacitors

Jingya Nan[1, #], Yue Sun[1, #], Fusheng Yang[1], Yijing Zhang[1], Yuxi Li[1], Zihao Wang[1], Chuchu Wang[1], Dingkun Wang[1], Fuxiang Chu[1, 2], Chunpeng Wang[1, 2, *], Tianyu Zhu[3, 4, *], and Jianchun Jiang[1, 2, *]

[1] Institute of Chemical Industry of Forest Products, Chinese Academy of Forestry; Key Laboratory of Biomass Energy and Material, Jiangsu Province, Nanjing, Jiangsu 210042, P. R. China

[2] Co-Innovation Center of Efficient Processing and Utilization of Forest Resources, Nanjing Forestry University, Nanjing, Jiangsu 210037, P. R. China

[3] Energy Technologies Area, Lawrence Berkeley National Laboratory, Berkeley, CA 94720, USA

[4] Department of Materials Science and Engineering, Clemson University, Clemson, South Carolina 29634, USA

#Jingya Nan and Yue Sun are contributed equally to this work.

*Corresponding authors. E-mail: wangchunpeng@icifp.cn (Chunpeng Wang); tzhu2@clemson.edu (Tianyu Zhu); jiangjc@caf.ac.cn (Jianchun Jiang)

S1 Supporting Experimental Section

S1.1 Adhesion Tests

For the interfacial toughness tests, the hydrogel electrolyte/Zn samples with adhesion width of 2.5 cm were measured by the standard 90-degree peel test (ASTM D2861), and the hydrogel electrolyte/CNTs samples were measured by the standard 180-degree peel test (ASTM F2256). All tests were carried out using a universal testing machine (1 kN load cell, UTM 4304GD, Shenzhen SUNS) with a constant peeling rate of 50 mm min^{-1}. The interfacial toughness was determined by dividing the plateau force (for 90-degree peel test) or two times the plateau force (for 180-degree peel test) by the adhesion width. Polyester film (PET, thickness 0.2 mm) as a stiff backing was applied

onto hydrogel electrolyte with cyanoacrylate glue (Loctite, Henkel) to prevent its elongation along the peeling direction.

For the shear strength tests, the hydrogel electrolyte/Zn and hydrogel electrolyte/CNTs samples with an adhesion area of width 2.5 cm and length 1 cm were measured by the standard lap-shear test (ASTM F2255). All tests were carried out using a universal testing machine (1 kN load cell, UTM 4304GD, Shenzhen SUNS) with a constant tension rate of 50 mm min^{-1}. The shear strength was determined by dividing the maximum force by the adhesion area. PET film backing was applied onto hydrogel electrolyte with cyanoacrylate glue to prevent its elongation along the shear direction.

For the tensile strength tests, the Zn/hydrogel electrolyte/CNTs samples with an adhesion area of width 2.5 cm and length 2.5 cm were measured by the standard tensile test (ASTM F2258). All tests were carried out using a universal testing machine (1 kN load cell, UTM 4304GD, Shenzhen SUNS) with a constant tension rate of 50 mm min^{-1}. The tensile strength was determined by dividing the maximum force by the adhesion area.

The temperature-dependent adhesion performance of the Zn/hydrogel electrolyte/CNTs samples were measured with assistance of the universal testing machine equipped with an environmental chamber. The interfacial toughness tests (ASTM F2256) and shear strength tests (ASTM F2255) for the Zn/hydrogel electrolyte/CNTs samples were same as the above at different temperatures of 25, –20, –40, and –60 °C.

S1.2 Mechanical Tests

All mechanical properties of hydrogel electrolytes were performed on a universal testing machine (1 kN load cell, UTM 4304GD, Shenzhen SUNS) equipped with an environmental chamber (WGDN-7150S, Shenzhen SUNS). All temperature-dependent mechanical tests were conducted in an environmental chamber with desired temperatures.

For the fracture energy tests, the hydrogel electrolytes with a rectangular shape (of length 10 mm, width 20 mm and thickness 2 mm) were measured by the pure-shear tensile test. The tests were performed at room temperature with a constant tension rate of 50 mm min^{-1}. The fracture energy of hydrogel electrolytes was calculated from the tensile force-distance curves of the unnotched and notched samples according to the previously reported method [S1].

For the cyclic tension tests, the hydrogel electrolytes with a rectangular shape (of length 10 mm, width 20 mm and thickness 2 mm) were cyclically stretched to 100% strain and released to the original length. The tests were performed at desired temperatures with a constant tension rate of 50 mm min^{-1}.

For the cyclic compression tests, the hydrogel electrolytes with cylinder shape (of diameter 13 mm and height 10 mm) were cyclically compressed to 80% strain and released to the original height. The tests were performed at desired temperatures with a constant compression rate of 30 mm min^{-1}.

S1.3 Electrochemical Tests

Electrochemical performances of the hydrogel electrolytes and the assembled Zn||CNTs hybrid capacitors were measured on an electrochemical working station (CHI 760E) and a multichannel battery testing system (CH8-1A, Shenzhen Betta). All temperature-dependent electrochemical tests were conducted in an environmental chamber with

desired temperatures, which was controlled by Meiling refrigerator DW-HL100 (–86~–10 °C) or Suns environmental chamber WGDN-7150S (–70~150 °C). Ionic conductivities of hydrogel electrolytes were obtained from EIS tests of the Pt|hydrogel electrolyte|Pt symmetric cells. GCD and CV tests of the assembled Zn||CNTs hybrid capacitors were carried out in the voltage range of 0.6~1.8 V at current densities of 0.005~5 A g^{-1} and scan rates of 0.4~1 mV s^{-1}, respectively. EIS tests of the hybrid capacitors were performed in the frequency range of 10^5~0.01 Hz with 5 mV amplitude at an open-circuit voltage.

The temperature-dependent electrochemical behaviors of the hybrid capacitors under tension cycles were measured with assistance of the universal testing machine equipped with an environmental chamber. Each side of the Zn|hydrogel electrolyte|CNTs hybrid capacitor with an adhesion area of width 2.5 cm and length 2.5 cm was cyclically stretched to 20 or 30% tensile strain (with respect to the adhesion length) at different temperatures of 25, –20, –40, and –60 °C. At the same time, the corresponding charge/discharge curves of the hybrid capacitor were recorded to evaluate its capacitive stability. All specific capacities were normalized using the weight of CNTs in the positive electrodes.

S1.4 Simulation of Peeling Process

The simulation of peeling process was implemented using the finite element method. In the simulation, a simplified two-dimensional model was created to simulate the peeling process of hydrogels adhered to rigid substrates [S2, S3]. For the simulated model, the hydrogel matrix was modeled with CPE4RH element, the stiff backing and substrate were modeled with CPE4R element, and the adhered interface between the hydrogel and substrate was modeled with COH2D4 element. For investigating the influence of hydrogel toughness on the peeling process, we used the tough hydrogel with energy dissipation and pure elastic hydrogel without energy dissipation to compare the peeling processes, respectively. We simulated several peeling tests by prescribing different energy values of the interfacial interactions Γ_0, in which S_{max} was fixed to be 500 kPa, and δ_{max} was varied from 0.2 to 1.0 mm. The interfacial toughness as a function of different energy of interfacial interactions was then obtained.

S1.5 Molecular Dynamics (MD) Simulations

The MD simulations were performed on the GROMACS 2021 software package [S4] with OPLSS-AA force field [S5]. The water molecule was modeled with TIP3P element [S6]. In the simulations, the NPT (Number of atoms, pressure, and temperature are constant) ensemble was applied to deduce the system equilibrium. The temperature and pressure in the equilibrium process were maintained at 298.15 K by the V-rescale thermostat and at 1 bar by the Berendsen barostat, respectively. For studying the relationship between ion solvation configurations and ion interactions, we used aqueous solutions with three $ZnCl_2$:LiCl molality ratios (5:0, 5:6, 5:12) to simulate the interactions among ions and water. After 20 ns of MD simulations, the radical distribution function (RDF) g(r) and the coordination number distribution function N(r) of Zn^{2+}–O_W, Zn^{2+}–Cl^-, and Cl^-–H_W in different solutions, as well as the number of H-bonds between water molecules were analyzed.

S2 Supporting Figures and Tables

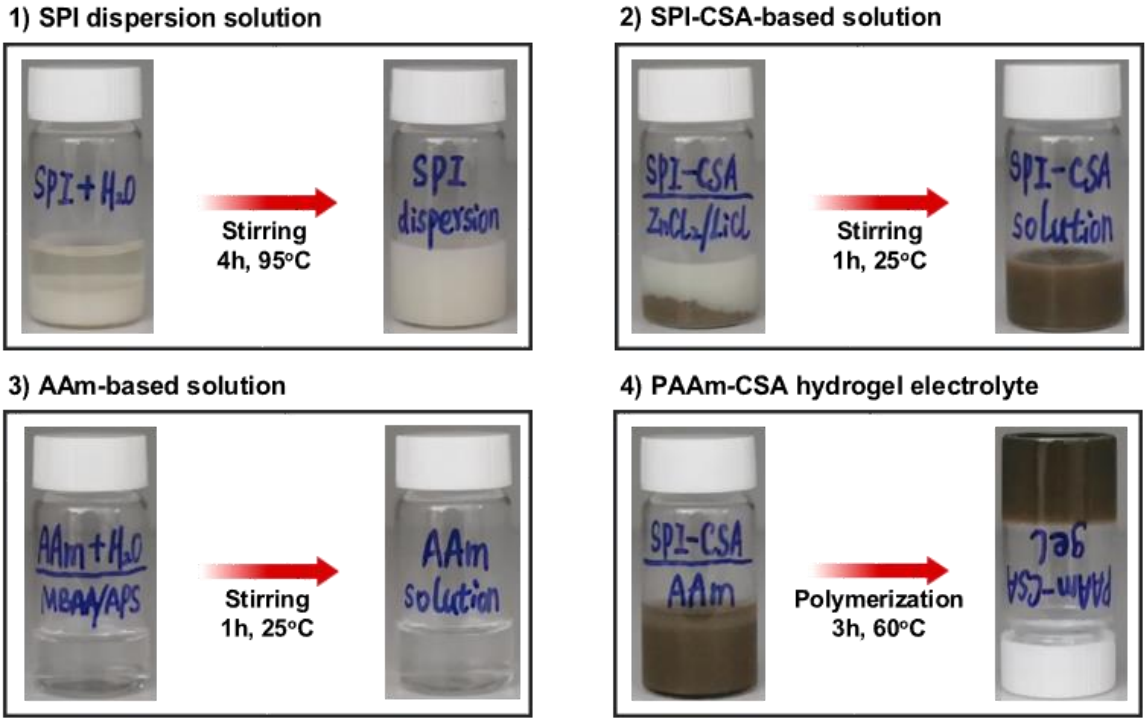

Fig. S1 Schematic procedure for the fabrication of hydrogel electrolyte. The hydrogel electrolyte was fabricated in four steps. First, the SPI dispersion solution was made by dispersing SPI in water at 95 °C for 4 h. Second, the SPI-CSA-based solution was prepared by dissolving CSA, hybrid salts of $ZnCl_2$ and LiCl in SPI dispersion solution at room temperature. Inorganic crystals of hydrated CSA were well dispersed and stabilized in water. Third, AAm-based solution was prepared by dissolving AAm (monomer), MBAA (crosslinker), APS (initiator) in water at room temperature. Finally, the SPI-CSA-based solution and AAm-based solution were homogeneously mixed to be a precursor solution, and then polymerized at 60 °C for 3 h, resulting in the formation of PAAm-CSA hydrogel electrolyte

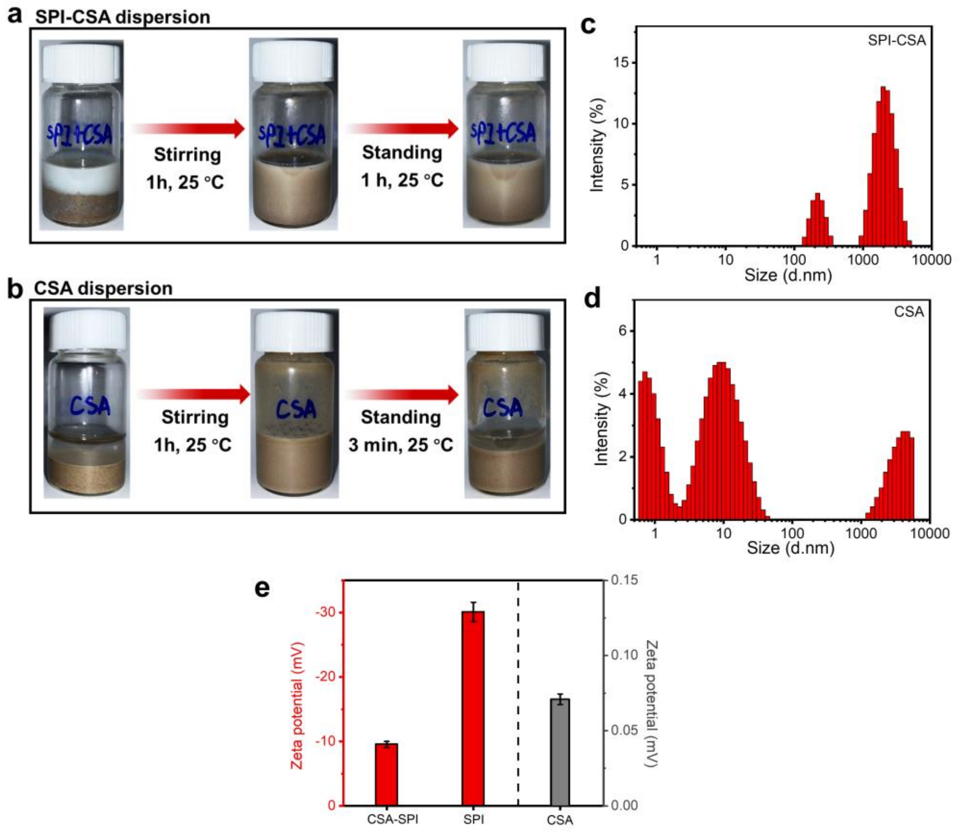

Fig. S2 Stability of SPI-CSA and CSA dispersion. (**a, b**) Optical images of the SPI-CSA and CSA dispersion. (**c, d**) Size distributions of the SPI-CSA and CSA dispersion. (**e**) Zeta potentials of the SPI-CSA, CSA and SPI dispersion

To evaluate the stabilizing effect of SPI nanoparticles on the hydrated CSA crystals, we discuss the stability of SPI-CSA and CSA dispersion, including the apparent homogeneity, size distributions and zeta potentials. As shown in Fig. S2a, b, the SPI-CSA dispersion presents a homogeneous mixture, and remains well-dispersed even after standing for 1 h. In contrast, the CSA dispersion without SPI shows a muddy state, and rapidly precipitates when standing for 3 min. This comparison confirms that the introduction of SPI is beneficial to stabilizing the hydrated CSA crystals in water. Meanwhile, the size distribution of SPI-CSA dispersion shows that hydrated CSA crystals are uniformly dispersed in SPI solution, in a drastic contrast to the behavior of CSA dispersion (Fig. S2c, d). Moreover, zeta potential results indicate that negatively charged SPI nanoparticles are fully absorbed on the surface of positively charged hydrated CSA crystals (Fig. S2e), thus inducing repulsive force between the CSA crystals for stabilization. Therefore, these results reveal that SPI serving as a bio-based pickering dispersant contributes to the stabilization of the hydrated CSA crystals through electrostatic interactions between Ca^{2+}/Al^{3+} of hydrated CSA and $–COO^{-}$ of SPI nanoparticles.

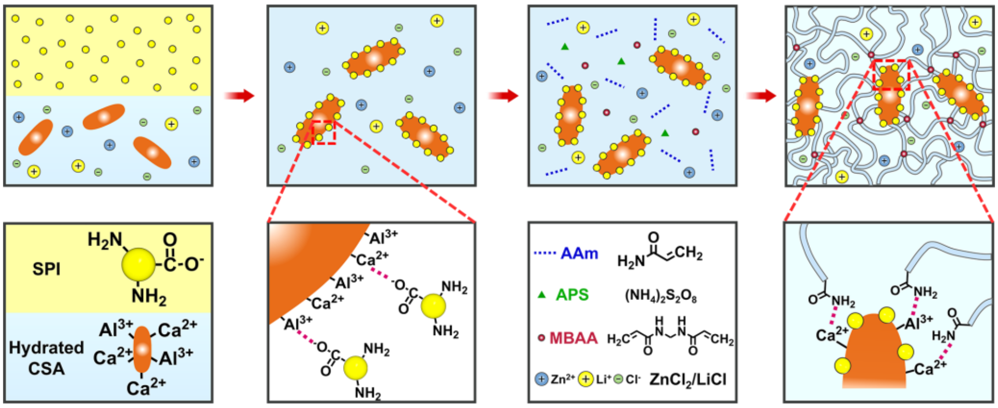

Fig. S3 Polymerization process of the PAAm-CSA hydrogel electrolyte. First, CSA was hydrated with water to generate inorganic crystals of hydrated CSA. Second, inorganic crystals of hydrated CSA absorbed SPI nanoparticles by electrostatic interactions between Ca^{2+}/Al^{3+} of hydrated CSA and $–COO^{-}$ of SPI nanoparticles, thus keeping stabilized in the dispersion. Then, the stable hydrated CSA, AAm monomer, APS initiator, MBAA cross-linker, and $ZnCl_2$/LiCl hybrid salts were mixed together to form a precursor solution. Finally, AAm monomers were polymerized and chemically cross-linked to form PAAm network, followed by coordinating with hydrated CSA, leading to the organic-inorganic composite of hydrogel electrolyte

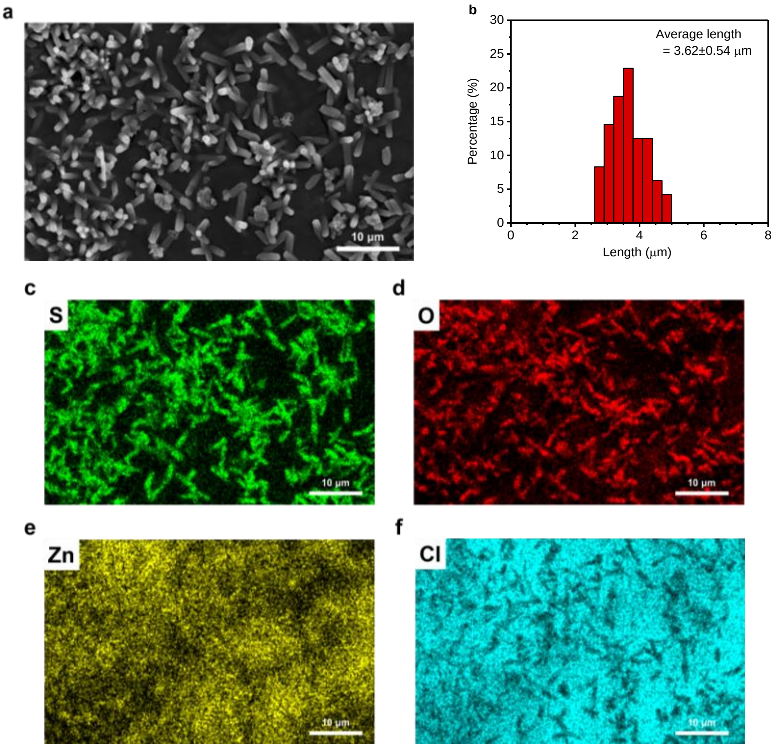

Fig. S4 Morphology of the hydrogel electrolyte. (**a**) SEM image of the hydrogel electrolyte. (**b**) Length distribution of hydrated CSA in (a). The inorganic phase of needle-shaped hydrated CSA with length ~3.6 μm is well dispersed in the continuous phase of PAAm porous polymer network. (**c-f**) Corresponding EDS mapping images of S, O, Zn and Cl elements for the SEM image in (a). S and O elements of the hydrated CSA are incorporated into the polymer skeleton. Zn and Cl elements serving as an ion-conducting phase are uniformly distributed in the porous channel of the polymer network

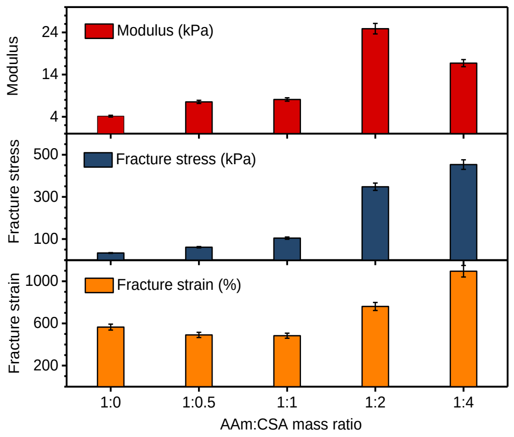

Fig. S5 Summary of Young's modulus, fracture stress and fracture strain of hydrogel electrolytes with different AAm:CSA mass ratios. Values correspond to the tensile stress versus strain curves in Fig. 2a

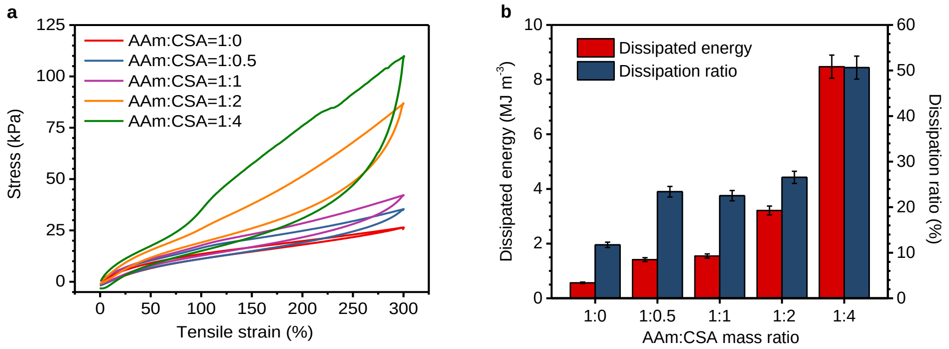

Fig. S6 Tension-release behavior of hydrogel electrolytes with different AAm:CSA mass ratios. (**a**) Tension-release hysteresis loops of various hydrogel electrolytes at 300% tensile strain. (**b**) Values of dissipated energy and dissipation ratio corresponding to tension-release curves in (a)

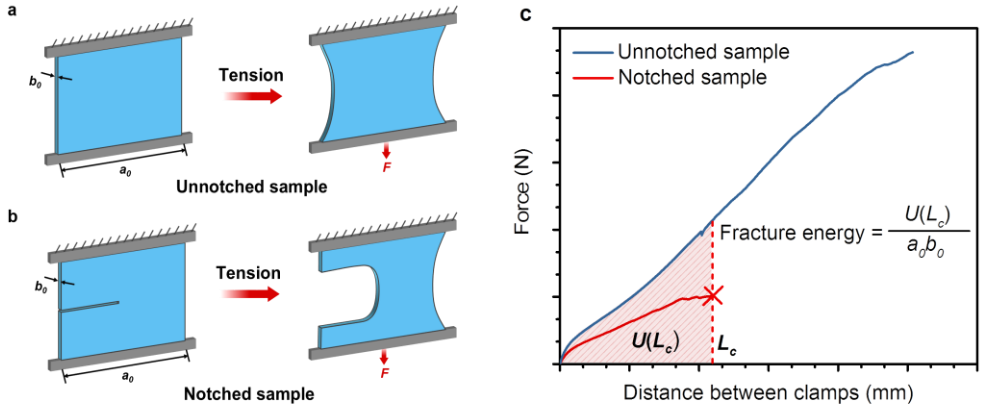

Fig. S7 Pure-shear test for measuring fracture energy of hydrogel electrolytes. (**a, b**) Schematics of the pure-shear test for the (**a**) unnotched sample and (**b**) notched sample. (**c**) Force versus distance curves for the unnotched (blue line) and notched (red line) hydrogel electrolytes for fracture energy determination. The light red area is the work $U(L_c)$ done by the tensile force to the unnotched sample at L_c. L_c indicates the critical distance between clamps at which the notched sample starts to propagate the crack.

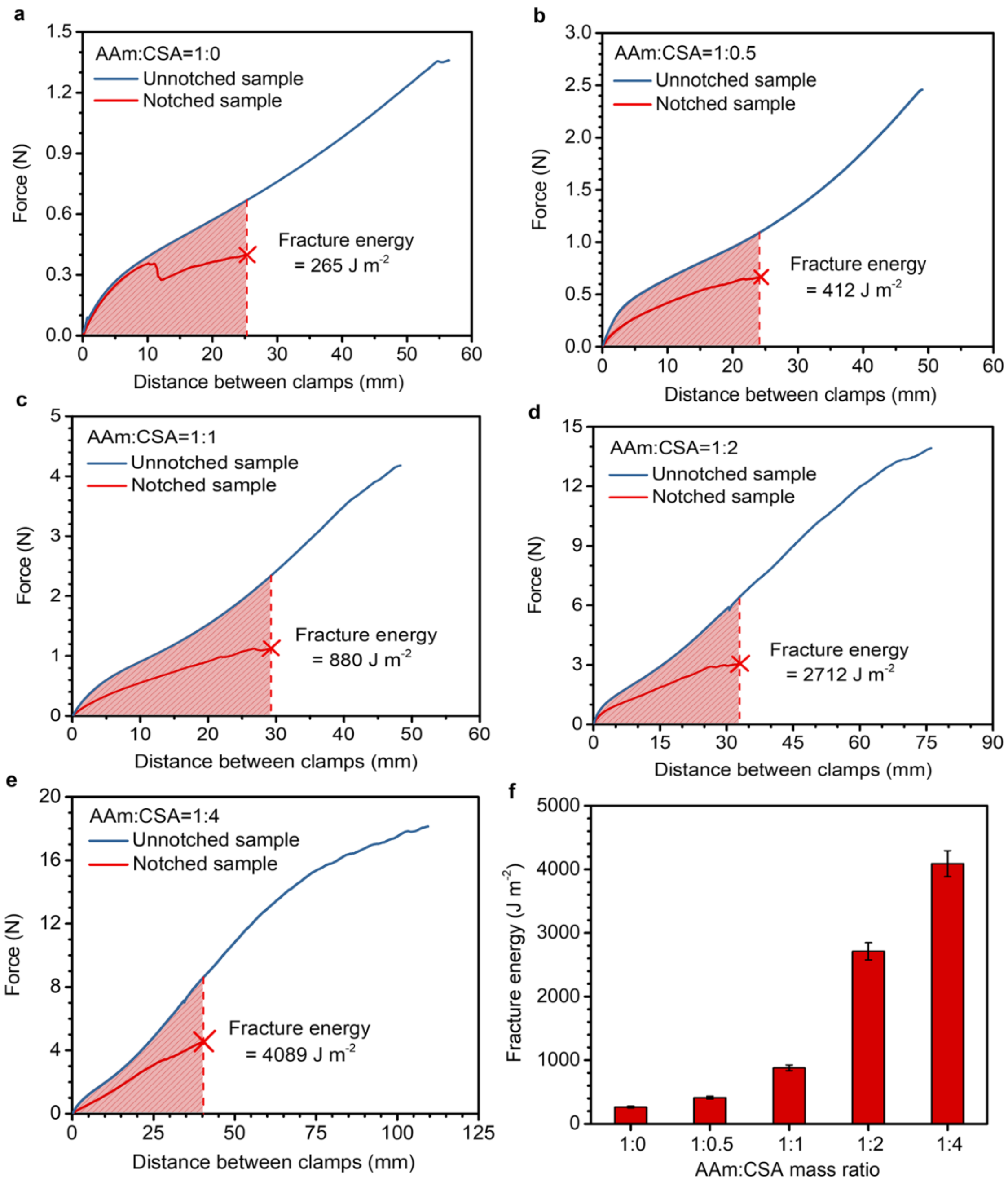

Fig. S8 Fracture energy of hydrogel electrolytes with different AAm:CSA mass ratios. (**a-e**) Force versus distance curves of various hydrogel electrolytes for the fracture energy determination, in which the AAm:CSA mass ratios ranges from 1:0 to 1:4. (**f**) Comparison of fracture energy for various hydrogel electrolytes

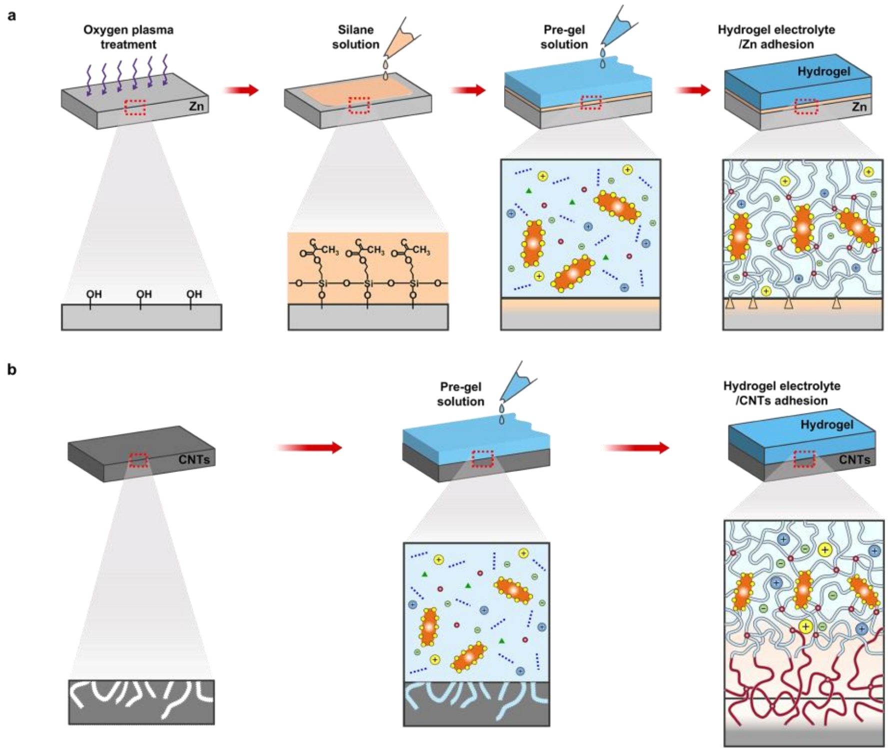

Fig. S9 Schematic process of hydrogel electrolyte/electrode adhesion. (**a**) In situ adhesion of hydrogel electrolyte on Zn electrode. The adhesion of hydrogel electrolyte/Zn electrode was obtained by in situ copolymerizing the precursor solution on the silanized Zn surface. (**b**) In situ adhesion of hydrogel electrolyte on CNTs electrode. The adhesion of hydrogel electrolyte/CNTs electrode was acquired by penetrating AAm-based solution into the porous CNTs surface and covalently cross-linking.

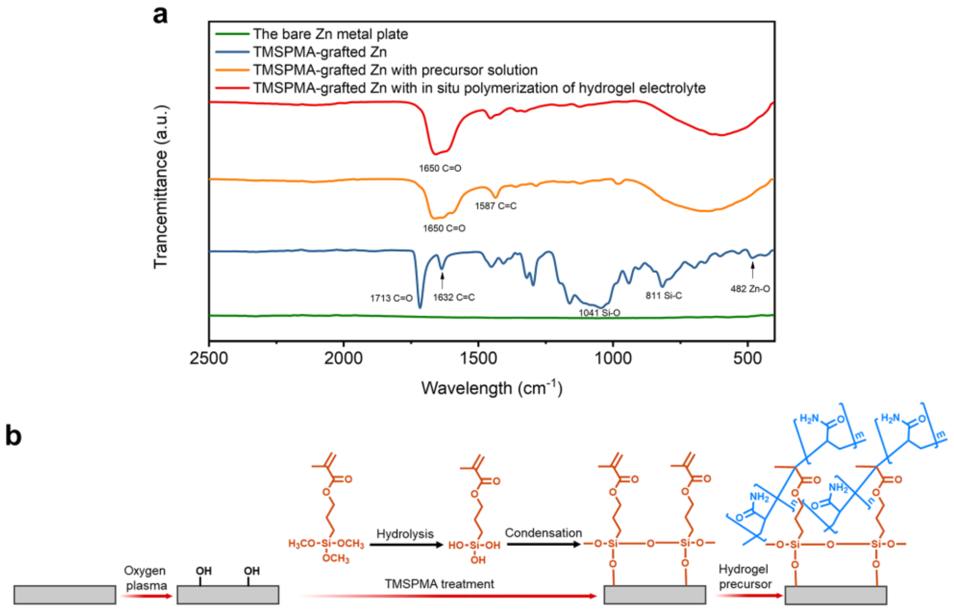

Fig. S10 Mechanism of interactions between the TMSPMA-grafted Zn electrode and the hydrogel electrolyte. (**a**) FTIR spectra of the Zn metal surface before and after interaction. (**b**) The proposed mechanism of interactions between the TMSPMA-grafted Zn electrode and the hydrogel electrolyte

To analyze the mechanism of interactions between the TMSPMA-grafted Zn electrode and the hydrogel electrolyte, we used Fourier transform infrared spectroscope (FTIR) to track the chemical compositions of the Zn metal surface before and after interaction.

As shown in Fig. S10a, compared with the bare Zn metal, the TMSPMA-grafted Zn shows several characteristic peaks, including 1713 cm^{-1} (sharp) for C=O, 1632 cm^{-1} for C=C, 1041 cm^{-1}(broad) for Si–O, 811 cm^{-1} for Si–C, and 482 cm^{-1} for Zn–O. This result suggests that the TMSPMA is successfully grafted onto the Zn surface. We then compared FTIR spectra of the TMSPMA-grafted Zn before and after in situ copolymerization of hydrogel electrolyte. The TMSPMA-grafted Zn with precursor solution shows peaks at 1650 cm^{-1} and 1587 cm^{-1}, corresponding to C=O and C=C of AAm molecules. For comparison, the TMSPMA-grafted Zn with in situ polymerization of hydrogel electrolyte exhibits a decreased peak at 1587 cm^{-1}, which indicates that the methacrylate groups of grafted TMSPMA can copolymerize with the acrylate groups of AAm monomers through free radical polymerization, generating chemically anchored polymer network onto Zn surface.

The proposed mechanism of interactions between the TMSPMA-grafted Zn electrode and the hydrogel electrolyte is schematically illustrated in Fig. S10b. First, Zn metal plate is treated with oxygen plasma to add hydroxyl groups on its surface. Second, upon dissolving in water, the methoxy groups of TMSPMA hydrolyzes to form silanol groups. These silanol groups then condensate with the hydroxyl groups on Zn surface, thus forming the TMSPMA-grafted Zn metal surface. Finally, when the precursor solution was poured onto the TMSPMA-grafted Zn surface, the methacrylate groups of grafted TMSPMA can copolymerize with the acrylate groups of AAm monomers, thus generating the chemically anchored polymer network in situ.

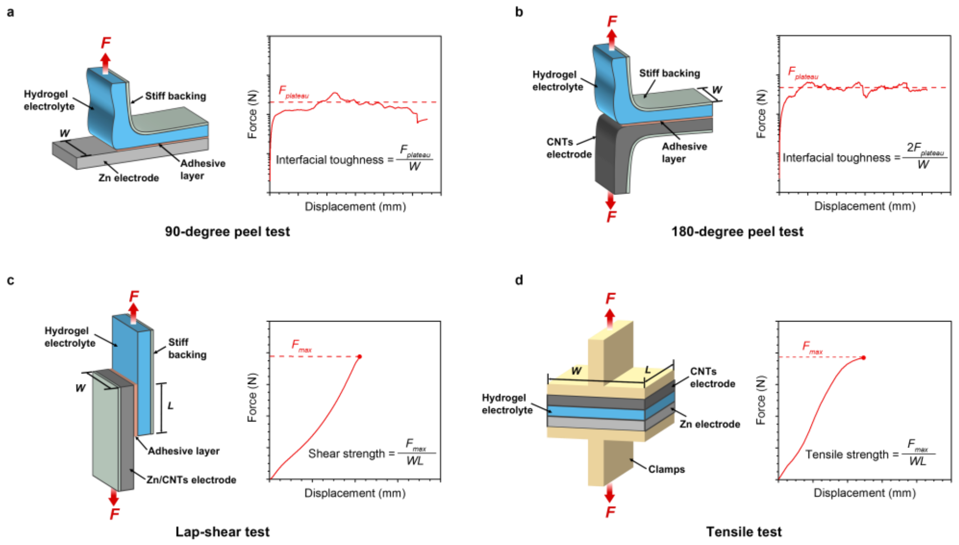

Fig. S11 Measurement of adhesion performance. (a) 90-degree peel test (ASTM D2861) for the interfacial toughness of hydrogel electrolyte/Zn hybrid. The interfacial toughness is determined by dividing the plateau force ($F_{plateau}$) by the adhesion width (W). (b) 180-degree peel test (ASTM F2256) for the interfacial toughness of hydrogel electrolyte/CNTs hybrid. The interfacial toughness is determined by dividing two times the plateau force ($F_{plateau}$) by the adhesion width (W). (c) Lap-shear test (ASTM F2255) for the shear strength of hydrogel electrolyte/Zn and hydrogel electrolyte/CNTs hybrids. The shear strength is determined by dividing the maximum force (F_{max}) by the adhesion

area (*WL*). (d) Tensile test (ASTM F2258) for the tensile strength of Zn/hydrogel electrolyte/CNTs hybrid. The tensile strength is determined by dividing the maximum force (F_{max}) by the adhesion area (*WL*).

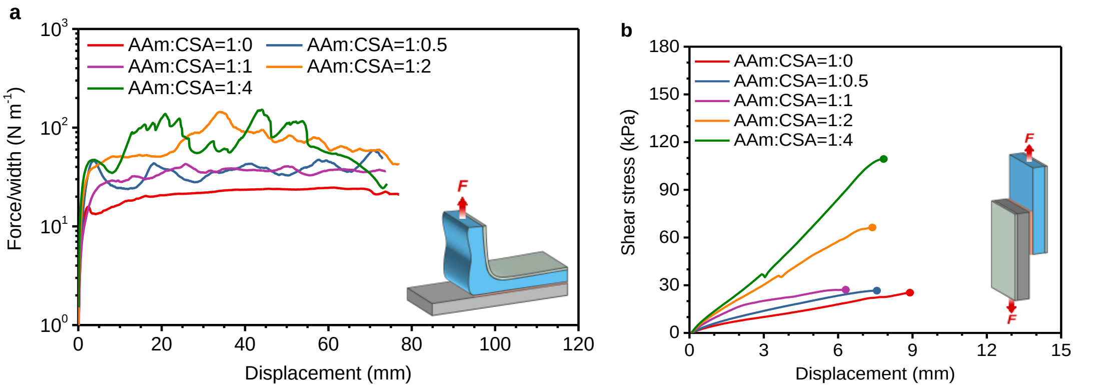

Fig. S12 Adhesion test of hydrogel electrolyte/Zn hybrids with different AAm:CSA mass ratios. (**a**) Force/width versus displacement curves for 90-degree peel tests of various hydrogel electrolyte/Zn hybrids. (**b**) Shear stress versus displacement curves for lap-shear tests of various hydrogel electrolyte/Zn hybrids

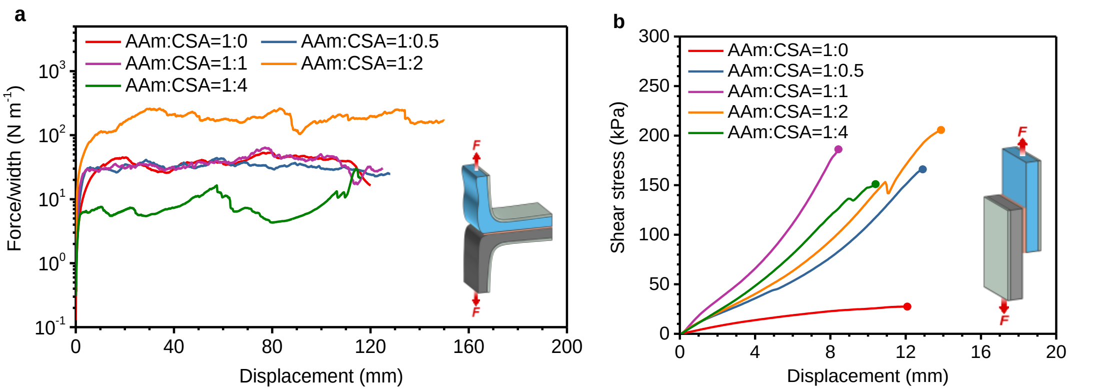

Fig. S13 Adhesion test of hydrogel electrolyte/CNTs hybrids with different AAm:CSA mass ratios. (**a**) Force/width versus displacement curves for 180-degree peel tests of various hydrogel electrolyte/CNTs hybrids. (**b**) Shear stress versus displacement curves for lap-shear tests of various hydrogel electrolyte/CNTs hybrids

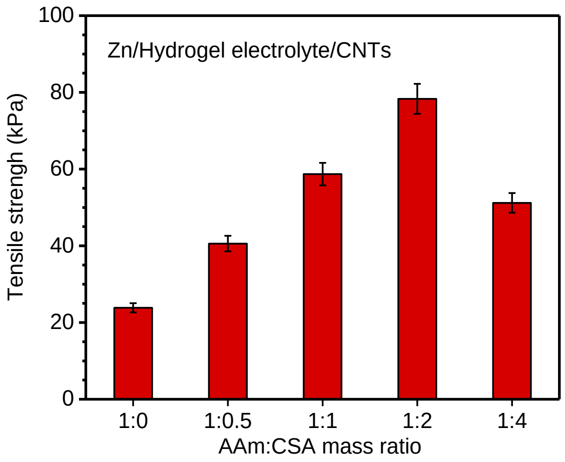

Fig. S14 Comparison of tensile strength for various Zn/hydrogel electrolyte/CNTs hybrids. Values correspond to tensile stress versus displacement curves in Fig. 2c

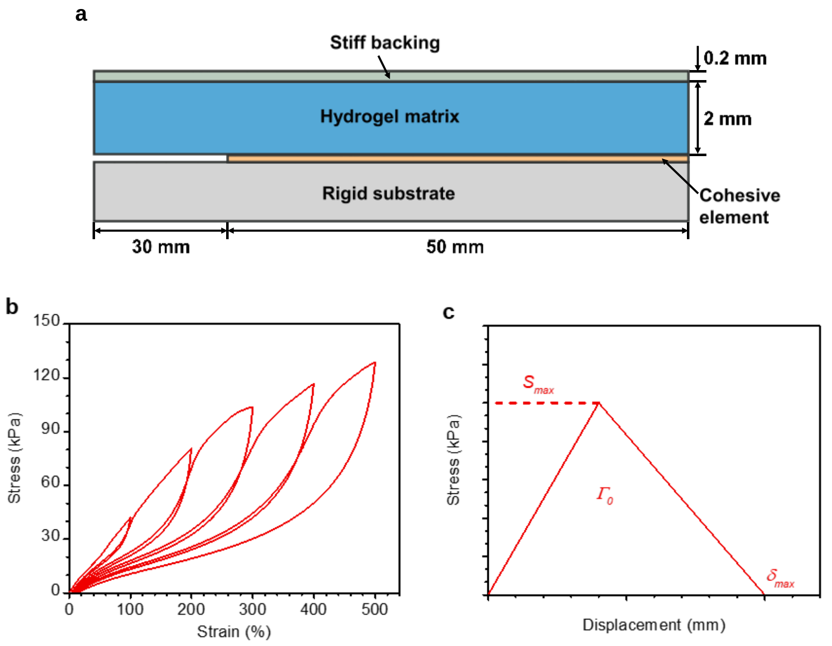

Fig. S15 Peeling simulation of hydrogels from rigid substrates. (**a**) Schematic of the finite element model. A hydrogel with rectangle shape (of length 80 mm and thickness 2 mm) was adhered on a rigid substrate, where a partial hydrogel (of length 30 mm) was initially detached. The model was simplified as a plane-strain deformation. The elastic property and energy dissipation of the hydrogels were characterized with the Ogden hyperelastic material and Mullins effect, respectively. The stiff backing was modeled as a linear elastic material with Young's modulus of 1.5 GPa. The adhered interface between the hydrogel and substrate was simulated as the cohesive element. (**b**) Experimental data for fitting the tough hydrogel model. (**c**) Triangular cohesive law for the cohesive element. The parameters of the cohesive element were fixed as maximum strength S_{max} = 500 kPa and maximum separation distance δ_{max} = 1 mm. The cohesive element followed the Quads Damage

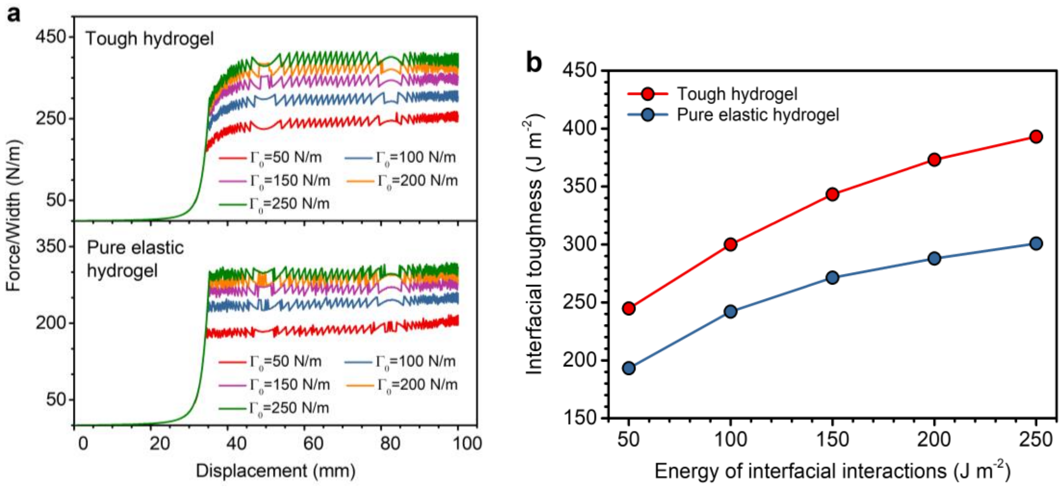

Fig. S16 Simulated peeling results of various hydrogels from rigid substrates by finite element analysis. (**a**) Simulated force/width versus displacement curves of the tough hydrogel (top) and the pure elastic hydrogel (bottom) from rigid substrates. Γ_0 represents the energy of interfacial interactions. (**b**) Calculated interfacial toughness as a function of the energy of interfacial interactions for the tough hydrogel (red) and the pure elastic hydrogel (blue)

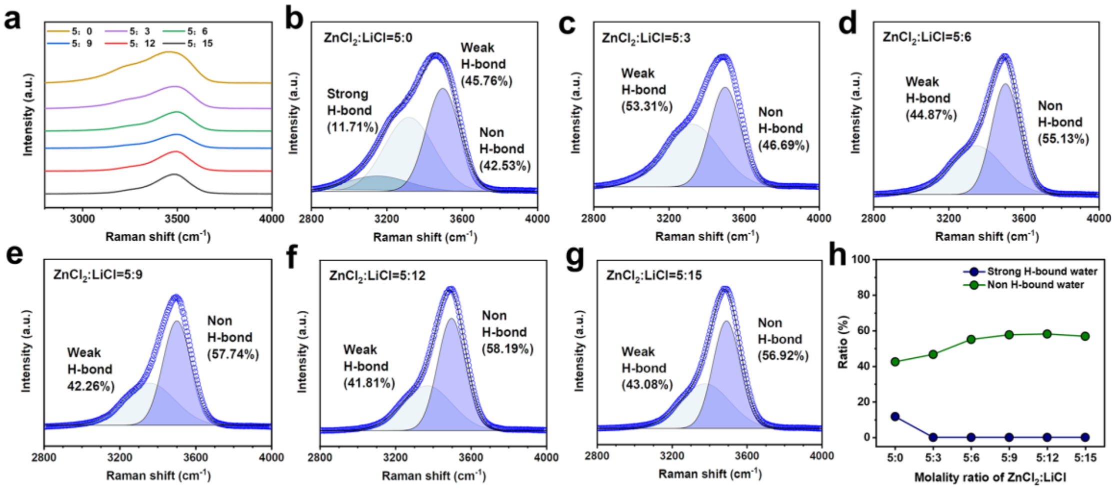

Fig. S17 Raman spectra of hydrogel electrolytes with different $ZnCl_2$:LiCl molality ratios. (**a**) O–H stretching vibrations of hydrogel electrolytes with different $ZnCl_2$:LiCl molality ratios from 5:0 to 5:15. (**b-g**) The corresponding fitted O–H stretching vibrations representing water molecules with strong, weak and non H-bonds. (**h**) The proportion of strong H-bound water and non H-bound water in hydrogel electrolytes with different $ZnCl_2$:LiCl molality ratios.

We conducted Raman spectroscopy tests of hydrogel electrolytes with different molality ratios of $ZnCl_2$:LiCl. As shown in Fig. S17a, when the molality ratio of $ZnCl_2$:LiCl increases from 5:0 to 5:15, the Raman peak in the region of 3000~3700 cm^{-1} gradually shifts to high frequency, suggesting that the water molecules with strong H-bonds are decreasing constantly in the hydrogel electrolyte [S7, S8]

We then fitted the Raman spectra and calculated the water ratios with different H-bound states (including strong H-bound water, weak H-bound water and Non-bound water) based on the area of fitted peaks (Fig. S17b-h). When the $ZnCl_2$:LiCl molality ratio increases from 5:0 to 5:12, the strong H-bound water reduces rapidly but non H-bound water increases obviously; As the $ZnCl_2$:LiCl molality ratio further increases to 5:15, the reduction in strong H-bound water and increase in non H-bound water remain stable. This result reveals that the moderate addition of LiCl into the hydrogel electrolyte is beneficial to breaking the H-bonds among water molecules, thus efficiently suppressing ice nucleation and hindering water freezing.

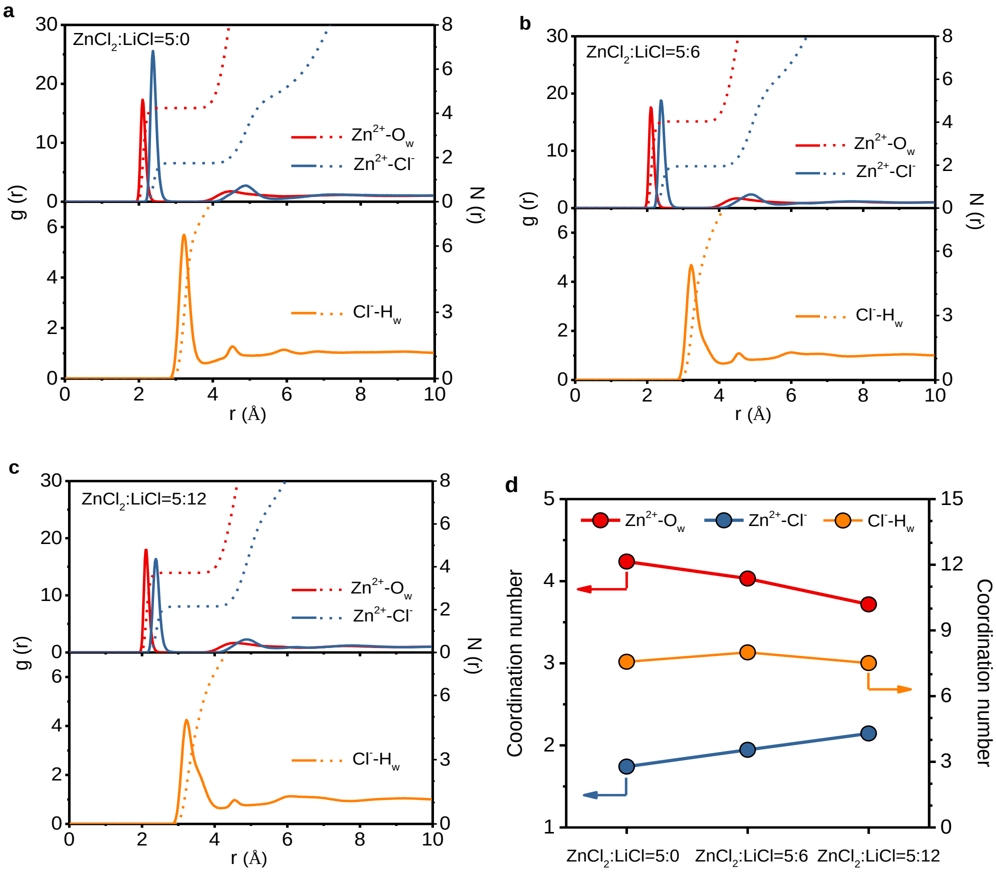

Fig. S18. MD simulations of aqueous solutions with three $ZnCl_2$:LiCl molality ratios. The radical distribution function (RDF) *g(r)* and the coordination number distribution function *N(r)* of Zn^{2+}–O_W, Zn^{2+}–Cl^-, and Cl^-–H_W in aqueous solutions with different $ZnCl_2$:LiCl molality ratios at (**a**) 5:0, (**b**)5:6, and (**c**)5:12. (**d**) Corresponding changes in coordination numbers of Zn^{2+}–O_W, Zn^{2+}–Cl^-, and Cl^-–H_W

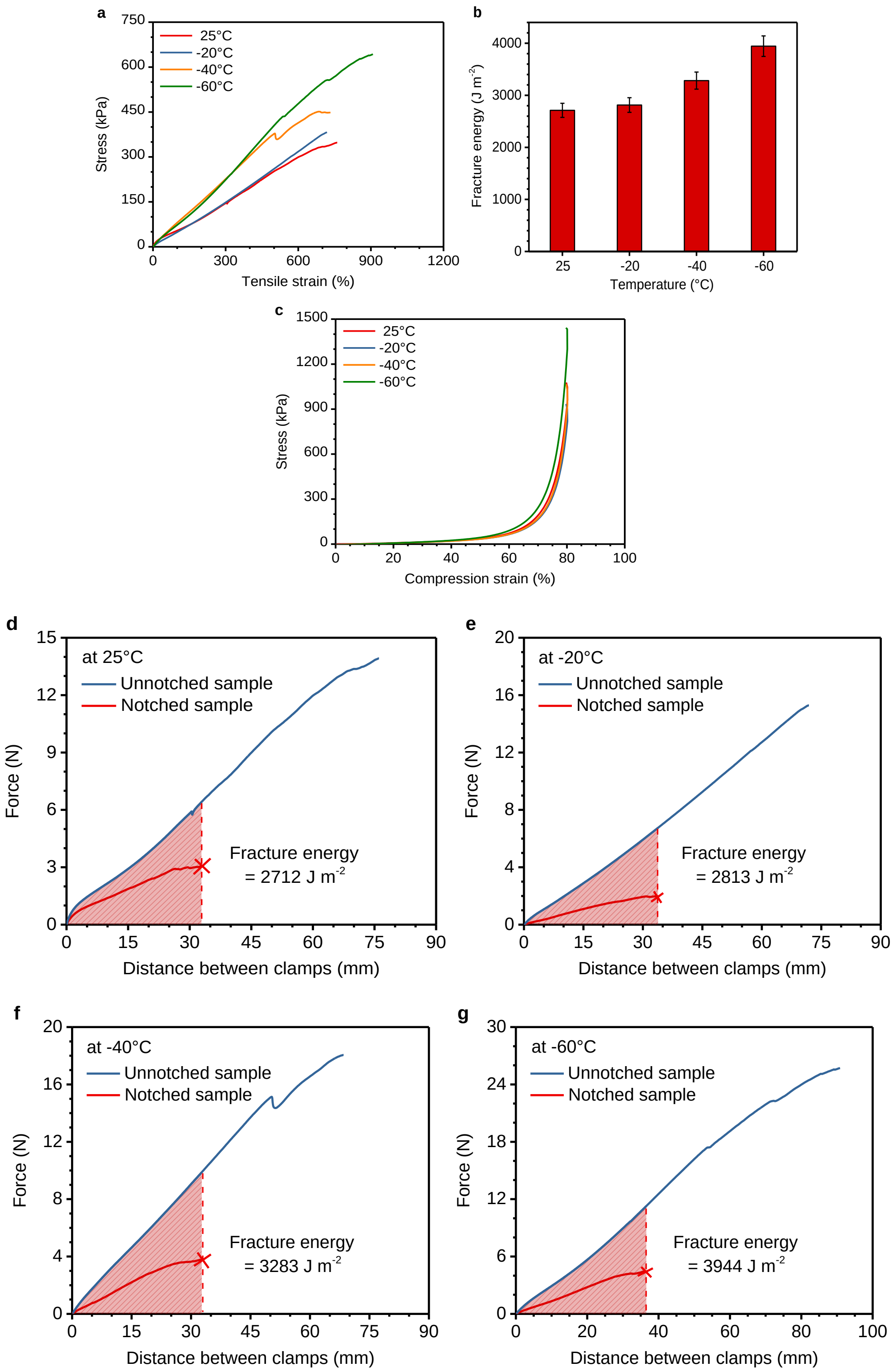

Fig. S19. Low-temperature mechanical property of the hydrogel electrolyte. (**a**) Tensile stress versus strain curves of the hydrogel electrolyte in the temperature range of 25~–60 °C. (**b**) Change of fracture energy for the hydrogel electrolyte as the temperature dropping from 25 °C to −60 °C. (**c**) Compressive stress versus strain curves of the hydrogel electrolyte in the temperature range of 25~–60 °C. Force versus distance curves of the hydrogel electrolyte for fracture energy determination at (**d**) 25, (**e**) –20, (**f**) –40, and (**g**) –60 °C

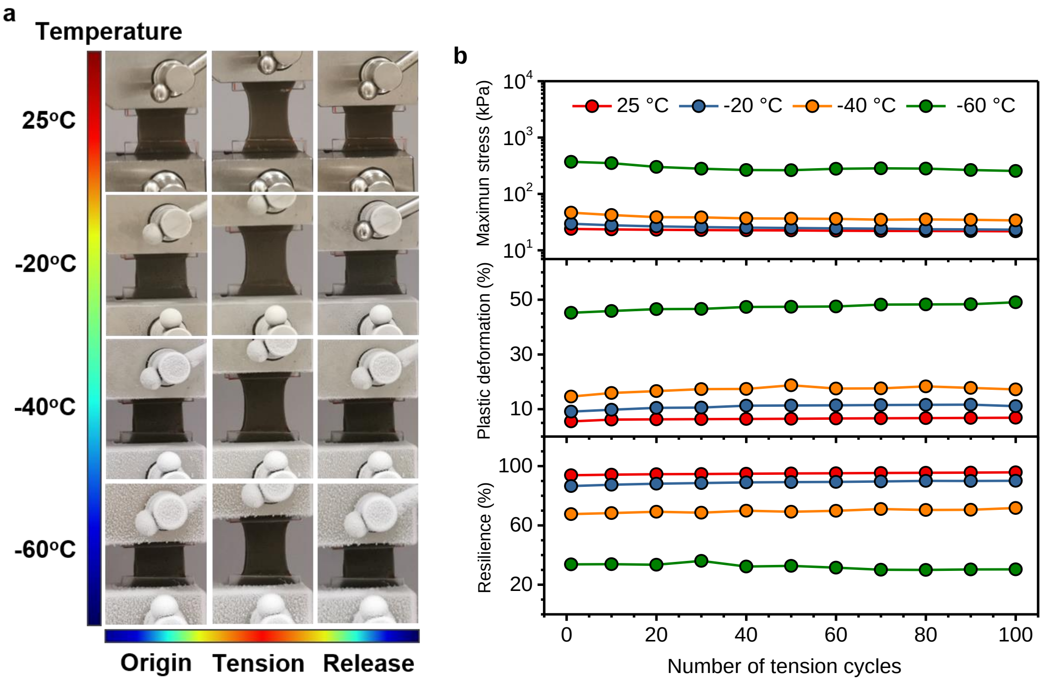

Fig. S20 Cyclic tension behavior of the hydrogel electrolyte at low temperatures. (**a**) Optical images of the hydrogel electrolyte with a $ZnCl_2$:LiCl ratio of 5:12 during tension cycles in the temperature range of 25~–60 °C. (**b**) Changes in maximum stress, plastic deformation, and resilience of the hydrogel electrolyte during 100 tension cycles with 100% strain at 25, −20, −40, and −60 °C

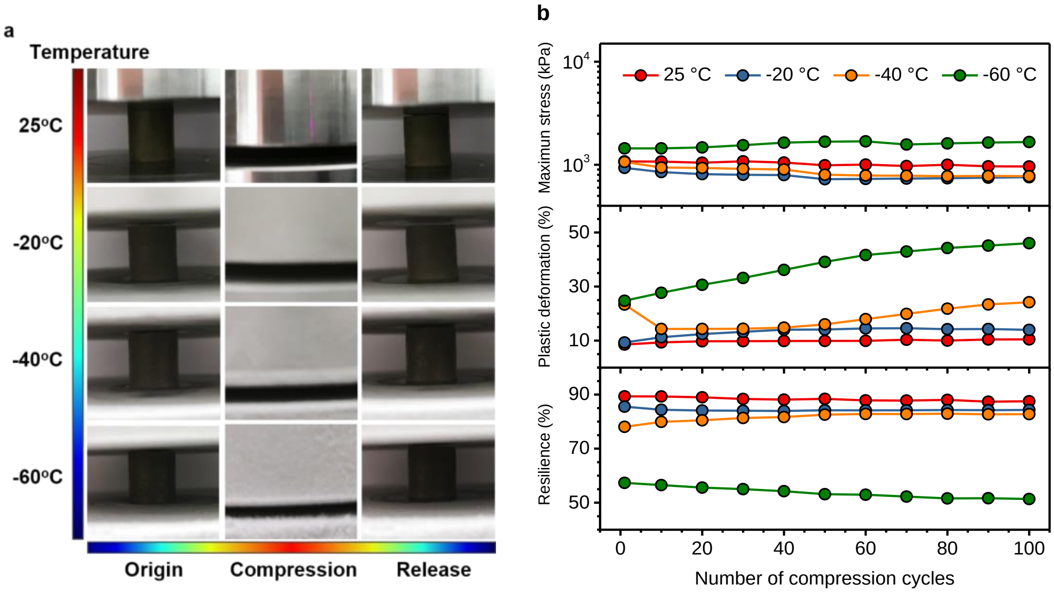

Fig. S21 Cyclic compression behavior of the hydrogel electrolyte at low temperatures. (**a**) Optical images of the hydrogel electrolyte with a $ZnCl_2$:LiCl ratio of 5:12 during compression cycles in the temperature range of 25~–60 °C. (**b**) Changes in maximum stress, plastic deformation, and resilience of the hydrogel electrolyte during 100 compression cycles with 80% strain at 25, −20, −40, and −60 °C

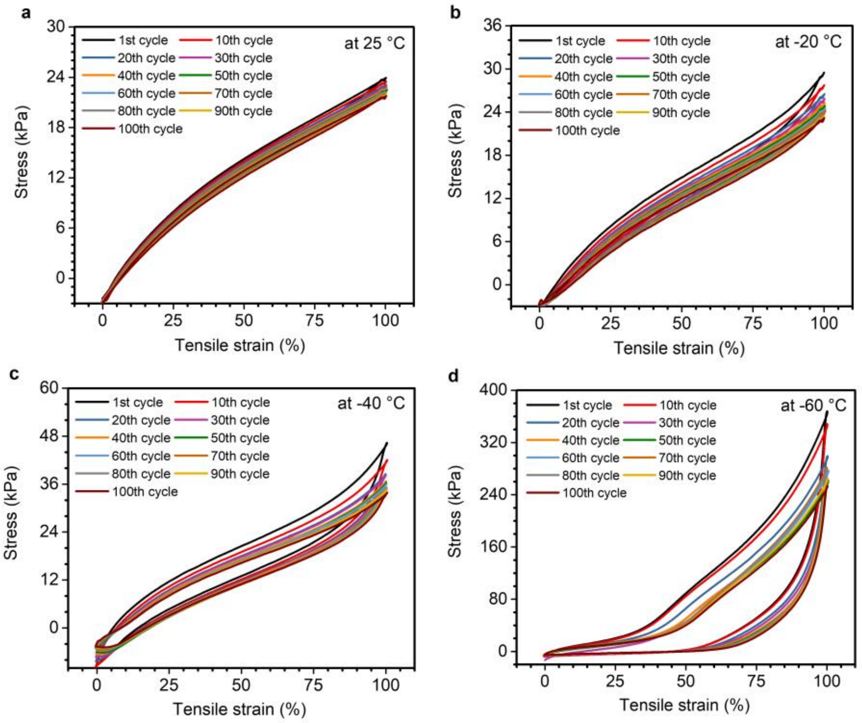

Fig. S22 Tension cycle tests of the hydrogel electrolyte in the temperature range of 25~–60 °C. Tensile stress versus strain curves of the hydrogel electrolyte with a $ZnCl_2$:LiCl ratio of 5:12 under 100% tensile strain for 100 cycles at (**a**) 25, (**b**) –20, (**c**) –40, and (**d**) –60 °C

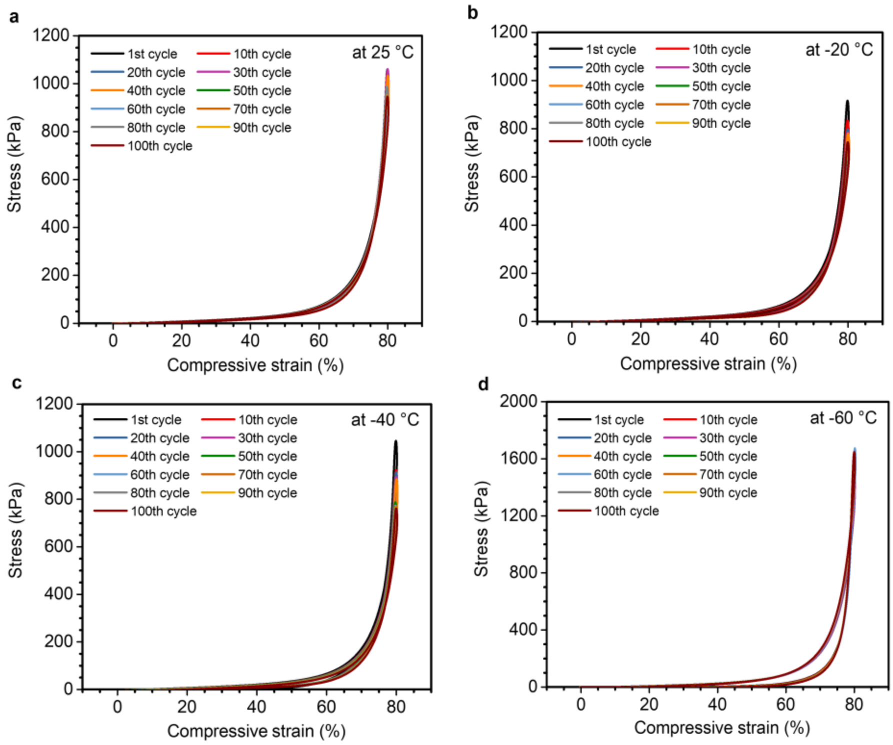

Fig. S23 Compression cycle tests of the hydrogel electrolyte in the temperature range of 25~–60 °C. Compressive stress versus strain curves of the hydrogel electrolyte with a $ZnCl_2$:LiCl ratio of 5:12 under 80% compressive strain for 100 cycles at (**a**) 25, (**b**) –20, (**c**) –40, and (**d**) –60 °C

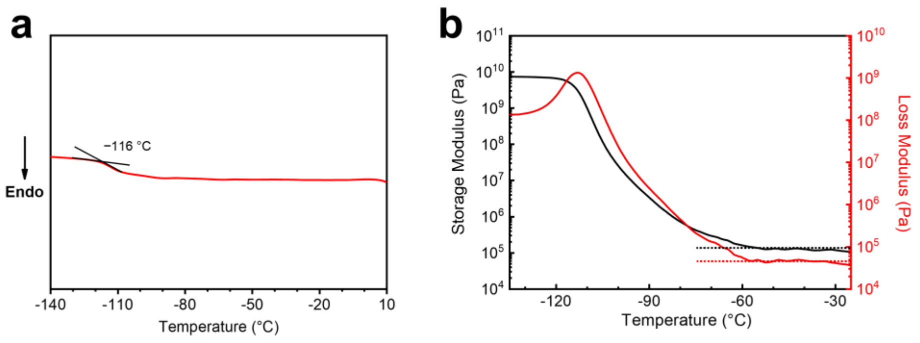

Fig. S24 Differential scanning calorimetry (DSC) and dynamic mechanical analysis (DMA) tests of the hydrogel electrolyte. (**a**) The DSC curve of the hydrogel electrolyte from –140 to 25 °C. The glass transition temperature is about –116 °C. (**b**) The storage modulus (E’) and loss modulus (E’’) of the hydrogel electrolyte from –140 to 20 °C

We conducted differential scanning calorimetry (DSC) and dynamic mechanical analysis (DMA) tests of the hydrogel electrolyte with a critical $ZnCl_2$:LiCl ratio of 5:12. The DSC curve in Fig. S24a shows that the hydrogel electrolyte has a glass transition temperature (T_g) of –116 °C, indicating that the hydrogel electrolyte can endure the lowest temperature of –116 °C. For DMA curves in Fig. S24b, when the temperature increases from –120 °C to –60 °C, the storage modulus (E’) and loss modulus (E’’) of the hydrogel electrolyte decrease abruptly, suggesting that the hydrogel electrolyte experiences a phase transition from frozen state to unfrozen state. Meanwhile, the E’ maintains constant (~10^5 Pa) above the temperature of ~–60 °C, revealing that the hydrogel electrolyte presents mechanical stability and elasticity in the temperature range of 25~–60 °C. These results are consistent with low-temperature mechanical tests results of the hydrogel electrolyte. Therefore, the thermodynamics analysis strongly confirms that the hydrogel electrolyte shows a phase transition around –116 °C, and is able to maintain mechanical stability above −60 °C.

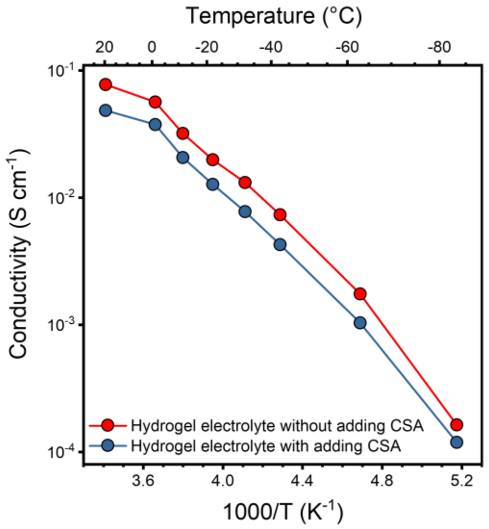

Fig. S25 Ionic conductivities of hydrogel electrolytes with and without CSA in the temperature range of 25~–80 °C. To investigate the effect of hydrated CSA crystals on ions transport in the hydrogel electrolyte, we compared the ionic conductivities between the hydrogel electrolytes with and without CSA in the temperature range of

25~–80 °C. Clearly, the introduction of CSA into the hydrogel electrolytes results in a slight decrease in the ionic conductivity. One possible explanation is that the addition of CSA could partially impedes the ions transport in the hydrogel electrolyte, due to the fact that the inorganic phase of hydrated CSA crystals is filled in the continuous phase of PAAm porous polymer network, thus reducing the porous channels for ions transport.

However, we notice that changes in the ionic conductivities of the hydrogel electrolytes with and without CSA exhibit a similar trend as the temperature drops from 25 to –80 °C, indicating that the addition of CSA has no negative effect on ions transport at low temperatures. Considering that the incorporation of hydrated CSA nanocrystals plays a vital role in stiffening and strengthening the polymer skeleton at low temperatures, it is necessary to adding moderate content of CSA in the hydrogel electrolyte.

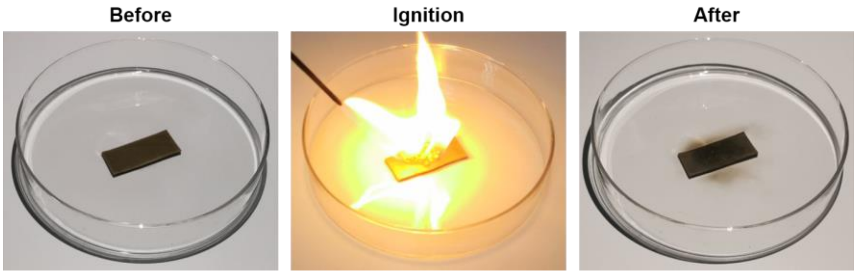

Fig. S26 Flame retardancy of the hydrogel electrolyte. Optical images of flammability test for the hydrogel electrolyte

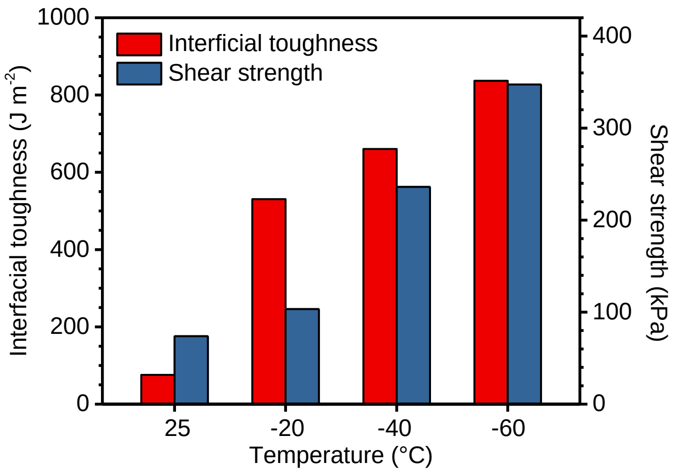

Fig. S27 Low-temperature adhesion performance of the Zn/hydrogel electrolyte/CNTs. Changes of interfacial toughness and shear strength for the Zn/hydrogel electrolyte/CNTs with the temperature dropping from 25 °C to –60 °C

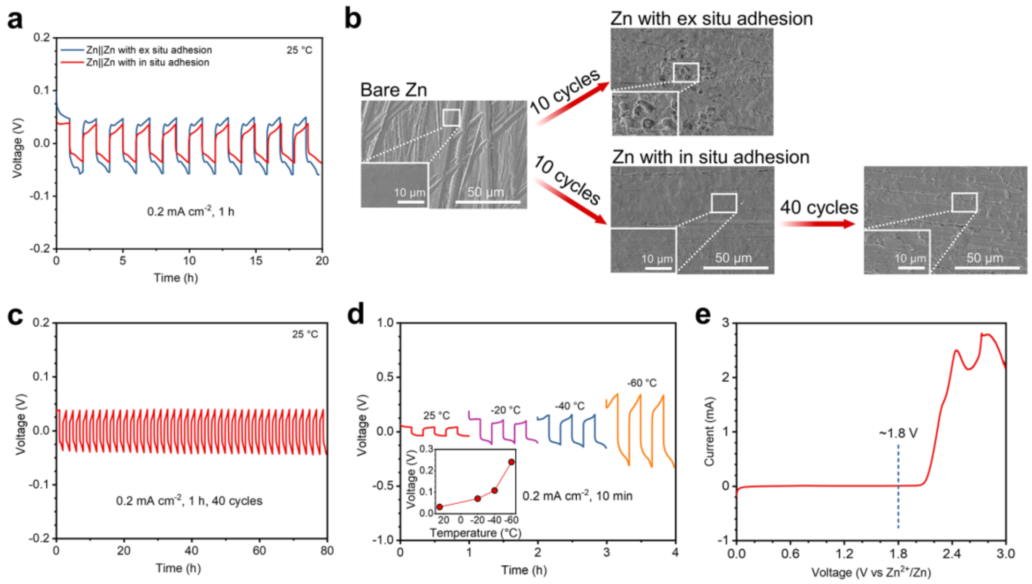

Fig. S28 Voltage profiles of the symmetric Zn||Zn cells. (**a**) Zn plating/stripping tests of the Zn||Zn cells assembled with different ways. (**b**) SEM images of the Zn anodes surface assembled with ex situ adhesion and in situ adhesion. (**c**) Cycling performance of the Zn||Zn cell with in situ adhesion at 25 °C. (**d**) Zn plating/stripping test of the Zn||Zn cell with in situ adhesion at 25~–60 °C. The inset is the corresponding overpotential at each temperature. (**e**) Linear sweep voltammetry (LSV) curve of the hydrogel electrolyte in Zn||Pt cell at a scan rate of 1 mV s^{-1}

Compared with the Zn||Zn cell with ex situ adhesion, the Zn||Zn cell with in situ adhesion shows obviously lower overpotential (Fig. S28a), which indicates that the robust interfacial contact formed by in situ adhesion of hydrogel electrolyte can effectively reduce the interfacial resistance and generate a uniform Zn-ion flux. Meanwhile, the morphologies of the Zn metal anodes with ex situ adhesion and in situ adhesion were compared after 10 plating/stripping cycles at 0.2 mA cm^{-2}. As seen in Fig. S28b, the Zn anode with ex situ adhesion shows protruding dendrites on the surface. In contrast, the Zn anode with in situ adhesion exhibits a much smoother and flatter surface. This result confirms that the well-adhered interface formed by in situ adhesion is more beneficial to the stable Zn plating and stripping. Also, the symmetric Zn cell with in situ adhesion demonstrates excellent stability and cyclability, maintaining a low overpotential of below 26 mV over 40 plating/stripping cycles (Fig. S28c). At the same time, the cycled Zn anode still keeps smooth on the surface, showing a dendrite-free morphology after 40 cycles (Fig. S28b).

Furthermore, we tested the symmetric Zn cell with in situ adhesion in the temperature of 25~−60 °C (Fig. S28d). When the temperature drops from 25 °C to −60 °C, even though the symmetric cell shows increased overpotential due to the reduced ionic conductivity and sluggish Zn plating/stripping kinetics at lower temperatures, it remains working even at −60 °C.

In addition, the electrochemical stability window of the hydrogel electrolyte was measured by linear sweep voltammetry (LSV) in Zn||Pt cell, showing an oxidation wave over 1.8 V (Fig. S28e). This enhancement of oxidative stability is attributed to the cooperative solvation of $ZnCl_2$ and LiCl, resulting in the scarcity of free water molecules and suppression of water decomposition.

These results suggest that the hydrogel electrolyte with strong adhesion and high anti-freezing properties favors Zn reversibility and compatibility, enabling stable Zn plating/stripping cycles across a temperature range of 25 °C to −60 °C.

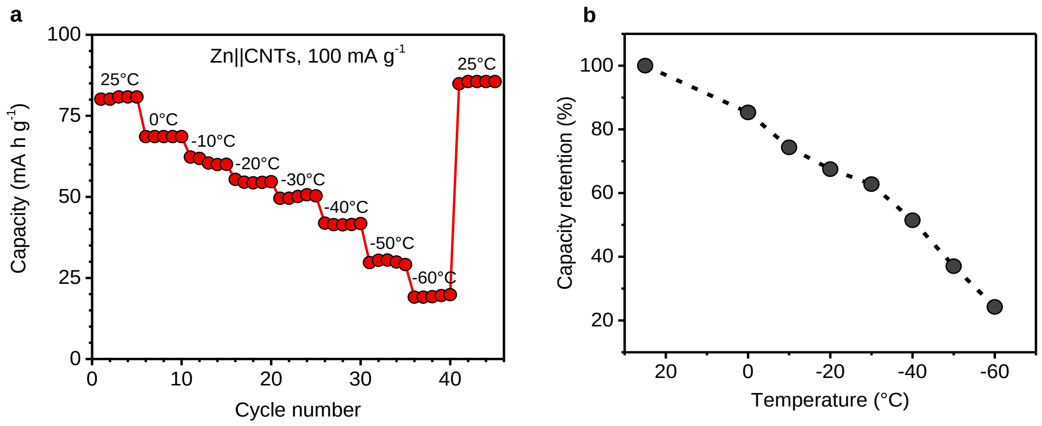

Fig. S29 Capacity changes of the Zn||CNTs hybrid capacitor in the temperature range of 25~–60 °C. (**a**) Discharge capacities of the hybrid capacitor with the current density of 0.1 A g^{-1} at temperatures dropping from 25 to –60 °C. (**b**) The corresponding capacity retention at different temperatures

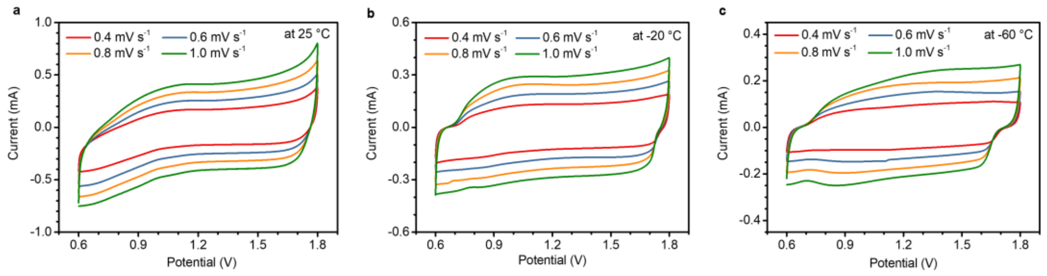

Fig. S30 CV tests of the Zn|hydrogel electrolyte|CNTs hybrid capacitor at different temperatures. CV curves of the Zn||CNTs hybrid capacitor in the scan rates of 0.4~1.0 mV s^{-1} at (**a**) 25, (**b**) –20, and (**c**) –60 °C. The CV results show quasi-rectangular shapes in the temperature range of 25~–60 °C, indicating the stable and reversible capacitive performance of the hybrid capacitor at low temperatures

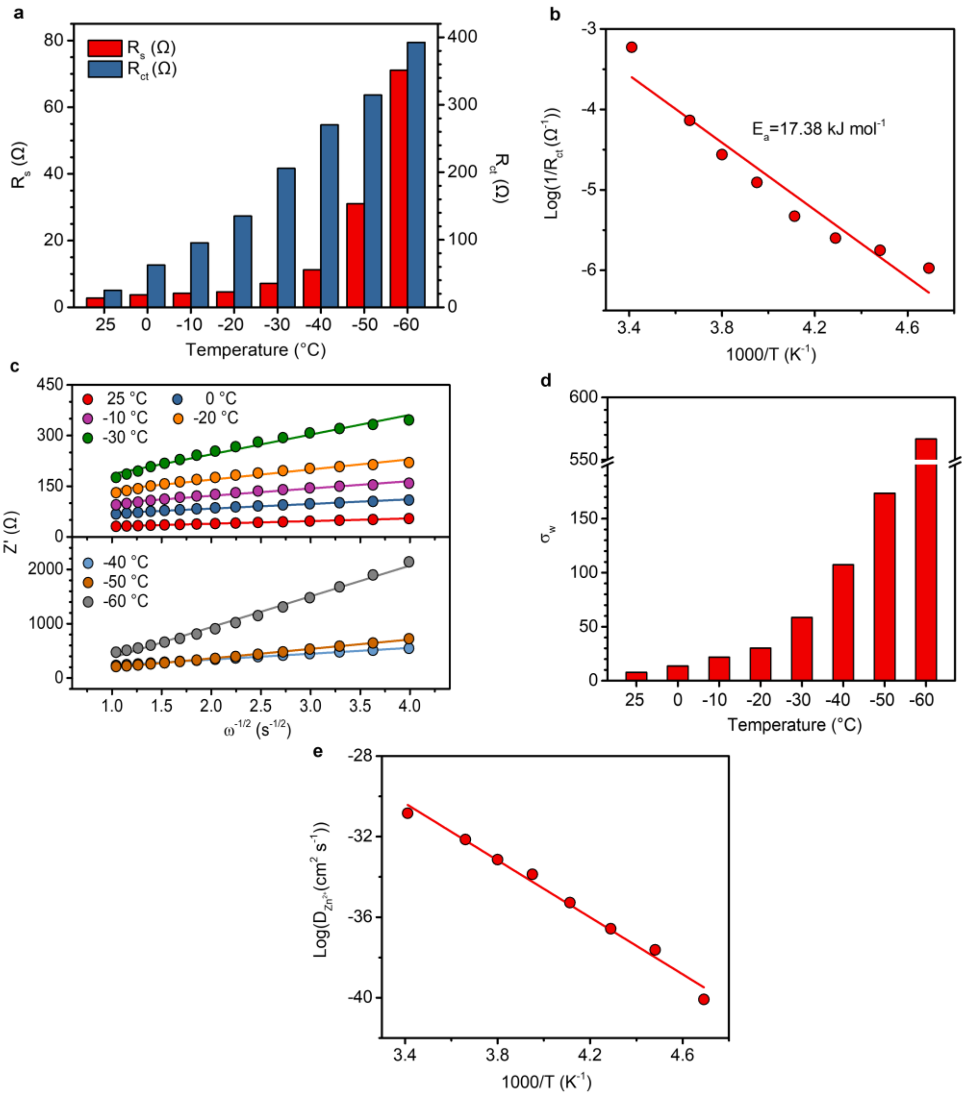

Fig. S31 Electrochemical impedance analysis of the Zn||CNTs hybrid capacitor in the temperature range of 25~–60 °C. (**a**) Comparison of internal resistance (R_s) and charge-transfer resistance (R_{ct}) of the hybrid capacitor at different temperatures. Values are obtained by fitting the EIS plots in Fig. 4c. (**b**) Linear fitting between log ($1/R_{ct}$) and 1000/T. The linear fitting indicates that the electrode/electrolyte interface of hybrid capacitor is well maintained at low temperatures. (**c**) Linear fitting between the real impedance (Z') and the inverse square root of angular frequency ($\omega^{1/2}$, $\omega=2\pi f$) at different temperatures. (**d**) Warburg factors (σ_w) at different temperatures. Values of σ_w correspond to the slope of fitted lines in (c). (**e**) Linear fitting between log (D_{Zn2+}) and 1000/T. The diffusion coefficients of Zn^{2+} (D_{Zn2+}) at different temperatures are calculated based on the Warburg diffusion area. The linear relationship between log (D_{Zn2+}) and 1000/T obeys Arrhenius law, reflecting an efficient Zn^{2+} diffusion in the electrode at low temperatures between 25 and –60 °C

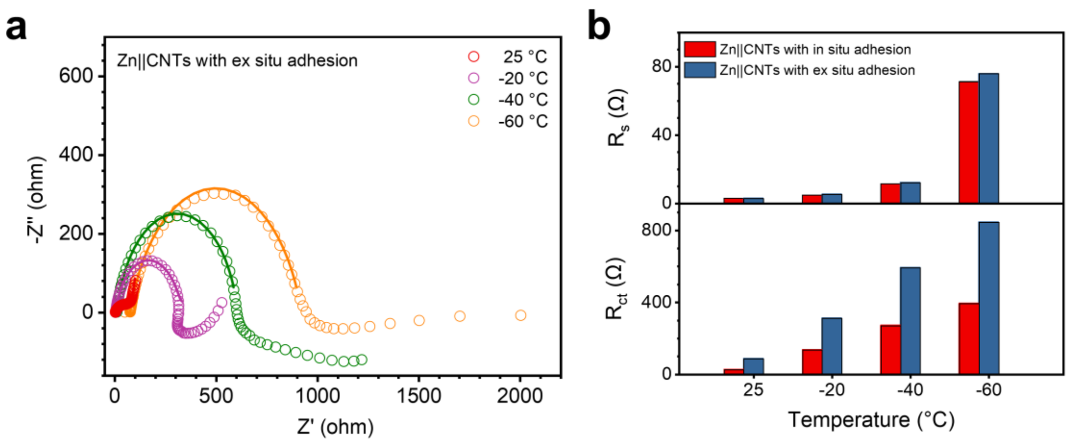

Fig. S32 Electrochemical impedance analysis of the Zn||CNTs hybrid capacitor with ex situ adhesion in the temperature range of 25~–60 °C. (**a**) Temperature-dependent Nyquist plots of the hybrid capacitor with ex situ adhesion. The solid lines correspond to the fitting equivalent circuits. (**b**) Comparison of internal resistance (R_s) and charge-transfer resistance (R_{ct}) of the hybrid capacitors with in situ adhesion and ex situ adhesion at different temperatures

We assembled another type of Zn||CNTs hybrid capacitor by directly stacking the hydrogel electrolyte between Zn and CNTs electrodes (defining as 'the Zn||CNTs hybrid capacitor with ex situ adhesion'), to be compared with the Zn||CNTs hybrid capacitor with in situ adhesion. The temperature-dependent Nyquist plots and the fitting equivalent circuit results are shown in Fig. S32a,b. We compare the fitting results between the Zn||CNTs hybrid capacitors with ex situ adhesion and in situ adhesion, as shown in Table S6.

Apparently, when the temperature drops from 25 to –60 °C, the internal resistance (R_s) of the hybrid capacitor with ex situ adhesion presents almost same value as that of the hybrid capacitor with in situ adhesion, which indicates that the anti-freezing hydrogel electrolyte contributes to ions transport at low temperatures. In contrast, the charge-transfer resistance (R_{ct}) of the hybrid capacitor with ex situ adhesion is more than twice as that of the hybrid capacitor with in situ adhesion at each temperature. This significant growth means that the charge transfer is impeded at the interface formed by ex situ adhesion, which could be attributed to a weaker interfacial contact between hydrogel electrolyte and electrode. Moreover, Nyquist plot of the hybrid capacitor with ex situ adhesion exhibits a larger slope at each temperature, indicating that the ions diffusion in the electrode could also be affected by the weak interfacial contact formed by ex situ adhesion.

Therefore, EIS results suggest that compared with the weak interface formed by ex situ adhesion, the robust interface formed by in situ adhesion significantly facilitates efficient charge transfer at the interface and ions diffusion in the electrode at low temperatures, which is beneficial to improving the electrochemical performance of the hybrid capacitor across a temperature range of 25~–60 °C.

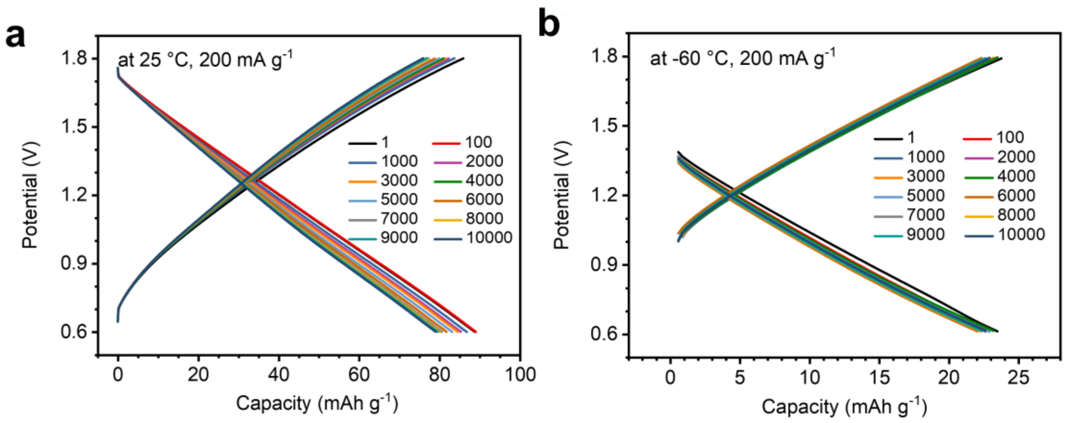

Fig. S33 Cycling life tests for the Zn||CNTs hybrid capacitor at different temperatures. Charge/discharge curves of the hybrid capacitor over 10,000 charge/discharge cycles at (**a**) 25 °C and (**b**) –60 °C with 200 mA g^{-1}

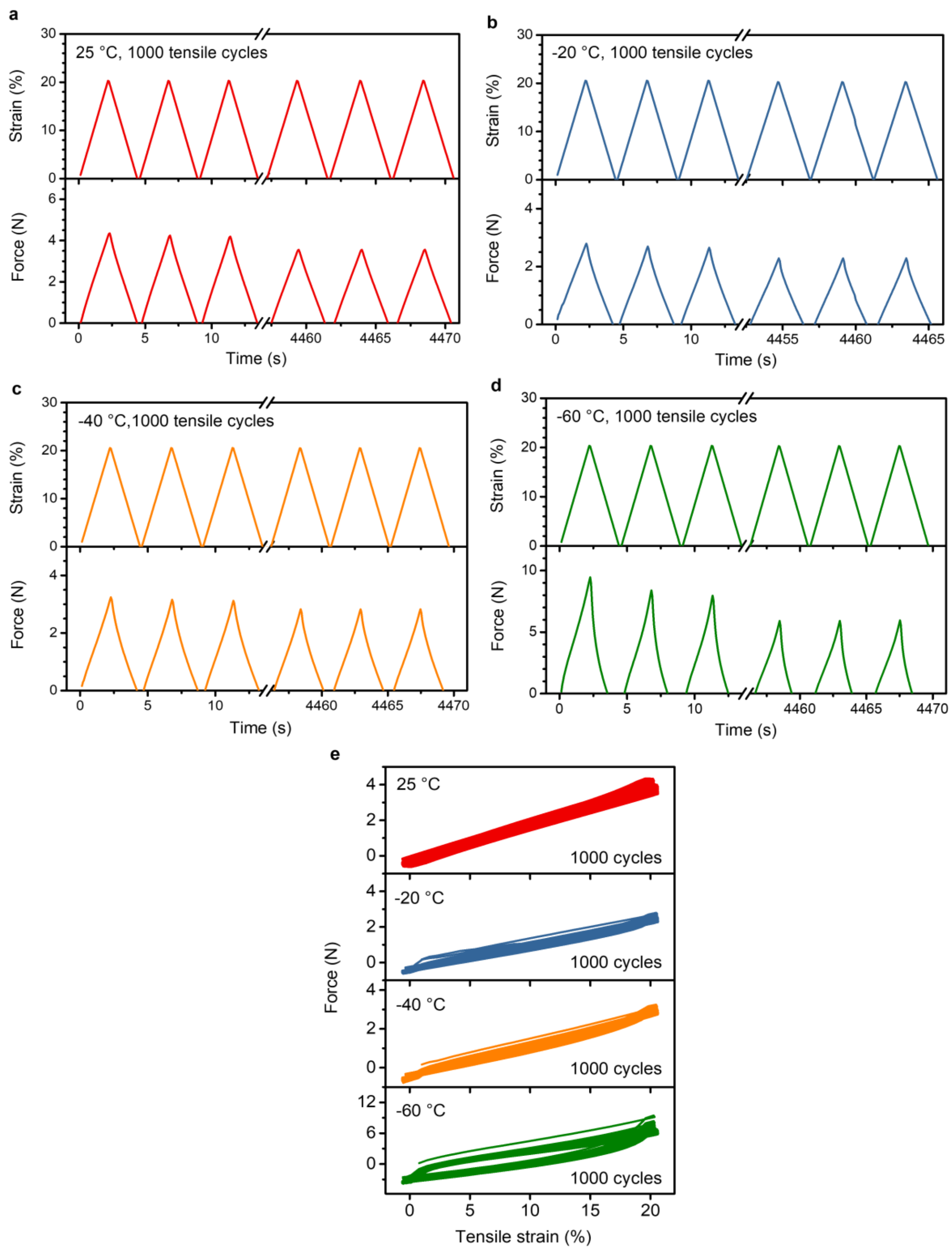

Fig. S34 Cyclic tension tests for the Zn||CNTs hybrid capacitor at 20% strain. Force and strain versus time curves of the hybrid capacitor for 1000 tension cycles at (**a**) 25, (**b**) –20, (**c**) –40, and (**d**) –60 °C, respectively. (e) Force versus strain curves of the hybrid capacitor under 20% strain for 1000 tension cycles at 25, –20, –40, and –60 °C

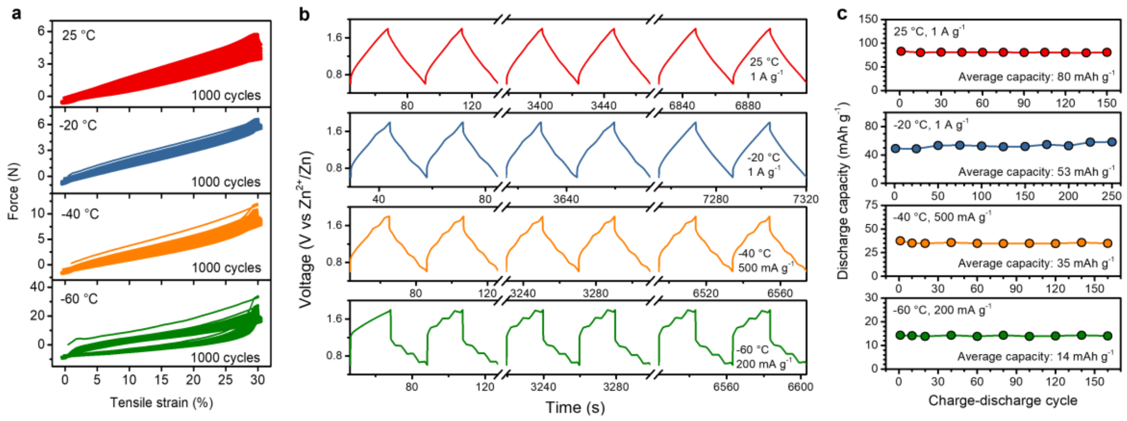

Fig. S35 Mechanical and capacitive behavior of the Zn||CNTs hybrid capacitor under tension cycles of 30% strain in the temperature range of 25~–60 °C. (**a**) Force versus strain curves of the hybrid capacitor under 30% strain for 1000 tension cycles at 25, –20, –40, and –60 °C. (**b**) Charge/discharge traces of the hybrid capacitor undergoing 1000 tension cycles of 30% strain at different temperatures. (**c**) Corresponding changes in discharge capacities of the hybrid capacitor during cyclic tension at different temperatures. The hybrid capacitor shows slight capacity degradation of less than 7.7% over 1000 tension cycles in the temperature of 25~–60 °C, achieving stable operation during dynamic deformations at low temperatures.

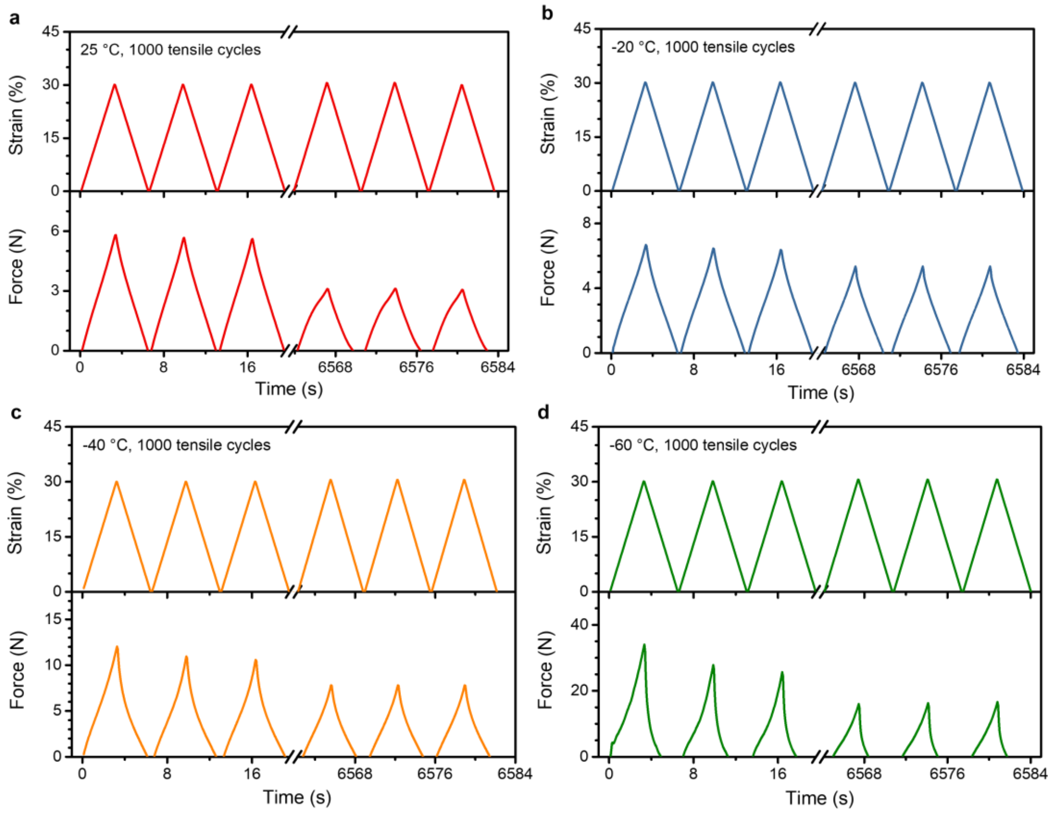

Fig. S36 Cyclic tension tests for the Zn||CNTs hybrid at 30% strain. Force and strain versus time curves of the hybrid capacitor for 1000 tension cycles at (**a**) 25, (**b**) –20, (**c**) –40, and (**d**) –60 °C

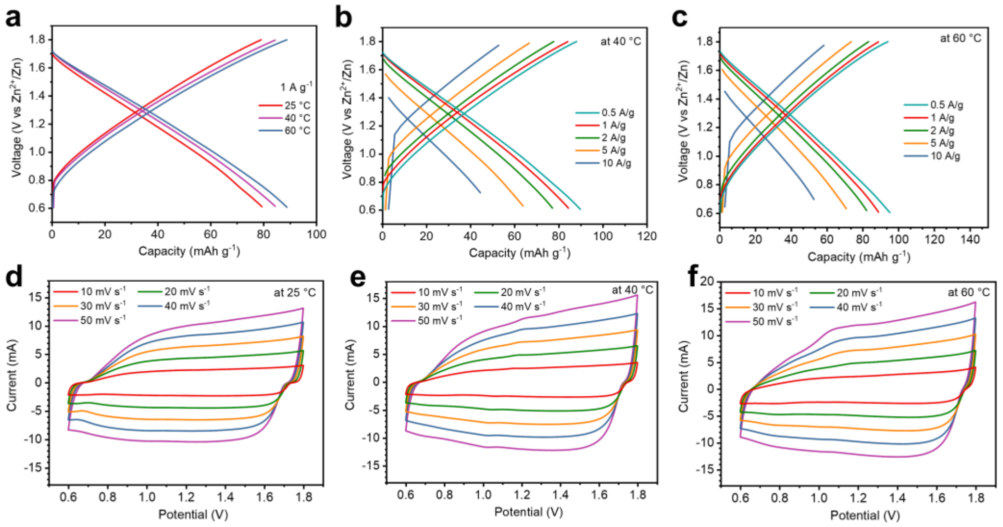

Fig. S37 High-temperature electrochemical performance of the Zn|hydrogel electrolyte|CNTs hybrid capacitor. (**a**) Charge/discharge curves of the Zn||CNTs hybrid capacitor in the temperature range of 25~60 °C. Charge/discharge curves of the Zn||CNTs hybrid capacitor with different current densities of 0.5~10 A g^{-1} at (**b**) 40 °C and (**c**) 60 °C. CV curves of the hybrid capacitor in the scan rates of 10~50 mV s^{-1} at (**d**) 25 °C, (e) 40 °C and (f) 60 °C

To investigate the high-temperature electrochemical performance of the Zn|hydrogel electrolyte|CNTs hybrid capacitor, we performed electrochemical tests of the Zn||CNTs hybrid capacitor in the temperature range of 25~60 °C. As shown in Fig. S37a, when the temperature rises from 25 °C to 60 °C, the Zn||CNTs hybrid capacitor delivers the discharge capacity of 79, 84, and 89 mAh g^{-1} with the current density of 1 A g^{-1} at 25, 40 and 60 °C, demonstrating slight capacity growth at high temperatures.

Meanwhile, the GCD curves with different current densities of 0.5~10 A g^{-1} show symmetric triangular shapes at both 40 °C and 60 °C (Fig. S37b,c), revealing stable capacity output and satisfying rate capability at high temperatures. Similarly, the CV curves in the scan rates of 10~50 mV s^{-1} maintain quasi-rectangular shapes at 25~60 °C (Fig. S37d-f), indicating the excellent reversible capacitive behavior at high temperatures.

Combining the impressive low-temperature capacitive performance we have discussed, the Zn||CNTs hybrid capacitor based on the hydrogel electrolyte achieves distinguished wide-temperature adaptability, operating stably across a wide temperature range of –60 to +60 °C.

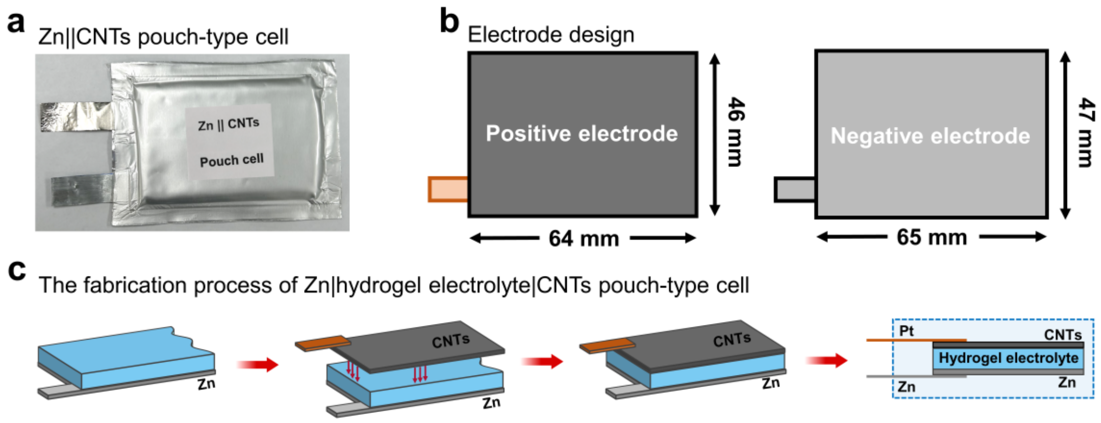

Fig. S38 Fabrication process of the Zn|hydrogel electrolyte|CNTs pouch-type cell. The image of the as-prepared Zn||CNTs pouch-type cell is presented in Fig. S38a. First, the silanized Zn plate with length 65 mm and width 47 mm is designed as the negative electrode, and the CNTs paper loaded on the carbon cloth with length 64 mm and width 46 mm is served as the positive electrode (Fig. S38b). Afterwards, the precursor solution of the hydrogel electrolyte is in situ polymerized onto the Zn and CNTs electrodes to form the Zn|hydrogel electrolyte|CNTs stack. Finally, this entire stack is sealed with aluminum-plastic film, thus obtaining the Zn||CNTs pouch-type cell (Fig. S38c)

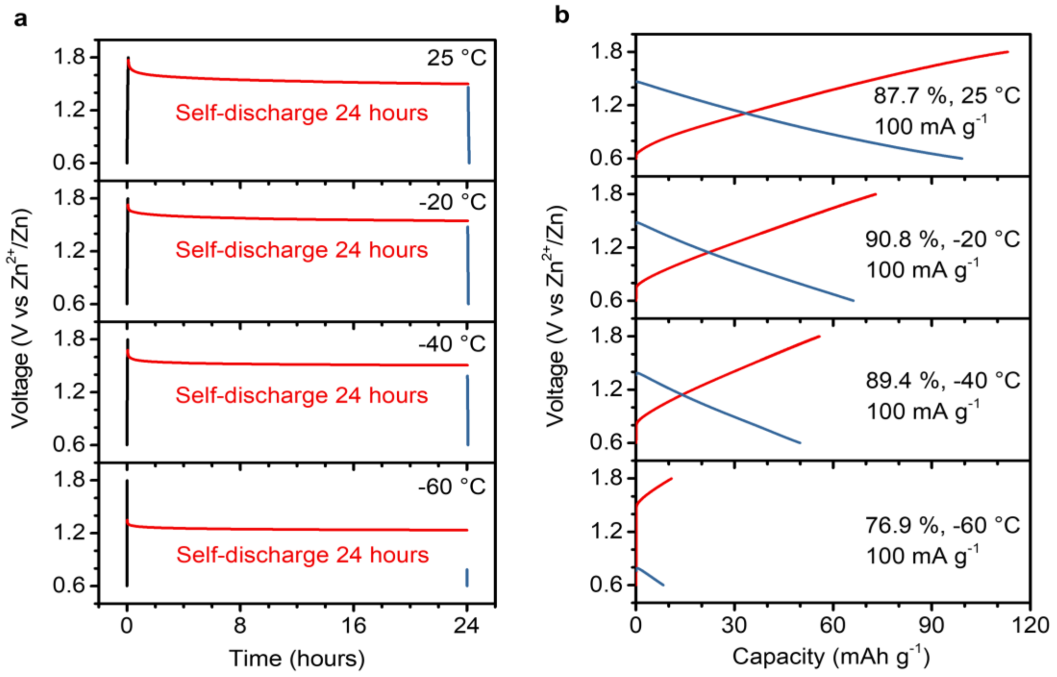

Fig. S39 Self-discharge behavior of the Zn|hydrogel electrolyte|CNTs pouch-type cell in the temperature range of 25~–60 °C. (a) Voltage versus time curves of the pouch cell with a current density of 100 mA g^{-1} at 25, –20, –40, and –60 °C. (b) Charge/discharge curves after self-discharging for 24 hours at different temperatures. The pouch cell remains ~88%, ~91%, ~89%, and ~77% of the initial capacity after resting 24 hours at 25, –20, –40, and –60 °C, respectively, exhibiting high capacity retentions at low temperatures

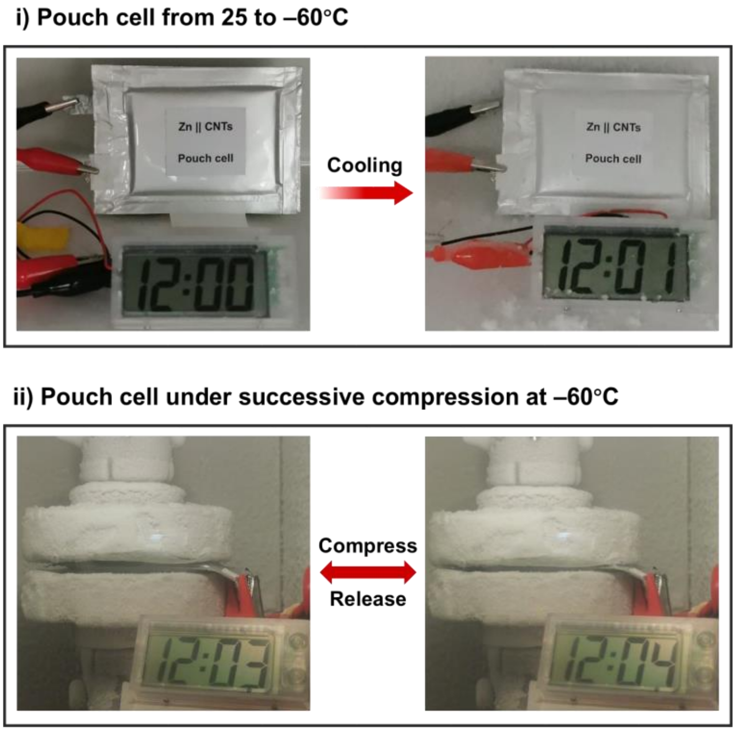

Fig. S40 Demonstration of the Zn|hydrogel electrolyte|CNTs pouch-type cell providing power in harsh conditions. The pouch cell can function well at (i) –60 °C, and (ii) under successive compression at –60 °C

Table S1 Ionic conductivities of hydrogel electrolytes with different $ZnCl_2$:LiCl molality ratios in the temperature range of 25~–80 °C

Temperature (°C)	Ionic conductivities (mS cm^{-1})					
	5:0	5:3	5:6	5:9	5:12	5:15
**25**	17.73	27.07	37.95	37.82	48.38	33.34
**0**	9.17	14.17	29.32	31.35	37.49	21.60
**–10**	4.79	8.56	14.06	14.99	20.61	12.88
**–20**	3.71	5.82	8.29	9.45	12.67	10.70
**–30**	1.95	3.53	5.80	6.72	7.73	5.23
**–40**	5.23×10^{-1}	1.87	2.96	3.81	4.25	3.17
**–60**	1.51×10^{-2}	2.93×10^{-1}	5.47×10^{-1}	9.16×10^{-1}	1.03	7.39×10^{-1}
**–80**	7.97×10^{-5}	1.36×10^{-2}	6.61×10^{-2}	7.61×10^{-2}	1.18×10^{-1}	5.73×10^{-2}

Table S2 Activation energies of ionic conduction in hydrogel electrolytes with different $ZnCl_2$:LiCl molality ratios at various temperature stages

Hydrogel electrolyte	Activation energy (eV)	
	Stage I (25~–20 °C)	Stage II (–30~–80 °C)
**$ZnCl_2$:LiCl=5:0**	0.285	0.840
**$ZnCl_2$:LiCl=5:3**	0.280	0.471
**$ZnCl_2$:LiCl=5:6**	0.272	0.382
**$ZnCl_2$:LiCl=5:9**	0.253	0.381
**$ZnCl_2$:LiCl=5:12**	0.241	0.357
**$ZnCl_2$:LiCl=5:15**	0.214	0.385

Table S3 Comparison of the anti-freezing property of our hydrogel electrolyte with previously-reported anti-freezing hydrogel electrolytes, in terms of conductivity and mechanical cyclability

Type	Hydrogel electrolyte	Tension cycles (strain) (at temperature)	Maximum Stress remaining	Conductivity (mS cm^{-1})
**Salty-dissolved hydrogel**	Chitosan-PAAm/H_3PO_4 hydrogel [S9]	10 (1000%) (25°C)	~88%	~12.6 (–60°C)
	CG/PAAm-Li/K hydrogel [S10]	10 (300%) (–40°C)	~78%	19 (–40°C)
		10 (300%) (25°C)	~73%	87 (25°C)
	CNF-Gelatin-Betaine-PAAm hydrogel [S11]	10 (70%) (–40°C)	~58%	12.4 (–40°C)
		10 (70%) (25°C)	~83%	15 (25°C)
	PHEAA/SF-LiCl hydrogel [S12]	20 (200%) (–30°C)	~81%	4.4 (25°C)
	PVA/LiCl hydrogel [S13]	10 (200%) (25°C)	~80%	~112 (25°C) ~40 (–40°C)
	CS-P(AM-co-AA)/$FeCl_3$ hydrogel [S14]	1000 (100%) (25°C)	~65%	0.1 (–20°C)
	PANI/CS-PAAm Hydrogel [S15]	1000 (50%) (25°C)	~100%	~1 (–30°C)
	PVA/ $Fe_2(SO_4)_3$ Hydrogel [S16]	50 (200%) (25°C)	~80%	6 (25°C)
	CS-PHEAA Hydrogel [S17]	60 (200%) (–60°C)	~74%	1 (25°C)
	G-CyBA/PAAm SP-KOH hydrogel [S18]	10 (500%) (25°C)	~38%	63.2 (–80°C)
**Organic hydrogel**	PBA/CPA/Gly hydrogel [S19]	20 (500 %) (25°C)	~100%	0.29 (–40°C)
	PMZn-GL hydrogel [S20]	5 (60%) (25°C)	~94%	0.56 (25°C)

	P(AAm/AMPS)-AFPS hydrogel [S21]	10 (500%) (25°C)	~92%	7 (–10°C)
	XG-Fe^{3+}/PAAm-Gl hydrogel [S22]	1000 (100%) (25°C)	~55%	5.1 (25°C)
**This work**	**PAAm-CSA/$ZnCl_2$-LiCl hydrogel**	**100 (100%) (25°C)**	**90.89%**	**48.38 (25°C)**
		**100 (100%) (–20°C)**	**78.43%**	**12.61 (–20°C)**
		**100 (100%) (–40°C)**	**73.01%**	**4.25 (–40°C)**
		**100 (100%) (–60°C)**	**69.05%**	**1.03 (–60°C)**

Table S4 Comparison of the low-temperature adhesion performance of our hydrogel electrolyte with previously-reported anti-freezing hydrogels

Type	Hydrogel	Freezing tolerance (°C)	Shear strength (kPa)
**Salty-dissolved hydrogel**	PHEAA-SF/LiCl hydrogel [S12]	−30	45.3 (25°C)
	PEG/NaCl hydrogel [S23]	−80	61 (25°C)
	PVA-PAM-MXene/$CaCl_2$ Hydrogel [S24]	−50	12 (25°C)
	PVA/LiCl hydrogel [S13]	−40	25 (25°C)<br>190 (−40°C)
	TC-PSA/$ZnCl_2$ hydrogel [S25]	−60	31.5 (25°C)<br>98 (−60°C)
	CNF-PAA/$ZnSO_4$ Hydrogel [S26]	−40	25.5 (25°C)<br>111 (−40°C)
**Organic hydrogel**	Gluten-based hydrogel [S27]	−20	35.4 (25°C)<br>32 (−20°C)
	Poly(AAm-co-APMA)/GO-Gly hydrogel [S28]	−20	277.3 (−20°C)
	AU/PAA hydrogel [S29]	−20	8 (25°C)<br>6 (−20°C)
	PVA-P(AM-*co*-SBMA)/$CaCl_2$ hydrogel [S30]	−40	12.3 (25°C)<br>30.3 (−40°C)
	PDA/CNTs/GW hydrogel [S31]	−20	57 (25°C)<br>67.5 (−20°C)
	Dopamine/PSBMA hydrogel [S32]	0	20.4 (25°C)
	KOH-filled SP-DN hydrogel [S33]	-50	1.3 (25°C)<br>1.6 (–40°C)
	PAM-EG/W/H_2SO_4 hydrogel [S34]	−30	10.5 (25°C)
**Zwitterionic hydrogel**	PBA/CPA/Gly hydrogel [S19]	−40	11.5 (25°C)<br>24 (−40°C)

	SA-PAAm-SBMA/$Zn(CF_3SO_3)_2$ hydrogel [S35]	−20	4.1 (25°C)
	P(AM-co-SBMA)-$Zn(OTf)_2$ hydrogel [S36]	25	19 (25°C)
**This work**	**PAAm-CSA/$ZnCl_2$-LiCl hydrogel**	**25**	**73.88 (25°C)**
		**−20**	**103.38 (−20°C)**
		**−40**	**236.17 (−40°C)**
		**−60**	**347.39 (−60°C)**

Table S5 Fitting equivalent circuit results of the Zn||CNTs hybrid capacitor in the temperature range of 25~−60 °C

Temperature (°C)	Equivalent circuit	R_s (Ω)	R_{ct} (Ω)	$D_{Zn^{2+}}$ (cm^{-2} s^{-1})
25		2.78	25.21	4.06×10^{-14}
0		3.75	62.56	1.10×10^{-14}
−10		4.20	95.66	4.05×10^{-15}
−20	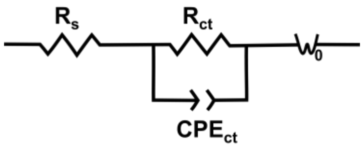	4.63	135.20	1.95×10^{-15}
−30		7.15	205.90	4.79×10^{-16}
−40		11.26	270.20	1.31×10^{-16}
−50		31.04	314.52	4.58×10^{-17}
−60		71.08	392.60	3.92×10^{-18}

Table S6 Comparison of fitting equivalent circuit results between the Zn||CNTs with ex situ adhesion and in situ adhesion in the temperature range of 25~−60 °C

Zn||CNTs hybrid capacitor	Equivalent circuit	Temperature (°C)	R_s (Ω)	R_{ct} (Ω)
**Zn||CNTs with ex situ adhesion**	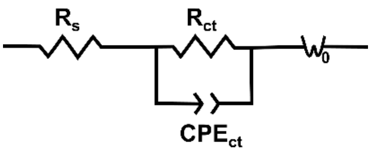	25	2.91	85.98
		−20	5.26	310.40
		−40	12.10	592.36
		−60	75.88	845.60
**Zn||CNTs with in situ adhesion**		25	2.78	25.21
		−20	4.63	135.20
		−40	11.26	270.20
		−60	71.08	392.60

Table S7 Summary of specific capacitances, energy densities and power densities of the Zn||CNTs hybrid capacitor in the temperature range of 25~–80 °C

Temperature (°C)	Current density ($A\ g^{-1}$)	Capacity ($mAh\ g^{-1}$)	Capacitance ($F\ g^{-1}$)	Energy density ($Wh\ kg^{-1}$)	Power density ($W\ kg^{-1}$)
**25**	5	23.30	69.89	15.10	41801.89
	2	43.01	129.03	42.42	25454.60
	1	52.69	158.06	59.16	14488.38
	0.5	62.19	186.56	74.94	7773.90
	0.2	72.62	217.85	91.68	3258.94
	0.1	80.65	241.94	103.90	1662.76
**0**	5	12.54	37.63	5.41	27803.13
	2	33.69	101.08	29.28	22423.87
	1	44.09	132.26	46.20	13522.00
	0.5	52.87	158.60	61.40	7492.92
	0.2	62.37	187.10	77.46	3205.93
	0.1	68.81	206.44	87.66	1644.03
**–10**	5	8.96	26.88	3.25	23419.52
	2	28.67	86.02	23.51	21159.54
	1	38.35	115.05	38.57	12978.35
	0.5	46.24	138.71	52.40	7311.74
	0.2	56.27	168.82	69.24	3176.29
	0.1	59.96	179.89	75.68	1628.89
**–20**	2	23.66	70.97	17.59	19189.38
	1	33.69	101.08	32.25	12350.98
	0.5	41.76	125.27	45.81	7076.88
	0.2	50.97	152.90	61.67	3122.93
	0.1	54.44	163.33	67.91	1609.84
**–30**	2	13.62	40.86	7.07	13397.09
	1	25.81	77.42	21.05	10525.59
	0.5	34.41	103.23	34.32	6434.73
	0.2	42.72	128.17	48.94	2956.08
	0.1	50.65	151.94	62.33	1588.53
**–40**	1	16.13	48.39	9.80	7840.31
	0.5	26.88	80.64	23.47	5633.92
	0.2	37.78	113.33	41.32	2822.48
	0.1	41.50	124.51	48.42	1505.69
**–50**	0.5	12.02	36.07	6.77	2512.10
	0.2	23.01	69.03	20.62	1599.49
	0.1	30.54	91.61	33.27	1033.64
	0.05	34.52	103.55	40.77	527.17
	0.02	38.74	116.21	49.19	226.41
**–60**	0.2	12.69	38.06	7.70	1026.67
	0.1	19.53	58.60	16.04	723.57
	0.05	25.79	77.38	27.97	483.91
	0.02	31.48	94.44	38.61	218.74
**–70**	0.05	12.98	38.93	8.42	289.52
	0.02	21.74	65.82	22.25	180.84
	0.01	27.67	83.02	32.20	104.02
	0.0075	30.00	90.00	36.88	82.51
	0.005	32.02	96.07	40.68	56.92
**–80**	0.01	11.07	33.22	7.69	61.93
	0.0075	14.57	43.71	11.97	54.91
	0.005	19.19	57.56	17.71	41.34

Table S8 Comparison of electrochemical performance of our hybrid capacitor with previously-reported low-temperature aqueous-based energy storage devices

Type	Device configuration	Electrolyte	Freezing tolerance	Cycle life	Max. energy (Wh kg^{-1}) @ power (W kg^{-1})	Energy (Wh kg^{-1}) @ max. power (W kg^{-1})	Capacity retention under deformation
Symmetrical supercapacitor	CNT||CNT[37]	PVA/H_2O/EG/LiCl organohydrogel	–40 °C	88.3% over 5000 cycles at –20 °C	—	—	90.1% under 1000 180°-bending cycles at 25 °C
	AC||AC[38]	PAMPS-PAAm/EG/LiCl hydrogel	–20 °C	97.9% over 10,000 cycles at –20 °C	6@999 (–20 °C)	—	Stable under 100 kg-crushing or different bending angles at 25 °C
	PANI/carbon cloth|| PANI/carbon	PVA-PAAm/H_2SO_4	–40 °C	—	16@1433 (25 °C)	—	86.5% under 4,000 180°-bending cycles at –30 °C
	CNT||CNT[40]	SF/EMImAc/H_2O/ KCl hydrogel	–50 °C	99.3% over 1000 cycles at	—	—	Stable under knotting state at –50 °C
	Graphene||graphene	MMT/PVA/DSMO /H_2SO_4 hydrogel	–50 °C	95% over 10,000 cycles	—	—	91% under 1000 bending cycles at 25 °C
	AC||AC[9]	Chitosan-PAAm/H_3PO_4 hydrogel	–60 °C	97.5% over 10,000 cycles at 25 °C	15@50 (25 °C)	8@2311 (25 °C)	Stable under 1000 180°-bending or compression cycles at 25 °C
	AC||AC[42]	EGINA-PVA-PHEAA/LiCl	–20 °C	56.3% over 2000 cycles at	—	—	—

	AC||AC[43]	Poly(SBMA-*co*-HEA)/LiCl	–30 °C	71% over 10,000 cycles	—	—	78.5% under 500 twisting cycles at 25 °C
	AC||AC[44]	Poly(HFBA-*co*-HEMA)/LiTFSI	–20 °C	76% over 8000 cycles at	—	—	100% under 500 180°-bending cycles at 25 °C
	MWCNT/RGO|| MWCNT/RGO[45]	PC-PMMA/ [BMIM][TFSI] gel	–30 °C	95.2% over 5000 cycles at –30 °C	4 µWh cm^{-2}@12 µW cm^{-2} (25 °C) 4 µWh cm^{-2}@12 µW	2 µWh cm^{-2}@800 µW cm^{-2} (25 °C) 0.2 µWh cm^{-2}@400	97.6% under 1000 30% tension cycles at 25 °C
	AC||AC[46]	Poly(AAm-SBMA)/EG/H_2SO_4	–70 °C	81.5% over 10,000 cycles	—	—	—
**Zn-ion battery**	Zn||NiCo[47]	PANa/Zn$(CH_3COO)_2$ hydrogel	–20 °C	87% over 10,000 cycles	172 (–20 °C)	6800 (–20 °C)	97.3% under different bending angles from 30° [illegible]
	Zn||MnO_2-CNT[48]	EG-waPUA-PAAm/$ZnSO_4$-$MnSO_4$	–20 °C	74.54% over 600 cycles at [illegible]	350@650 (25 °C)	210@2100 (25 °C)	Stable under bending, compression, or hammering at –20 °C
	Zn||$NH_4V_3O_8$[49]	Xanthan gum/$ZnCl_2$ gel	–20 °C	83% over 450 cycles at	—	—	Stable under 90° and 180°-bending cycles at
	Zn||PANI[50]	$ZnCl_2$ aqueous solution	–90 °C	~100% over 2000 cycles at	98 (25 °C)	—	—
	Zn||PANI-V_2O_5[51]	Water/EG/$ZnSO_4$	–40 °C	Stable over 250 cycles at	121 (–20 °C)	1700 (–20 °C)	—

	Zn‖$LiFePO_4$[52]	PAAm/$ZnSO_4$-LiCl hydrogel	–20 °C	~100% over 500 cycles at	—	—	>80% under 400 bending cycles at –20 °C
	Zn‖MnO_2[53]	Guar gum-alginate/EG/$ZnSO_4$-$MnSO_4$ hydrogel	–20 °C	80.39% over 100 cycles at –20 °C	432@208 (25 °C)<br>234@133 (–20 °C)	111@7430 (25 °C)<br>141@665 (–20 °C)	97.58% under bending state at –20 °C
	Zn‖V_2O_5[54]	$Zn(CF_3SO_3)_2$ aqueous solution	–30 °C	81.7% over 1000 cycles at 30 °C	—	—	—
	Zn‖MnHCF[55]	PVHF/MXene-*g*-PMA polymer	–35 °C	Stable over 90 days at	—	—	—
	Zn‖E-Bi_2Se_3[56]	HC-EGPAm/$Zn(TFSI)_2$ hydrogel	–40 °C	94.6% over 2000 cycles at 0 °C	400@390(25 °C)<br>441@683 (–20 °C)	200@3800 (25 °C)<br>210@4000 (–20 °C)	—
**Zn-ion hybrid capacitor**	Zn‖AC[57]	Cellulose/$ZnCl_2$ Hydrogel	–20 °C	94.7% over 5000 cycles at 25 °C	192@499 (25 °C)	58@16976 (25 °C)	Stable under 500 bending cycles at 25 °C
	Zn‖porous carbon[58]	PVA/$Zn(CF_3SO_3)_2$ hydrogel	–15 °C	~74% over 10,000 cycles at –15 °C	147@136 (25 °C)	65@15700 (25 °C)	Stable under different bending states at 25 °C
	Zn‖N-doped AC[51]	Water/EG/$ZnSO_4$ solution	–40 °C	Stable over 5000 cycles at –20 °C	80@85 (25 °C)<br>36@80 (–20 °C)	45@17000 (25 °C)<br>20@3100 (–20 °C)	—
	Zn‖AC[59]	Water/EG/$ZnSO_4$ solution	–40 °C	97.8% over 1000 cycles at 20 °C	—	—	—

	Zn‖AC[60]	$Zn(ClO_4)_2$ salty ice	–60 °C	Operating over 280 days at –30 °C	40.55 (–60 °C)	—	—
	Zn‖porous carbon[61]	PAAm/$ZnCl_2$ Hydrogel	–20 °C	92.9% over 40,000 cycles at –20 °C	217@450 (25 °C)	65@18000 (25 °C)	Stable under 400 bending cycles at 25 °C
	Zn‖graphene[62]	PVA/EG/$Zn(Tf)_2$ gel	–20 °C	Stable over 30,000 cycles at –20 °C	137@200 (25 °C)<br>158@237 (–20 °C)	57@94000 (25 °C)	91.8% under 300 bending cycles at 25 °C
	Zn‖AC[25]	TC-P(SBMA-*co*-AA)/$ZnCl_2$ hydrogel	–60 °C	84.6% over 100,000 cycles at –40 °C	253@690 (25 °C)<br>81@720 (–40 °C)	176@27500 (25 °C)<br>40@27800 (–40 °C)	88% under 100 bending cycles at –40 °C
**Zn/Li hybrid capacitor**	**Zn‖CNTs this work**	**PAAm-CSA/$ZnCl_2$-LiCl hydrogel**	**–60 °C**	**98.7% over 10,000 cycles at –60 °C**	**104@1663 (25 °C)**<br>**39@219 (–60 °C)**	**15@41802 (25 °C)**<br>**8@1027 (–60 °C)**	**>90% under 1000 20% tension cycles at 25~–60 °C;**<br>**>84% under 1000**

Description of Supporting Movies

Movie S1: Demonstration for adhesion tests of the hydrogel electrolyte/Zn hybrids, hydrogel electrolyte/CNTs hybrids, and Zn/hydrogel electrolyte/CNTs hybrids. First column: 90-degree peel tests of various hydrogel electrolyte/Zn hybrids with different AAm:CSA mass ratios. Second column: 180-degree peel tests of various hydrogel electrolyte/CNTs hybrids with different AAm:CSA mass ratios. Third column: tensile tests of various Zn/hydrogel electrolyte/CNTs hybrids with different AAm:CSA mass ratios.

Movie S2: Simulated peeling processes of the tough hydrogel and the pure elastic hydrogel from rigid substrates.

Movie S3: Demonstration for testing processes of mechanical and capacitive behaviors of the Zn||CNTs hybrid capacitor under 20% and 30% cyclic tensions at 25 °C and –60 °C.

Movie S4: Working performance of the Zn||CNTs pouch-type cells at 25 and –60 °C. When the environmental temperature drops from 25 to –60 °C, the pouch cell is able to continue powering an electronic watch, and the five pouch cells in series enable a flash lamp to work well.

Movie S5: Demonstration for the Zn||CNTs pouch-type cells providing power at harsh conditions. Both a pouch cell and five pouch cells in series can function well under successive compressions at –60 °C. When being punctured and cut, the pouch cell can still power an electronic watch.

Supplementary References

[S1] H. Yuk, C.E. Varela, C.S. Nabzdyk, X. Mao, R.F. Padera, et al. Dry double-sided tape for adhesion of wet tissues and devices. Nature. **575**(7781), 169-174 (2019). https://doi.org/10.1038/s41586-019-1710-5

[S2] H. Yuk, T. Zhang, S. Lin, G.A. Parada, X. Zhao. Tough bonding of hydrogels to diverse non-porous surfaces. Nat. Mater. **15**(2), 190-196 (2016). https://doi.org/10.1038/NMAT4463

[S3] H. Yuk, T. Zhang, G.A. Parada, X. Liu, X. Zhao. Skin-inspired hydrogel–elastomer hybrids with robust interfaces and functional microstructures. Nat. Commun. **7**(1), 1-11 (2016). https://doi.org/10.1038/ncomms12028

[S4] H.J. Berendsen, D. van der Spoel, R. van Drunen. Gromacs: A message-passing parallel molecular dynamics implementation. Comput. Phys. Commun. **91**(1-3), 43-56 (1995). https://doi.org/10.1016/0010-4655(95)00042-E

[S5] W.L. Jorgensen, D.S. Maxwell, J. Tirado-Rives. Development and testing of the opls all-atom force field on conformational energetics and properties of organic liquids. J. Am. Chem. Soc. **118**(45), 11225-11236 (1996). https://doi.org/10.1021/ja9621760

[S6] W.L. Jorgensen, J. Chandrasekhar, J.D. Madura, R.W. Impey, M.L. Klein. Comparison of simple potential functions for simulating liquid water. J. Chem. Phys. **79**(2), 926-935 (1983). https://doi.org/10.1063/1.445869

[S7] J. Zheng, G. Tan, P. Shan, T. Liu, J. Hu, et al. Understanding thermodynamic and kinetic contributions in expanding the stability window of aqueous electrolytes. Chem-us. **4**(12), 2872-2882 (2018). https://doi.org/10.1016/j.chempr.2018.09.004

[S8] Q. Zhang, Y. Ma, Y. Lu, L. Li, F. Wan, et al. modulating electrolyte structure for ultralow temperature aqueous zinc batteries. Nat. Commun. **11**(1), 4463 (2020). https://doi.org/10.1038/s41467-020-18284-0

[S9] J. Xu, R. Jin, X. Ren, G. Gao. A wide temperature-tolerant hydrogel electrolyte mediated by phosphoric acid towards flexible supercapacitors. Chem. Eng. J. **413**(127446 (2021). https://doi.org/10.1016/j.cej.2020.127446

[S10] S. Wu, D. Lou, H. Wang, D. Jiang, X. Fang, et al. One-pot synthesis of anti-freezing carrageenan/polyacrylamide double-network hydrogel electrolyte for low-temperature flexible supercapacitors. Chem. Eng. J. **435**((2022). https://doi.org/10.1016/j.cej.2022.135057

[S11] Y. Liu, Q. Liu, L. Zhong, C. Chen, Z. Xu. Tough, antifreezing, and conductive double network zwitterionic-based hydrogel for flexible sensors. Chem. Eng. J. **452**((2023). https://doi.org/10.1016/j.cej.2022.139314

[S12] X. Sun, S. He, M. Yao, X. Wu, H. Zhang, et al. Fully-physically crosslinked silk fibroin/poly(hydroxyethyl acrylamide) hydrogel with high transparency and adhesive properties for wireless sensing and low-temperature strain sensing. J. Mater. Chem. C. **9**(6), 1880-1887 (2021). https://doi.org/10.1039/d0tc05958g

[S13] J. Yin, K. Wei, J. Zhang, S. Liu, X. Wang, et al. Mxene-based film electrode and all-round hydrogel electrolyte for flexible all-solid supercapacitor with extremely low working temperature. Cell Rep. Phys. Sci. **3**(5), (2022). https://doi.org/10.1016/j.xcrp.2022.100893

[S14] H. Liu, X. Wang, Y. Cao, Y. Yang, Y. Yang, et al. Freezing-tolerant, highly sensitive strain and pressure sensors assembled from ionic conductive hydrogels with dynamic cross-links. ACS Appl. Mater. Interfaces. **12**(22), 25334-25344 (2020). https://doi.org/10.1021/acsami.0c06067

[S15] L. Zhou, C. Dai, L. Fan, Y. Jiang, C. Liu, et al. Injectable self-healing natural biopolymer-based hydrogel adhesive with thermoresponsive reversible adhesion for minimally invasive surgery. Adv. Funct. Mater. **31**(14), (2021). https://doi.org/10.1002/adfm.202007457

[S16] Y. Gao, J. Peng, M. Zhou, Y. Yang, X. Wang, et al. A multi-model, large range and anti-freezing sensor based on a multi-crosslinked poly(vinyl alcohol)

hydrogel for human-motion monitoring. J. Mater. Chem. B. **8**(48), 11010-11020 (2020). https://doi.org/10.1039/d0tb02250k

[S17] Y. Yang, Y. Yang, Y. Cao, X. Wang, Y. Chen, et al. Anti-freezing, resilient and tough hydrogels for sensitive and large-range strain and pressure sensors. Chem. Eng. J. **403**((2021). https://doi.org/10.1016/j.cej.2020.126431

[S18] J. Xu, R. Jin, X. Ren, G. Gao. A wide temperature-tolerant hydrogel electrolyte mediated by phosphoric acid towards flexible supercapacitors. Chem. Eng. J. **413**((2021). https://doi.org/10.1016/j.cej.2020.127446

[S19] S. Hao, L. Meng, Q. Fu, F. Xu, J. Yang. Low-temperature tolerance and conformal adhesion zwitterionic hydrogels as electronic skin for strain and temperature responsiveness. Chem. Eng. J. **431**((2022). https://doi.org/10.1016/j.cej.2021.133782

[S20] Y. Feng, H. Liu, W. Zhu, L. Guan, X. Yang, et al. Muscle-inspired mxene conductive hydrogels with anisotropy and low-temperature tolerance for wearable flexible sensors and arrays. Adv. Funct. Mater. **31**(46), (2021). https://doi.org/10.1002/adfm.202105264

[S21] J. Xu, R. Jing, X. Ren, G. Gao. Fish-inspired anti-icing hydrogel sensors with low-temperature adhesion and toughness. J. Mater. Chem. A. **8**(18), 9373-9381 (2020). https://doi.org/10.1039/d0ta02370a

[S22] Q. Yu, Z. Qin, F. Ji, S. Chen, S. Luo, et al. Low-temperature tolerant strain sensors based on triple crosslinked organohydrogels with ultrastretchability. Chem. Eng. J. **404**((2021). https://doi.org/10.1016/j.cej.2020.126559

[S23] L. Chen, Z. Wang, Z. Zhan, M. Xie, G. Duan, et al. 3D printed super-anti-freezing self-adhesive human-machine interface. Mater. Today Phys. **19**, (2021). https://doi.org/10.1016/j.mtphys.2021.100404

[S24] W. Peng, X. Pan, X. Liu, Y. Gao, T. Lu, et al. A moisture self-regenerative, ultra-low temperature anti-freezing and self-adhesive polyvinyl alcohol/polyacrylamide/$CaCl_2$/MXene ionotronics hydrogel for bionic skin strain sensor. J. Colloid Interf. Sci. **634**, 782-792 (2023). https://doi.org/https://doi.org/10.1016/j.jcis.2022.12.101

[S25] Q. Fu, S. Hao, L. Meng, F. Xu, J. Yang. Engineering self-adhesive polyzwitterionic hydrogel electrolytes for flexible zinc-ion hybrid capacitors with superior low-temperature adaptability. ACS Nano. **15**(11), 18469-18482 (2021). https://doi.org/10.1021/acsnano.1c08193

[S26] Q. Fu, S. Hao, X. Zhang, H. Zhao, F. Xu, et al. All-round supramolecular zwitterionic hydrogel electrolytes enabling environmentally adaptive dendrite-free aqueous zinc ion capacitors. Energy Environ. Sci. **16**(3), 1291-1311 (2023). https://doi.org/10.1039/d2ee03793a

[S27] H. Xu, X. Jiang, X. Han, H. Cai, F. Gao. Cooking inspired tough, adhesive, and low-temperature tolerant gluten-based organohydrogels for high performance strain sensors. J. Mater. Chem. A. **9**(44), 25104-25113 (2021). https://doi.org/10.1039/d1ta07273k

[S28] R. Zhao, L. Jiang, P. Zhang, D. Li, Z. Guo, et al. Graphene oxide-based composite organohydrogels with high strength and low temperature resistance for strain sensors. Soft Matter. **18**(6), 1201-1208 (2022). https://doi.org/10.1039/d1sm01655e

[S29] X. Liu, Q. Zhang, G. Gao. DNA-inspired anti-freezing wet-adhesion and tough hydrogel for sweaty skin sensor. Chem. Eng. J. **394**, (2020). https://doi.org/10.1016/j.cej.2020.124898

[S30] O. Hu, J. Lu, S. Weng, L. Hou, X. Zhang, et al. An adhesive, anti-freezing, and environment stable zwitterionic organohydrogel for flexible all-solid-state supercapacitor. Polymer. **254**, (2022). https://doi.org/10.1016/j.polymer.2022.125109

[S31] L. Han, K. Liu, M. Wang, K. Wang, L. Fang, et al. Mussel-inspired adhesive and conductive hydrogel with long-lasting moisture and extreme temperature tolerance. Adv. Funct. Mater. **28**(3), (2018). https://doi.org/10.1002/adfm.201704195

[S32] C. Zhang, Y. Zhou, H. Han, H. Zheng, W. Xu, et al. Dopamine-triggered hydrogels with high transparency, self-adhesion, and thermoresponse as skinlike sensors. ACS Nano. **15**(1), 1785-1794 (2021). https://doi.org/10.1021/acsnano.0c09577

[S33] C. Gu, X.-Q. Xie, Y. Liang, J. Li, H. Wang, et al. Small molecule-based supramolecular-polymer double-network hydrogel electrolytes for ultra-stretchable and waterproof Zn–air batteries working from −50 to 100 °C. Energy Environ. Sci. **14**(8), 4451-4462 (2021). https://doi.org/10.1039/d1ee01134k

[S34] Z.F. Jing Cong, Shaoshan Pan, Jie Tian, Weizhen Lian, Shan Li, Sijie Wang, Dongchang Zheng, Chunguang Miao, Weiping Ding, Taolin Sun, and Tianzhi Luo. Polyacrylamide/chitosan-based conductive double network hydrogels with outstanding electrical and mechanical performance at low temperatures. ACS Appl. Mater. Interfaces. **13**(34942−34953 (2021). https://doi.org/10.1021/acsami.1c08421

[S35] T. Wu, C. Ji, H. Mi, F. Guo, G. Guo, et al. Construction of zwitterionic osmolyte-based hydrogel electrolytes towards stable zinc anode for durable aqueous zinc ion storage and integrated electronics. J. Mater. Chem. A. **10**(48), 25701-25713 (2022). https://doi.org/10.1039/d2ta07410a

[S36] W. Zhang, F. Guo, H. Mi, Z.S. Wu, C. Ji, et al. Kinetics-boosted effect enabled by zwitterionic hydrogel electrolyte for highly reversible zinc anode in zinc-ion hybrid micro-supercapacitors. Adv. Energy Mater. . **12**(40), (2022). https://doi.org/10.1002/aenm.202202219

[S37] Q. Rong, W. Lei, J. Huang, M. Liu. Low temperature tolerant organohydrogel electrolytes for flexible solid-state supercapacitors. Adv. Energy Mater. **8**(31), 1801967 (2018). https://doi.org/10.1002/aenm.201801967

[S38] X. Li, D. Lou, H. Wang, X. Sun, J. Li, et al. Flexible supercapacitor based on organohydrogel electrolyte with long-term anti-freezing and anti-drying property. Adv. Funct. Mater. **30**(52), 2007291 (2020). https://doi.org/10.1002/adfm.202007291

[S39] Z. Liu, J. Zhang, J. Liu, Y. Long, L. Fang, et al. Highly compressible and superior low temperature tolerant supercapacitors based on dual chemically crosslinked PVA hydrogel electrolytes. J. Mater. Chem. A. **8**(13), 6219-6228 (2020). https://doi.org/10.1039/c9ta12424a

[S40] N. Chang, T. Li, R. Li, S. Wang, Y. Yin, et al. An aqueous hybrid electrolyte for low-temperature zinc-based energy storage devices. Energy Environ. Sci. **13**(10), 3527-3535 (2020). https://doi.org/10.1039/d0ee01538e

[S41] C. Lu, X. Chen. All-temperature flexible supercapacitors enabled by antifreezing and thermally stable hydrogel electrolyte. Nano Lett. **20**(3), 1907-1914 (2020). https://doi.org/10.1021/acs.nanolett.9b05148

[S42] Q. Zhang, K. Xia, Y. Ma, Y. Lu, L. Li, et al. Chaotropic anion and fast-kinetics cathode enabling low-temperature aqueous Zn batteries. ACS Energy Lett. **6**(8), 2704-2712 (2021). https://doi.org/10.1021/acsenergylett.1c01054

[S43] J. Yang, Z. Xu, J. Wang, L. Gai, X. Ji, et al. Antifreezing zwitterionic hydrogel electrolyte with high conductivity of 12.6 mS cm^{-1} at −40 °C through hydrated lithium ion hopping migration. Adv. Funct. Mater. **31**(18), (2021). https://doi.org/10.1002/adfm.202009438

[S44] J. Liu, C. Yang, X. Chi, B. Wen, W. Wang, et al. Water/sulfolane hybrid electrolyte achieves ultralow-temperature operation for high-voltage aqueous lithium-ion batteries. Adv. Funct. Mater. **32**(1), (2021). https://doi.org/10.1002/adfm.202106811

[S45] H. Lee, G. Jung, K. Keum, J.W. Kim, H. Jeong, et al. A textile-based temperature-tolerant stretchable supercapacitor for wearable electronics. Adv. Funct. Mater. **31**(50), 2106491 (2021). https://doi.org/10.1002/adfm.202106491

[S46] W. Sun, Z. Xu, C. Qiao, B. Lv, L. Gai, et al. Antifreezing proton zwitterionic hydrogel electrolyte via ionic hopping and grotthuss transport mechanism toward solid supercapacitor working at−50 °C. Adv. Sci. **9**(27), 2201679 (2022). https://doi.org/10.1002/advs.202201679

[S47] H. Wang, J. Liu, J. Wang, M. Hu, Y. Feng, et al. Concentrated hydrogel electrolyte-enabled aqueous rechargeable nico//Zn battery working from−20 to 50 °C. ACS Appl. Mater. Interfaces. **11**(1), 49-55 (2019). https://doi.org/10.1021/acsami.8b18003

[S48] F. Mo, G. Liang, Q. Meng, Z. Liu, H. Li, et al. A flexible rechargeable aqueous zinc manganese-dioxide battery working at−20 °C. Energy Environ. Sci. **12**(2), 706-715 (2019). https://doi.org/10.1039/c8ee02892c

[S49] Y. Chen, J. Zhao, Y. Wang. Quasi-solid-state zinc ion rechargeable batteries for subzero temperature applications. ACS Appl. Energy Mater. **3**(9), 9058-9065 (2020). https://doi.org/10.1021/acsaem.0c01452

[S50] Q. Zhang, Y. Ma, Y. Lu, L. Li, F. Wan, et al. Modulating electrolyte structure for ultralow temperature aqueous zinc batteries. Nat. Commun. **11**(1), 1-10 (2020). https://doi.org/10.1038/s41467-020-18284-0

[S51] N. Chang, T. Li, R. Li, S. Wang, Y. Yin, et al. An aqueous hybrid electrolyte for low-temperature zinc-based energy storage devices. Energy Environ. Sci. **13**(10), 3527-3535 (2020). https://doi.org/10.1039/d0ee01538e

[S52] M. Zhu, X. Wang, H. Tang, J. Wang, Q. Hao, et al. Antifreezing hydrogel with high zinc reversibility for flexible and durable aqueous batteries by cooperative hydrated cations. Adv. Funct. Mater. **30**(6), 1907218 (2020). https://doi.org/10.1002/adfm.201907218

[S53] J. Wang, Y. Huang, B. Liu, Z. Li, J. Zhang, et al. Flexible and anti-freezing zinc-ion batteries using a guar-gum/sodium-alginate/ethylene-glycol hydrogel electrolyte. Energy Storage Mater. **41**(599-605 (2021). https://doi.org/10.1016/j.ensm.2021.06.034

[S54] Q. Zhang, K. Xia, Y. Ma, Y. Lu, L. Li, et al. Chaotropic anion and fast-kinetics cathode enabling low-temperature aqueous Zn batteries. ACS Energy Lett. **6**(8), 2704-2712 (2021). https://doi.org/10.1021/acsenergylett.1c01054

[S55] Z. Chen, X. Li, D. Wang, Q. Yang, L. Ma, et al. Grafted mxene/polymer electrolyte for high performance solid zinc batteries with enhanced shelf life at low/high temperatures. Energy Environ. Sci. **14**(6), 3492-3501 (2021). https://doi.org/10.1039/d1ee00409c

[S56] Y. Zhao, Y. Lu, H. Li, Y. Zhu, Y. Meng, et al. Few-layer bismuth selenide cathode for low-temperature quasi-solid-state aqueous zinc metal batteries. Nat. Commun. **13**(1), 1-12 (2022). https://doi.org/10.1038/s41467-022-28380-y

[S57] L. Yang, L. Song, Y. Feng, M. Cao, P. Zhang, et al. Zinc ion trapping in a cellulose hydrogel as a solid electrolyte for a safe and flexible supercapacitor. J. Mater. Chem. A. **8**(25), 12314-12318 (2020). https://doi.org/10.1039/d0ta04360e

[S58] Z. Li, D. Chen, Y. An, C. Chen, L. Wu, et al. Flexible and anti-freezing quasi-solid-state zinc ion hybrid supercapacitors based on pencil shavings derived porous carbon. Energy Storage Mater. **28**, 307-314 (2020). https://doi.org/10.1016/j.ensm.2020.01.028

[S59] F. Li, L. Yu, Q. Hu, S. Guo, Y. Mei, et al. Fabricating low-temperature-tolerant and durable Zn-ion capacitors via modulation of co-solvent molecular interaction and cation solvation. Sci. China Mater. **64**(7), 1609-1620 (2021). https://doi.org/10.1007/s40843-020-1570-5

[S60] Y. Sun, H. Ma, X. Zhang, B. Liu, L. Liu, et al. Salty ice electrolyte with superior ionic conductivity towards low-temperature aqueous zinc ion hybrid capacitors. Adv. Funct. Mater. **31**(28), 2101277 (2021). https://doi.org/10.1002/adfm.202101277

[S61] C. Wang, Z. Pei, Q. Meng, C. Zhang, X. Sui, et al. Toward flexible zinc-ion hybrid capacitors with superhigh energy density and ultralong cycling life: The pivotal role of $ZnCl_2$ salt-based electrolytes. Angew. Chem. Int. Ed. **60**(2), 990-997 (2021). https://doi.org/10.1002/anie.202012030

[S62] J. Liu, Z. Khanam, S. Ahmed, T. Wang, H. Wang, et al. Flexible antifreeze Zn-ion hybrid supercapacitor based on gel electrolyte with graphene electrodes. ACS Appl. Mater. Interfaces. **13**(14), 16454-16468 (2021). https://doi.org/10.1021/acsami.1c02242
